# Molecular Characterization and Differential Expression of Olfactory Genes in the Antennae of the Black Cutworm Moth *Agrotis ipsilon*

**DOI:** 10.1371/journal.pone.0103420

**Published:** 2014-08-01

**Authors:** Shao-Hua Gu, Liang Sun, Ruo-Nan Yang, Kong-Ming Wu, Yu-Yuan Guo, Xian-Chun Li, Jing-Jiang Zhou, Yong-Jun Zhang

**Affiliations:** 1 State Key Laboratory for Biology of Plant Diseases and Insect Pests, Institute of Plant Protection, Chinese Academy of Agricultural Sciences, Beijing, China; 2 Department of Biological Chemistry and Crop Protection, Rothamsted Research, Harpenden, United Kingdom; 3 Key Laboratory of Integrated Management of Crop Diseases and Pests (Ministry of Education), College of Plant Protection, Nanjing Agricultural University, Nanjing, China; Plant and Food Research, New Zealand

## Abstract

Insects use their sensitive and selective olfactory system to detect outside chemical odorants, such as female sex pheromones and host plant volatiles. Several groups of olfactory proteins participate in the odorant detection process, including odorant binding proteins (OBPs), chemosensory proteins (CSPs), odorant receptors (ORs), ionotropic receptors (IRs) and sensory neuron membrane proteins (SNMPs). The identification and functional characterization of these olfactory proteins will enhance our knowledge of the molecular basis of insect chemoreception. In this study, we report the identification and differential expression profiles of these olfactory genes in the black cutworm moth *Agrotis ipsilon*. In total, 33 OBPs, 12 CSPs, 42 ORs, 24 IRs, 2 SNMPs and 1 gustatory receptor (GR) were annotated from the *A. ipsilon* antennal transcriptomes, and further RT-PCR and RT-qPCR revealed that 22 OBPs, 3 CSPs, 35 ORs, 14 IRs and the 2 SNMPs are uniquely or primarily expressed in the male and female antennae. Furthermore, one OBP (*AipsOBP6*) and one CSP (*AipsCSP2*) were exclusively expressed in the female sex pheromone gland. These antennae-enriched OBPs, CSPs, ORs, IRs and SNMPs were suggested to be responsible for pheromone and general odorant detection and thus could be meaningful target genes for us to study their biological functions *in vivo* and *in vitro*.

## Introduction

Insects use their sensitive and selective antennae, which express various olfactory proteins, to detect air borne odorant molecules, such as sex pheromones and plant volatiles. Species-specific pheromone molecules and general plant volatiles enter the sensillum lymph of the different types of antennae sensilla via the multipores of the insect cuticle [1], [2]. During the last 30 years, our knowledge of the molecular and cellular basis of insect chemoreception has greatly expanded. It is commonly accepted that several different groups of antennae-enriched olfactory proteins participate in the first stage of the detection of olfactory signals, including odorant binding proteins (OBPs), chemosensory proteins (CSPs), odorant receptors (ORs), ionotropic receptors (IRs) and sensory neuron membrane proteins (SNMPs) [3].

Insect OBPs are small water-soluble olfactory proteins that are presumed to be synthesized by non-neuronal auxiliary cells (trichogen and tormogen cells) of the sensory neurons and secreted into the sensillum lymph in high concentrations (up to 10 mM) [4]–[7]. The insect OBPs are commonly believed act as carrier proteins to transport odorants to the olfactory receptors. Functional studies of insect OBPs at both molecular and behavior levels have proven that insect OBPs are indispensable in insect chemoreception. For example, *Drosophila* OBP LUSH is required for the activation of pheromone-sensitive chemosensory neurons by the pheromone 11-*cis* vaccenyl acetate (cVA) [8], [9]. Additionally, in the fire ant *Solenopsis invicta*, the pheromone binding protein gene *Gp-9* regulates the colony social organization between the monogyne social form (with a single queen) and the polygyne form (with multiple queens) [10].

Insect CSPs, which were also called OS-D like proteins [11] or sensory appendage proteins (SAPs) [12], represent one novel group of olfactory proteins that are involved in insect olfaction. These proteins have shown broad expression profiles in chemosensory tissues, including antennae [13]–[17], maxillary palps [18], labial palps [18], [19] and proboscis [20]. However, these proteins are also found in non-chemosensory organs, such as legs [21], [22], wings [23], [24] and pheromone glands [15]. Functional studies of insect CSPs revealed that these proteins have multiple-functions in insect chemoreception, growth and development. For example, in the tsetse fly *Glossina morsitans morsitans*, the female antennae-enriched CSP transcripts were showed remarkable expression levels after a blood meal, which suggested that these proteins participate in the female host-seeking behavior [14]. In the American cockroach *Periplaneta americana*, one CSP homologous gene named *P10* was expressed 30 times higher in regenerating legs than in normal legs, which indicated that the *P10* gene had a putative function in the regeneration of insect legs [21], [22]. In the migratory locust *Locusta migratoria*, the antennae-expressed CSP gene has been proposed to regulate the rapid switch between attraction and repulsion behaviors [25].

The insect odorant receptors (ORs) are odorant-gated ion channels which composed of one odorant-binding subunit and the olfactory coreceptor Orco [26], [27]. The functional study of insect ORs, particularly the pheromone receptors (PRs), revealed their essential role in insect olfaction [28], [29]. The classical method to identify and annotate insect OR genes is through bioinformatic screenings of genomic sequences. At present, using this method, insect OR genes have been identified and annotated from various insect species, including *Drosophila melanogaster* [30]–[32], *Anopheles gambiae* [33], *Aedes aegypti* [34], *Apis mellifera* [35], *Nasonia vitripennis* [36], *Bombyx mori* [37], *Tribolium castaneum* [38], and *Acyrthosiphon pisum* [39].

Recently, a novel chemosensory receptor family called ionotropic receptors (IRs) was discovered in *D. melanogaster* [40]. In total, 66 IRs, which included two putative conserved coreceptors, IR25a and IR8a, were identified by screening *D. melanogaster* genomic data [41]. The expression analysis revealed that 15 DmelIR genes were specially expressed in the antennae [40]. The misexpression of DmelIR84a and DmelIR92a conferred ectopic olfactory responses to the electrophysiology-activated compounds phenylacetaldehyde and ammonia, respectively [40]. Thus far, different IR genes have been identified and annotated in various insect species, including *D. melanogaster* [40], *B. mori* [41], *Spodoptera littoralis* [42], *A. gambiae* [43], *Manduca sexta* [44], *Cydia pomonella* [45], and *Helicoverpa armigera* [46].

Previously, functional studies of insect olfactory genes primarily focused on model species, such as *D. melanogaster* and *B. mori*, whose genomic data are available. However, the functional studies of olfactory genes of other insect species have been restricted due the deficiency of the genomic data for these species. Recently, the high-throughput sequencing of antennae and other tissues have proved to be an efficient strategy for identifying and annotating different types of olfactory genes in various insect species, including *A. gambiae* [43], *M. sexta* [44], *C. pomonella* [45], *H. armigera* [46], *Cotesia vestalis* [47], *Agrilus planipennis* [48], *Aphis gossypii* [49], *S. littoralis* [50], *Ips typographus* and *Dendroctonus ponderosae* [51].

In the present study, using a next-generation sequencing (NGS) 454 GS FLX platform, we have identified and annotate several families of chemosensory genes (including OBPs, CSPs, ORs, IRs and SNMPs) from the antennae of the black cutworm moth *Agrotis ipsilon* (Hufnagel) (Lepidoptera: Noctuidae), which is known as a destructive pest of many crops [52]–[53]. Using semi-quantitative RT-PCR and real-time quantitative-PCR (RT-qPCR), we have screened a number of antennae-specific or enriched olfactory genes from the *A. ipsilon* antennal transcriptomes, which may play important functions in the chemoreception of *A. ipsilon.*

## Results and Discussion

### 454 sequencing and *de novo* assembly

Two non-normalized cDNA libraries of the male and female *A. ipsilon* antennae were constructed. After a single sequencing run using the 454 GS FLX platform, a total of 551388 (mean length 539 bp) and 537572 raw reads (mean length 548 bp) were produced from the male and female antennae samples, respectively. After trimming adaptor sequences, contaminating sequences and low quality sequences, 550456 (mean length 531 bp) and 536474 clean reads (mean length 540 bp) from male and female antennae, respectively, remained for the following assembly.

**Figure 1:**
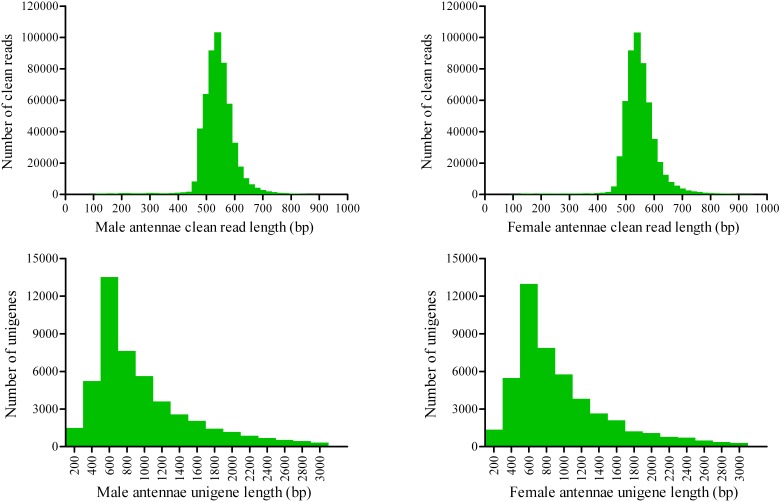
The size distribution of the clean reads and assembled unigenes from *A. ipsilon* male and female antennal transcriptomes.

All clean reads from male and female antennae were assembled and produced 40126 (mean length 1072 bp) and 41358 (mean length 1054 bp) unigenes, respectively. Furthermore, we assembled all clean reads from male and female antennae together and finally generated 48795 unigenes. Among these unigenes, 41173 are contigs (84.4%) and 7622 are singletons (15.6%). The assembled unigene lengths ranged from 100 bp to 15432 bp, with an average length of 967 bp. The size distribution of the assembled unigenes is shown in Figure 1. An overview of the sequencing and assembly process is presented in Table 1.

**Table 1:** An overview of the sequencing and assembly process.

	Male	Female	Total
Raw reads	551388	537572	1088960
Clean read	550456	536474	1086930
Clean read mean length	531 bp	540 bp	535.5 bp
Singletons	3583	4039	7622
Contigs	36543	37319	41173
Unigenes	40126	41358	48795
Unigene mean length	1072 bp	1054 bp	967 bp

### Homology searching of *A. ipsilon* antennal unigenes with other insect species

**Figure 2:**
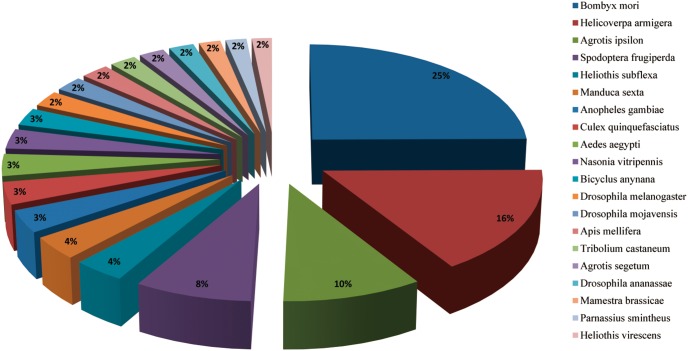
Top 20 best hits of the BLASTn results. All *A. ipsilon* antennal unigenes were used in BLASTn to search the GenBank entries. The best hits with an E-value < = 1.0E-5 for each query were grouped according to species.

We search for homologs in other insect species using the BLASTx and BLASTn programs with the e-value cut-off of 10e-5 [54]. The results indicated that 25180 of the 48795 unigenes (51.6%) had BLASTx hits in the non-redundant protein (nr) databases and that 17947 unigenes (36.8%) had BLASTn hits in the non-redundant nucleotide sequence (nt) databases. Some unigenes are homologous to more than one species. Most annotated *A. ipsilon* antennal unigenes have the best hits with Lepidoptera insect genes (8542 of the 17947 nt-hit unigenes); the highest hits included 2818 unigenes that were homologous to *B. mori* genes, 1820 unigenes that were homologous to *H. armigera* genes. The second highest hits are with Dipteran species genes, with 276 hits of *D. melanogaster* genes, and 392 and 383 hits that were homologous to genes of the mosquitoes *A. gambiae* and *A. aegypti*, respectively. The other unigenes were found to be homologous to genes from the wasp *N. vitripennis* (348 hits), the beetle *T. castaneum* (244 hits) and from the western honey bee *A. mellifera* (261 hits) (Figure 2).

### Functional annotation of the *A. ipsilon* antennal unigenes

**Figure 3:**
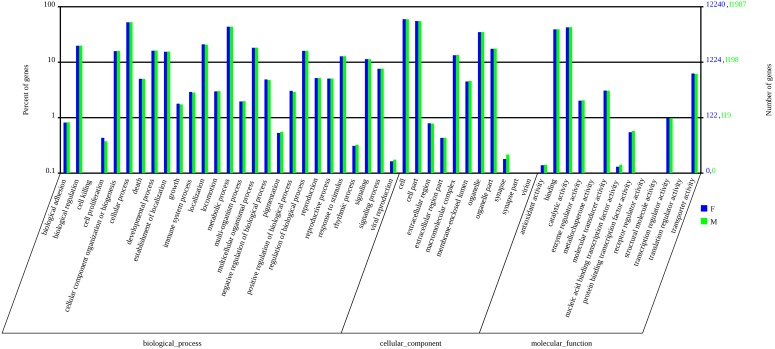
Gene Ontology (GO) classifications of the male and female *A. ipsilon* antennal unigenes according to their involvement in biological processes, cellular component and molecular function.

Similar to those genes that were found in the antennal transcriptomes of *M. sexta* [44], *S. littoralis* [55] and *H. armigera* [46], most *A. ipsilon* antennal unigenes (approximately 72%) could not be assigned to a Gene Ontology (GO) category. In total, 11987 male antennal unigenes and 12240 female antennal unigenes were annotated into different functional groups (biological process, cellular components and molecular functions) according to GO analysis [56] (Figure 3). Some transcripts were annotated into more than one GO category. The numbers of each GO category were similar between the male and female antennal transcriptomes (Figure 3). The cellular process (6301 male antennal unigenes and 6425 female antennal unigenes) and metabolic process (5243 male antennal unigenes and 5349 female antennal unigenes) GO categories were most abundantly represented within the biological process GO ontology. In the cellular components GO ontology, the transcripts were primarily distributed in the cell (7148 male antennal unigenes and 7308 female antennal unigenes) and in cell part (6619 male antennal unigenes and 6752 female antennal unigenes). The GO analysis also showed that the binding (4705 male antennal unigenes and 4787 female antennal unigenes) and catalytic activity (5133 male antennal unigenes and 5210 female antennal unigenes) were most abundant in the molecular function ontology (Figure 3).

### Chemosensory genes are highly abundant in the *A. ipsilon* antennae

Because a non-normalized cDNA library was used for 454 sequencing in this study, the number of reads of per unigene can represent the relative mRNA abundance in the *A. ipsilon* antennal transcriptomes. Among the top 500 most highly abundant transcripts, 89 transcripts are annotated as olfactory genes, which suggests their involvement in insect chemosensory reception, including olfactory receptors, odorant-binding proteins, chemosensory proteins, antennal cytochrome P450s, antennal-enriched UDP-glycosyltransferases, antennal oxidoreductases, antennal aldehyde oxidases, sensory neuron membrane proteins and takeout-like proteins (Table S1).

### Candidate odorant binding proteins in the *A. ipsilon* antennae

**Figure 4:**
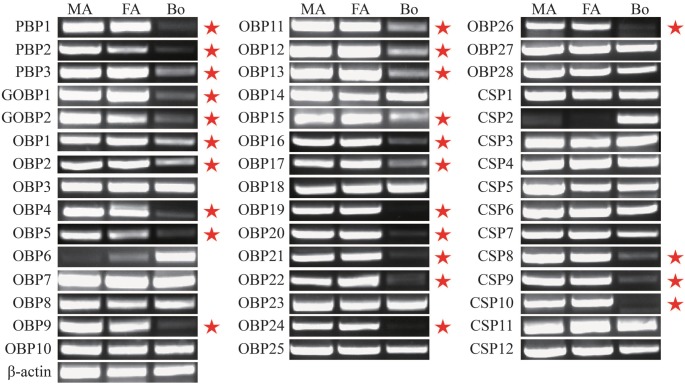
*A. ipsilon* OBP and CSP transcript levels in different tissues as evaluated by RT-PCR. MA: male antennae; FA: female antennae; Bo: body. Pheromone gland rather than body was used in the analysis of *AipsOBP6* and *AipsCSP2*. Antennae specific or enriched genes are labeled with a red pentagram. *β-actin* was used as an internal reference gene to test the integrity of each cDNA template; the similar intensity of *β-actin* bands among different tissues indicates the use of equal template concentrations.

OBPs are believed to be involved in the initial biochemical recognition steps in insect odorant perception by capturing and transporting odorant molecules to the olfactory receptors (ORs) [57]–[59]. In the *A. ipsilon* antennal transcriptomes, a total of 33 OBP genes were annotated (Table 2) based on the tBLASTn results. The number of *A. ipsilon* OBP identified in present study is a little fewer than the number identified from the genome of *B. mori* (44) [60], *A. gambiae* (57) [61] and *D. melanogaster* (51) [62], so there may still some OBP genes are not identified from the *A. ipsilon* antennae due to their low expression level. Among the identified 33 OBP genes, 28 have intact ORFs with lengths ranging from 402 bp to 759 bp. The RPKM value analysis revealed that 9 OBP genes (*PBP1, PBP2, PBP3, GOBP1, GOBP2, OBP4, OBP11, OBP18* and *OBP24*) are highly abundant in the male and female antennal transcriptomes (RPKM>1000) (Table 2). The RT-PCR results indicated that 22 OBP genes (*PBP1, PBP2, PBP3, GOBP1, GOBP2, OBP1, OBP2, OBP4, OBP5, OBP9, OBP11, OBP12, OBP13, OBP15, OBP16, OBP17, OBP19, OBP20, OBP21, OBP22, OBP24* and *OBP26*) are uniquely or primarily expressed in the male and female antennae (Figure 4). Based on the different expression profiles of these OBPs in male and female antennae, we suggest these male antennae-enriched expressed OBPs are involved in sex pheromone detection, whereas female antennae-enriched expressed OBPs play important roles in locating suitable host plants and oviposition sites.

**Table 2:** List of OBP genes in *A. ipsilon* antennae.

Unigene	Gene	Length(bp)	ORF(bp)	BLASTx annotation	Score	*E*-value	%Identify	RPKM value	
								Male	Female
Unigene_5952	PBP1	1182	513	gb|AFM36756.1| pheromone-binding protein 1 [Agrotis ipsilon]	353	2e-122	100%	5829	18523
Unigene_10109	PBP2	968	498	gb|AFM36757.1| pheromone-binding protein 2 [Agrotis ipsilon]	338	1e-116	100%	9759	28502
Unigene_19893	PBP3	2391	495	gb|AFM36758.1| pheromone-binding protein 3 [Agrotis ipsilon]	340	1e-117	100%	1151	8754
Unigene_14658	GOBP1	2153	495	gb|AFM36759.1| general odorant-binding protein 1 [Agrotis ipsilon]	299	3e-101	100%	3948	2977
Unigene_33505	GOBP2	1087	489	gb|AFM36760.1| general odorant-binding protein 2 [Agrotis ipsilon]	295	6e-100	100%	3136	2004
Unigene_7317	OBP1	765	543	gb|ACX53761.1| odorant binding protein [Heliothis virescens]	178	3e-53	53%	49	83
Unigene_6175	OBP2	579	447	gb|AAL66739.1| AF461143_1 pheromone binding protein 4 [Mamestra brassicae]	243	6e-79	80%	995	624
Unigene_3156	OBP3	743	588	gb|AFM93773.1| odorant-binding protein 19 [Helicoverpa armigera]	355	1e-38	52%	18	37
Unigene_9245	OBP4	1025	453	gb|AEB54591.1| OBP7 [Helicoverpa armigera]	190	4e-56	63%	2322	2143
Unigene_5755	OBP5	1124	414	gb|ACX53795.1| odorant binding protein [Heliothis virescens]	169	4e-51	71%	793	748
Unigene_8140	OBP6	378	---	gb|ACX53743.1| odorant binding protein [Heliothis virescens]	231	1e-75	86%	1	1
Unigene_31090	OBP7	896	438	gb|AEB54587.1| OBP6 [Helicoverpa armigera]	99.4	4e-22	43%	23	14
Unigene_36919	OBP8	1665	507	gb|ADO95155.1| antennal binding protein 7 [Antheraea yamamai]	66.6	8e-10	34%	56	34
Unigene_15755	OBP9	423	---	gb|AFM77984.1| odorant binding protein 6 [Spodoptera exigua]	192	4e-60	62%	16	21
Unigene_29151	OBP10	234	---	ref|NP_001157372.1| odorant binding protein [Bombyx mori]	41.2	1e-06	36%	4	4
Unigene_5992	OBP11	1474	726	ref|NP_001157372.1| odorant binding protein [Bombyx mori]	223	2e-67	44%	3388	3289
Unigene_6859	OBP12	3153	441	gb|AFG72998.1| odorant-binding protein 1 [Cnaphalocrocis medinalis]	183	4e-56	57%	39	19
Unigene_8851	OBP13	1051	420	gb|AEB54589.1| OBP8 [Helicoverpa armigera]	215	4e-69	83%	360	93
Unigene_6275	OBP14	1086	759	gb|ADD71058.1| odorant-binding protein [Chilo suppressalis]	354	2e-118	63%	306	861
Unigene_9200	OBP15	1635	429	gb|AEB54586.1| OBP2 [Helicoverpa armigera]	254	1e-78	82%	149	299
Unigene_36163	OBP16	1694	516	gb|ADO95155.1| antennal binding protein 7 [Antheraea yamamai]	53.5	7e-07	32%	52	36
Unigene_8227	OBP17	1332	423	gb|EHJ65654.1| antennal binding protein 4 [Danaus plexippus]	176	4e-54	67%	211	161
Unigene_6670	OBP18	791	402	gb|AFI57166.1| odorant-binding protein 17 [Helicoverpa armigera]	248	3e-80	90%	2269	2253
Unigene_8505	OBP19	1351	426	gb|AEB54588.1| OBP13 [Helicoverpa armigera]	238	1e-73	87%	1011	339
Unigene_3387	OBP20	848	465	gb|EFA09155.1| odorant binding protein 22 [Tribolium castaneum]	52.4	1e-05	38%	574	456
Unigene_6261	OBP21	721	435	gb|AEB54592.1| OBP9 [Helicoverpa armigera]	152	9e-43	53%	729	446
Unigene_34247	OBP22	1206	414	emb|CAA05508.1| antennal binding protein X [Heliothis virescens]	202	3e-60	83%	80	339
Unigene_24788	OBP23	569	438	gb|AEB54581.1| OBP5 [Helicoverpa armigera]	201	3e-63	68%	72	86
Unigene_33130	OBP24	1922	501	gb|ADY17882.1| odorant binding protein [Spodoptera exigua]	252	3e-80	73%	1072	1328
Unigene_37860	OBP25	357	---	gb|AAR28763.1| odorant-binding protein-2 precursor [Spodoptera frugiperda]	125	4e-31	50%	4	7
Unigene_13942	OBP26	569	450	gb|AEB54581.1| OBP5 [Helicoverpa armigera]	169	6e-50	57%	29	13
Unigene_23138	OBP27	495	---	emb|CAR85645.1| odorant-binding protein 4, partial [Myzus persicae]	152	5e-40	44%	15	13
Unigene_4326	OBP28	823	642	ref|NP_001159621.1| odorant binding protein [Bombyx mori]	38.1	1.5	28%	306	216

“---” represent that gene is partial and has not intact ORF. The nucleotide sequences of all 33 OBP genes are listed in Table S2.

**Figure 5:**
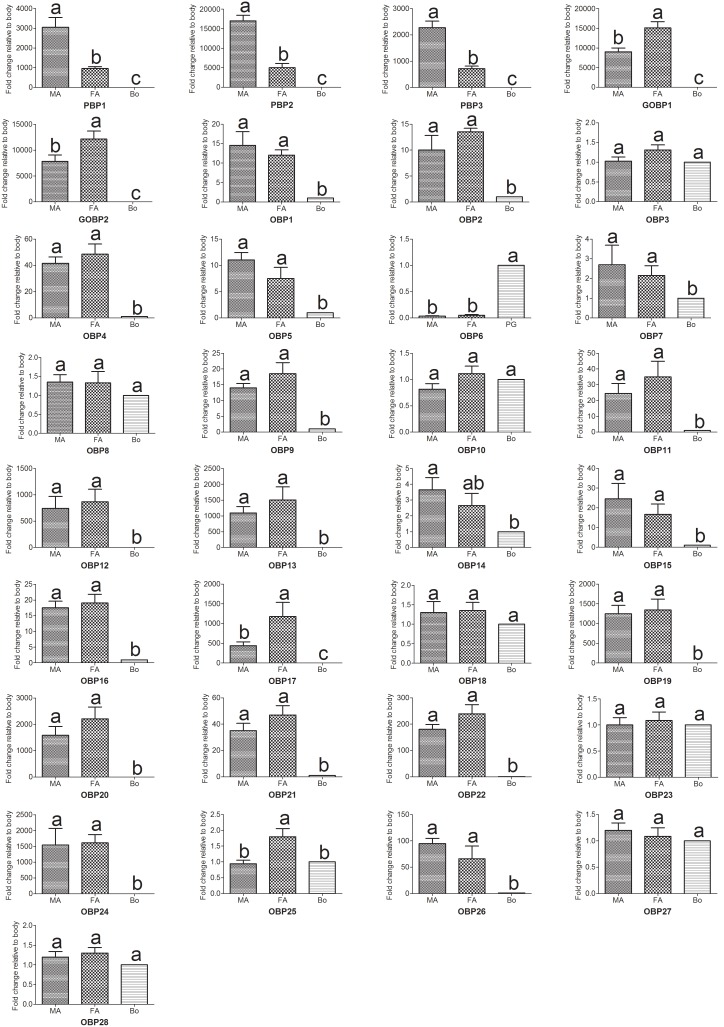
*A. ipsilon* OBP transcript levels in different tissues as measured by RT-qPCR. MA: male antennae; FA: female antennae; Bo: body. Pheromone gland rather than body was used in the analysis of *AipsOBP6*. The internal controls *β-actin* and *ribosomal protein S3* were used to normalize transcript levels in each sample. This figure was presented using *β-actin* as the reference gene to normalize the target gene expression and to correct sample-to-sample variation; similar results were obtained with *ribosomal protein S3* as the reference gene. The standard error is represented by the error bar, and the different letters (a, b, c) above each bar denote significant differences (p<0.05).

Furthermore, real-time quantitative PCR (RT-qPCR) analysis was performed to compare the accurate quantitative expression levels of these OBP genes among different tissues between sexes, and the results suggested that the three PBP genes (*PBP1, PBP2* and *PBP3*) are expressed higher in the male antennae than in the female antennae (*p*<0.01) (Figure 5). However, the RT-qPCR results lack concordance with the RPKM values, this reason may be the sequencing depth of 454 is not good enough. PBP1 and PBP2 showed high binding affinities with the two main sex pheromones of *A. ipsilon*, whereas PBP3 specifically binds to the minor amount sex pheromone Z11-16: Ac with a high binding ability [63]. In contrast, the expression levels of *GOBP1, GOBP2* and *OBP17* were much higher in the female antennae than in the male antennae (Figure 5). Interestingly, one OBP (*OBP6*) was primarily expressed in the pheromone gland (PG) (Figure 4 and Figure 5); this result was also reported in another study [64]. Unlike the common antennae-enriched OBPs, this PG-expressed OBP may play a different role in odorant and pheromone detection and transportation.

### Candidate chemosensory proteins in the *A. ipsilon* antennae

**Figure 6:**
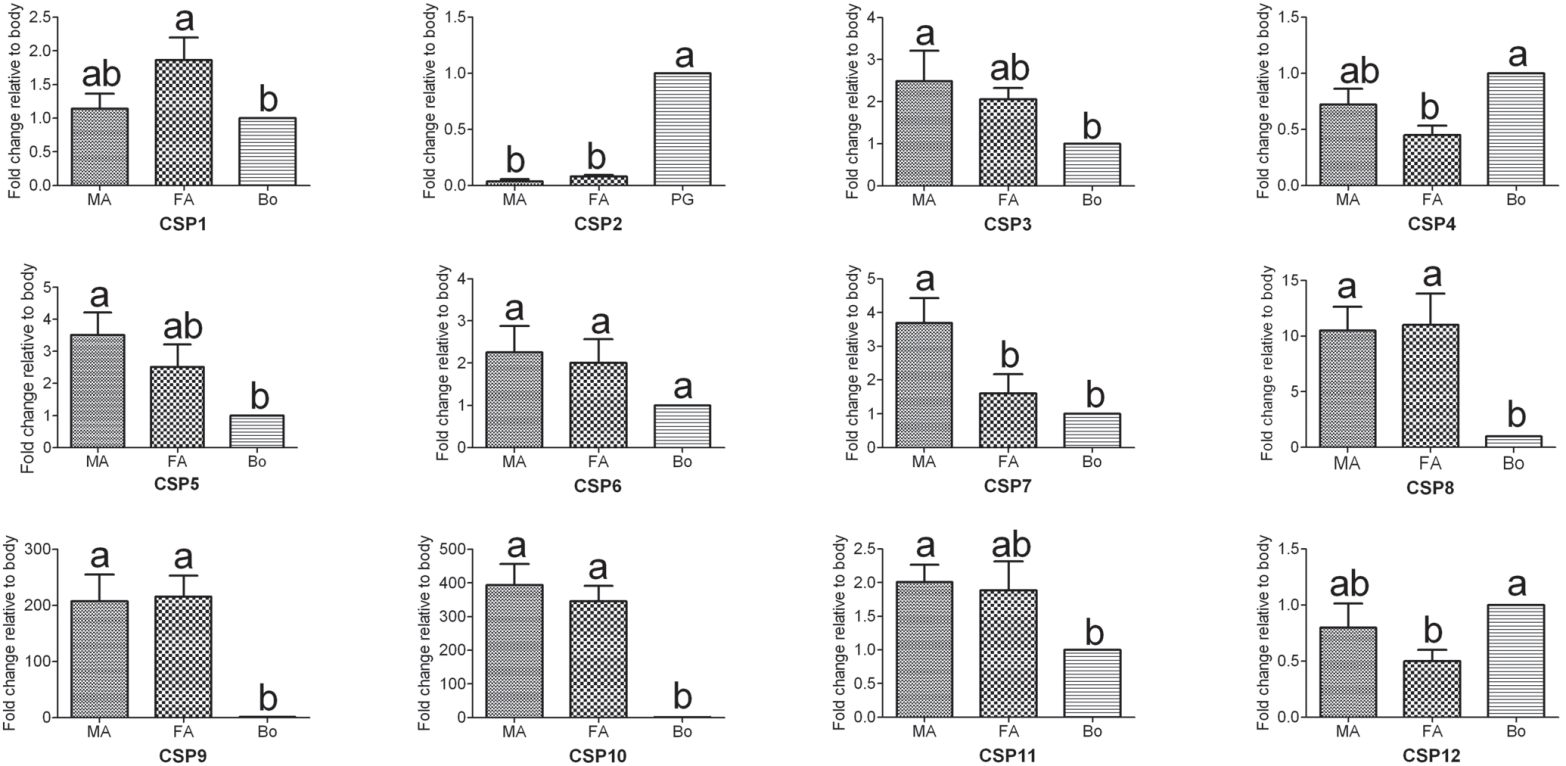
*A. ipsilon* CSP transcript levels in different tissues as measured by RT-qPCR. MA: male antennae; FA: female antennae; Bo: body. Pheromone gland rather than body was used in the analysis of *AipsCSP2*. The internal controls *β-actin* and *ribosomal protein S3* were used to normalize transcript levels in each sample. This figure was presented using *β-actin* as reference gene to normalize the target gene expression and correct sample-to-sample variation; similar results were obtained with *ribosomal protein S3* as the reference gene. The standard error is represented by the error bar, and the different letters (a, b) above each bar denote significant differences (p<0.05).

Chemosensory proteins (CSPs) represent a new class of soluble carrier proteins in the lymph of insect antennal chemosensilla and they are proposed to play similar functions as OBPs in insect chemoreception [65]. In this study, we have identified 12 novel CSP genes in the *A. ipsilon* antennae (Table 3). Based on the extensive expression profiles of CSPs, the remaining CSPs which expressed in other tissues such as legs and wings may not be identified in present study. In total, 11 of the novel genes had intact ORFs, and the protein sequences had the typical four conserved cysteines, which are recognized as the signature feature of insect CSPs [65]. The RPKM value analysis revealed that 4 CSP genes (*CSP4, CSP7, CSP9* and *CSP10*) are highly abundant in the male and female antennal transcriptomes (RPKM>1000) (Table 3). The RT-PCR and RT-qPCR results indicated that 3 CSP genes (*CSP8, CSP9* and *CSP10*) are highly expressed in the male and female antennae (Figure 4 and Figure 6). This result suggested that these three antennae-enriched CSPs might play essential roles in the chemical communication process in insects. Interestingly, one CSP gene (*CSP2*) was not expressed in the antennae but was specifically expressed in the female pheromone gland (PG) (Figure 4 and Figure 6). CSPs that are expressed in the pheromone gland of the cabbage armyworm *M. brassicae* can bind sex pheromone analogs, which suggests that these CSPs may play a role in pheromone capture [15]. In *Heliothis virescens* and *B. mori,* CSPs are all detected in the pheromone gland [66]–[68]. This observation suggests the possible involvement of these proteins in carrying and releasing sex pheromones, as demonstrated for the antennal OBPs and CSPs. The insect may use these female PG-enriched OBPs and CSPs to auto-detect and monitor the sex pheromones released by themselves [69]–[70].

**Table 3:** List of CSP genes in *A. ipsilon* antennae.

Unigene	Gene	Length(bp)	ORF(bp)	BLASTx annotation	Score	*E*-value	%Identify	RPKM value	
								Male	Female
Unigene_32747	CSP1	479	375	gb|ACX53825.1| chemosensory protein [Heliothis virescens]	125	1e-33	47%	171	155
Unigene_10019	CSP2	585	360	dbj|BAF91716.1| chemosensory protein [Papilio xuthus]	159	8e-48	66%	6	9
Unigene_32521	CSP3	927	387	gb|AAF71290.2|AF255919_1 chemosensory protein[Mamestra brassicae]	224	6e-70	82%	66	74
Unigene_5484	CSP4	653	363	gb|AEX07265.1| CSP2 [Helicoverpa armigera]	221	2e-70	86%	2246	2979
Unigene_4019	CSP5	823	324	gb|EHJ67380.1| chemosensory protein [Danaus plexippus]	185	3e-57	84%	25	31
Unigene_6911	CSP6	1762	384	gb|AAM77040.1| chemosensory protein 2 [Heliothis virescens]	225	3e-67	87%	279	571
Unigene_33786	CSP7	980	387	gb|AAP57460.1| chemosensory protein [Agrotis ipsilon]	218	1e-67	98%	1658	1327
Unigene_4517	CSP8	702	372	gb|ACX53806.1| chemosensory protein [Heliothis virescens]	210	7e-66	76%	159	72
Unigene_33739	CSP9	1617	447	gb|ABM67686.1| chemosensory protein CSP1 [Plutella xylostella]	173	3e-52	65%	1756	1371
Unigene_37440	CSP10	799	366	gb|ACX53813.1| chemosensory protein [Heliothis virescens]	197	2e-59	85%	1821	1921
Unigene_7374	CSP11	1186	897	ref|NP_001037069.1| chemosensory protein 9 precursor [Bombyx mori]	236	1e-70	76%	539	414
Unigene_16782	CSP12	360	---	gb|AFR92094.1| chemosensory protein 10 [Helicoverpa armigera]	202	1e-63	80%	4	10

“---” represent that gene is partial and has not intact ORF. The nucleotide sequences of all 12 CSP genes are listed in Table S2.

### Candidate olfactory receptors in the *A. ipsilon* antennae

**Figure 7:**
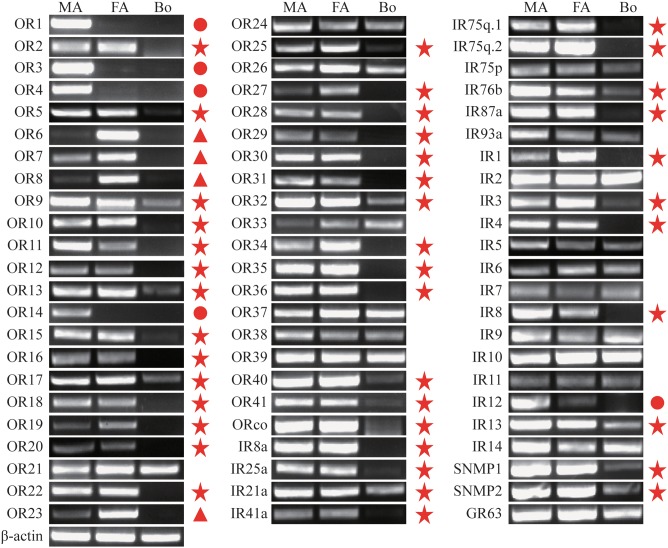
*A. ipsilon* OR, IR, SNMP and GR transcript levels in different tissues as evaluated by RT-PCR. MA: male antennae; FA: female antennae; Bo: body. Genes that are equally expressed in the male and female antennae are labeled with a red pentagram. Genes that are specifically or primarily expressed in the male antennae are labeled with a red circle. Genes that are specifically or primarily expressed in the female antennae are labeled with a red triangle. *β-actin* was used as the internal reference gene to test the integrity of each cDNA templates; the similar intensity of *β-actin* bands among different tissues indicates the use of equal template concentrations.

**Figure 8:**
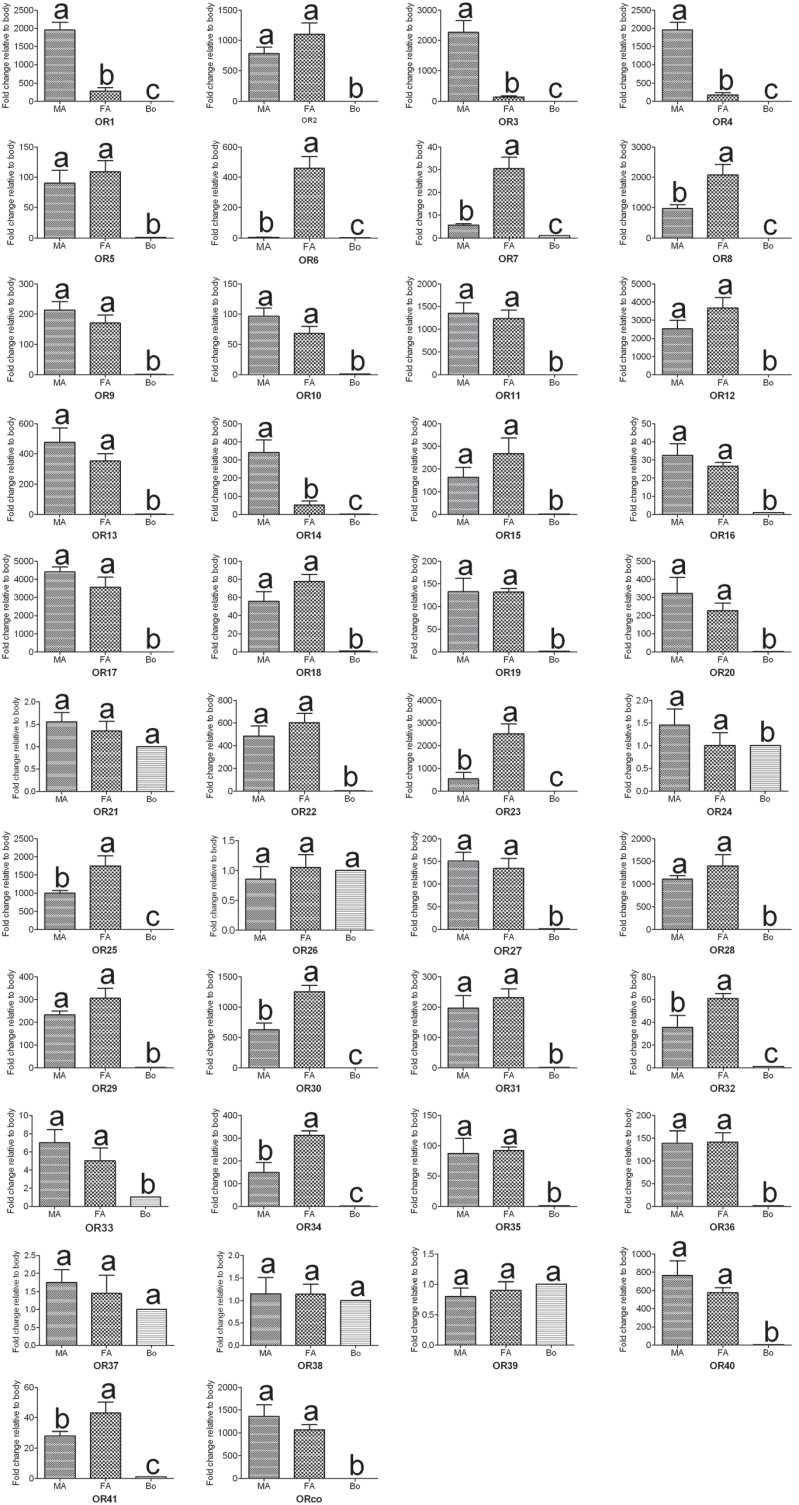
*A. ipsilon* OR transcript levels in different tissues as measured by RT-qPCR. MA: male antennae; FA: female antennae; Bo: body. The internal controls *β-actin* and *ribosomal protein S3* were used to normalize transcript levels in each sample. This figure was presented using *β-actin* as the reference gene to normalize the target gene expression and to correct sample-to-sample variation; similar results were obtained with *ribosomal protein S3* as the reference gene. The standard error is represented by the error bar, and the different letters (a, b, c) above each bar denote significant differences (p<0.05).

**Figure 9:**
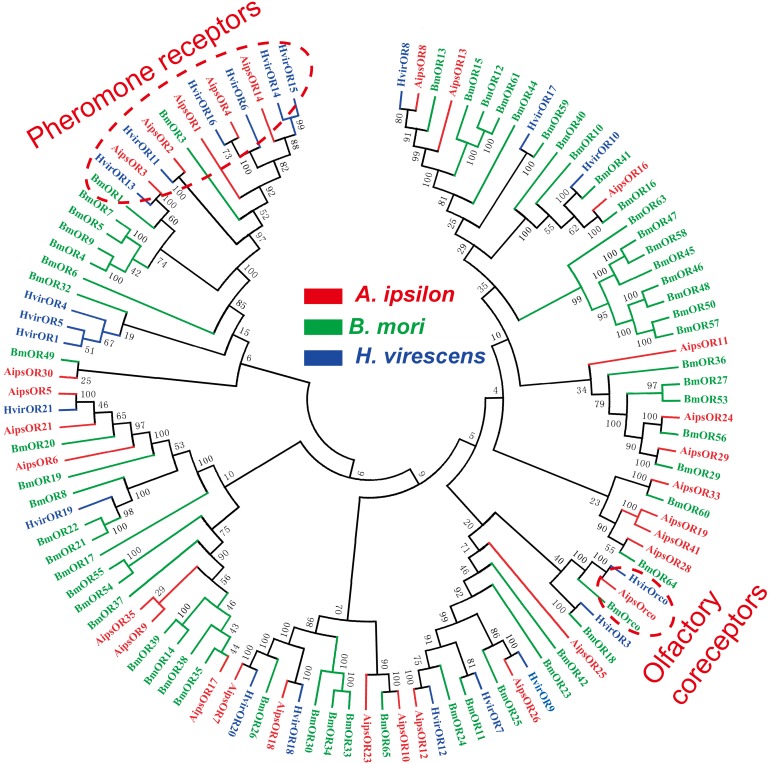
Neighbor-joining tree of candidate odorant receptor proteins from *A. ipsilon* (red), *B. mori* (green) and *H. virescens* (blue). The protein names and sequences of ORs that were used in this analysis are listed in Table S5.

Insect olfactory receptors (ORs) are the most important players in sex pheromone and general odorant detection. In the present study, we have identified 42 OR genes (41 typical ORs and one atypical coreceptor) from the *A. ipsilon* antennal transcriptomes (Table 4). In insect, the axons from the sensory neurons converge into glomeruli in the antennal lobe. There are 66 glomeruli in the antennae lobe of the male *A. ipsilon* moth [71], based on the hypothesis that the number of the glomeruli equals the number of olfactory receptors [72], [73], we predict there are about 24 OR genes still need to be identified. In total, 12 of the 42 ORs have intact ORFs. The RPKM value analysis revealed that the ORco had the highest expression level among the 42 ORs, with RPKM value of 741 and 997 in the male and female antennae, respectively. The other 41 typical ORs, however, showed a relative low expression level (RPKM ranged from 0 to 567) compared with the ORco, OBP and CSP genes. Three ORs (*OR1, OR3* and *OR4*) showed a higher RPKM in the male antennae than in the female antennae (more than 20 times) (Table 4). The RT-PCR and RT-qPCR results indicated that 35 ORs were exclusively or primarily expressed in the antennae. Among these ORs, 4 ORs (*OR1, OR3, OR4* and *OR14*) have male antennae-specific expression (Figure 7 and Figure 8), which suggests that these ORs may play essential roles in the detection of sex pheromones. In total, 4 ORs (*OR6, OR7, OR8* and *OR23*) have female antennae-enriched expression (Figure 7 and Figure 8), which suggests that these ORs may play important roles in the detection of general odorants, such as host plant volatiles. The OR tree from three Lepidoptera insects are extremely divergent; however, the olfactory coreceptor family and the pheromone receptor family are highly conserved (Figure 9).

**Table 4:** List of OR genes in *A. ipsilon* antennae.

Unigene	Gene	Length (bp)	ORF (bp)	BLASTx annotation	Score	*E*-value	% Identify	RPKM value	
								Male	Female
Unigene_225	OR1	1550	1308	emb|CAG38117.1| putative chemosensory receptor 16 [Heliothis virescens]	396	3e-131	54%	126	6
Unigene_6374	OR2	2121	1308	gb|ACF32965.1| olfactory receptor 11 [Helicoverpa armigera]	724	0.0	80%	34	13
Unigene_30282	OR3	1464	1272	dbj|BAG71423.2| olfactory receptor [Mythimna separata]	585	0.0	72%	567	3
Unigene_1457	OR4	1589	1299	gb|ACS45306.1| candidate odorant receptor 3 [Helicoverpa armigera]	606	0.0	69%	178	2
Unigene_13891	OR5	1314	1209	emb|CAG38122.1| putative chemosensory receptor 21 [Heliothis virescens]	561	0.0	74%	13	6
Unigene_14810	OR6	581	---	emb|CAG38122.1| putative chemosensory receptor 21 [Heliothis virescens]	157	1e-42	39%	26	0
Unigene_7733	OR7	1558	1179	gb|ACC63240.1| olfactory receptor 20, partial [Helicoverpa armigera]	617	0.0	74%	49	26
Unigene_10999	OR8	1594	1185	emb|CAD31949.1| putative chemosensory receptor 8 [Heliothis virescens]	503	2e-171	61%	27	13
Unigene_10668	OR9	1759	1251	ref|NP_001103476.1| olfactory receptor 35 [Bombyx mori]	436	2e-147	53%	19	5
Unigene_10397	OR10	1483	1290	gb|AFC91732.1| putative odorant receptor OR24 [Cydia pomonella]	459	4e-156	54%	14	16
Unigene_12603	OR11	480	---	ref|NP_001116817.1| olfactory receptor-like [Bombyx mori]	182	1e-73	84%	5	12
Unigene_14326	OR12	861	---	emb|CAG38113.1| putative chemosensory receptor 12 [Heliothis virescens]	512	1e-177	85%	13	8
Unigene_16749	OR13	495	---	ref|NP_001166603.1| olfactory receptor 13 [Bombyx mori]	165	1e-45	59%	6	6
Unigene_16622	OR14	1453	1248	dbj|BAG71414.1| olfactory receptor-1 [Mythimna separata]	624	0.0	71%	0	14
Unigene_24590	OR15	183	---	emb|CAG38111.1| putative chemosensory receptor 10 [Heliothis virescens]	127	8e-30	98%	14	8
Unigene_15088	OR16	693	---	gb|EHJ70341.1| olfactory receptor 16 [Danaus plexippus]	343	9e-115	73%	7	8
Unigene_12606	OR17	673	---	gb|AFL70813.1| odorant receptor 50, partial [Manduca sexta]	243	4e-75	60%	11	3
Unigene_2007	OR18	1080	---	gb|ACL81185.1| putative olfactory receptor 18 [Agrotis segetum]	660	0.0	98%	74	27
Unigene_18000	OR19	923	---	ref|NP_001166621.1| olfactory receptor 64 [Bombyx mori]	281	2e-89	56%	4	8
Unigene_28103	OR20	291	---	ref|NP_001166605.1| olfactory receptor 20 [Bombyx mori]	115	9e-28	62%	7	0
Unigene_15599	OR21	532	---	emb|CAG38122.1| putative chemosensory receptor 21 [Heliothis virescens]	168	4e-47	47%	10	7
Unigene_11593	OR22	138	---	gb|AFC91721.1| putative odorant receptor OR12 [Cydia pomonella]	80.5	4e-14	82%	12	10
Unigene_14448	OR23	915	---	gb|EHJ75140.1| olfactory receptor [Danaus plexippus]	173	1e-48	65%	62	17
Unigene_17502	OR24	675	---	ref|NP_001166617.1| olfactory receptor 56 [Bombyx mori]	424	4e-144	67%	9	2
Unigene_13261	OR25	744	---	gb|ACC63237.1| olfactory receptor 9 [Helicoverpa armigera]	89.0	5e-18	26%	12	7
Unigene_10818	OR26	648	---	emb|CAD31950.1| putative chemosensory receptor 9 [Heliothis virescens]	311	6e-130	79%	13	9
Unigene_21855	OR27	220	---	emb|CAG38118.1| putative chemosensory receptor 17 [Heliothis virescens]	85.5	1e-24	82%	5	5
Unigene_11342	OR28	411	---	ref|NP_001166621.1| olfactory receptor 64 [Bombyx mori]	137	2e-56	53%	6	12
Unigene_13860	OR29	1177	---	ref|NP_001166894.1| olfactory receptor 29 [Bombyx mori]	552	0.0	70%	13	115
Unigene_14601	OR30	645	---	gb|EEZ99413.1| odorant receptor 50 [Tribolium castaneum]	51.6	2e-04	25%	34	10
Unigene_21353	OR31	239	---	gb|ACF32961.1| olfactory receptor 3 [Helicoverpa armigera]	120	9e-37	79%	11	5
Unigene_28909	OR32	451	---	gb|AFC91724.1| putative odorant receptor OR16 [Cydia pomonella]	176	2e-50	60%	4	4
Unigene_12299	OR33	537	---	ref|NP_001155301.1| olfactory receptor 60 [Bombyx mori]	199	8e-104	71%	24	6
Unigene_13820	OR34	603	---	gb|AFL70813.1| odorant receptor 50, partial [Manduca sexta]	210	7e-63	58%	25	3
Unigene_13081	OR35	1380	1182	gb|AFL70813.1| odorant receptor 50, partial [Manduca sexta]	470	4e-160	56%	15	8
Unigene_25508	OR36	312	---	gb|AFC91738.1| putative odorant receptor OR30, partial [Cydia pomonella]	135	1e-36	61%	6	29
Unigene_27934	OR37	417	---	gb|EHJ67735.1| olfactory receptor [Danaus plexippus]	120	2e-30	45%	4	4
Unigene_15945	OR38	312	---	gb|ABK27848.1| odorant receptor 33 [Bombyx mori]	100	2e-22	58%	16	0
Unigene_22364	OR39	267	---	tpg|DAA05980.1| TPA_exp: odorant receptor 22 [Bombyx mori]	117	6e-38	63%	0	9
Unigene_18944	OR40	402	---	gb|AEF32141.1| odorant receptor [Spodoptera exigua]	199	1e-69	77%	10	0
Unigene_13402	OR41	540	---	ref|NP_001091818.1| olfactory receptor 42 [Bombyx mori]	238	2e-73	71%	6	20
Unigene_5611	ORco	3033	1422	dbj|BAG71415.1| olfactory receptor-2 [Mythimna separata]	969	0.0	97%	741	997

“---” represent that gene is partial and has not intact ORF. The nucleotide sequences of all 42 OR genes are listed in Table S2.

### Candidate ionotropic receptors in the *A. ipsilon* antennae

**Figure 10:**
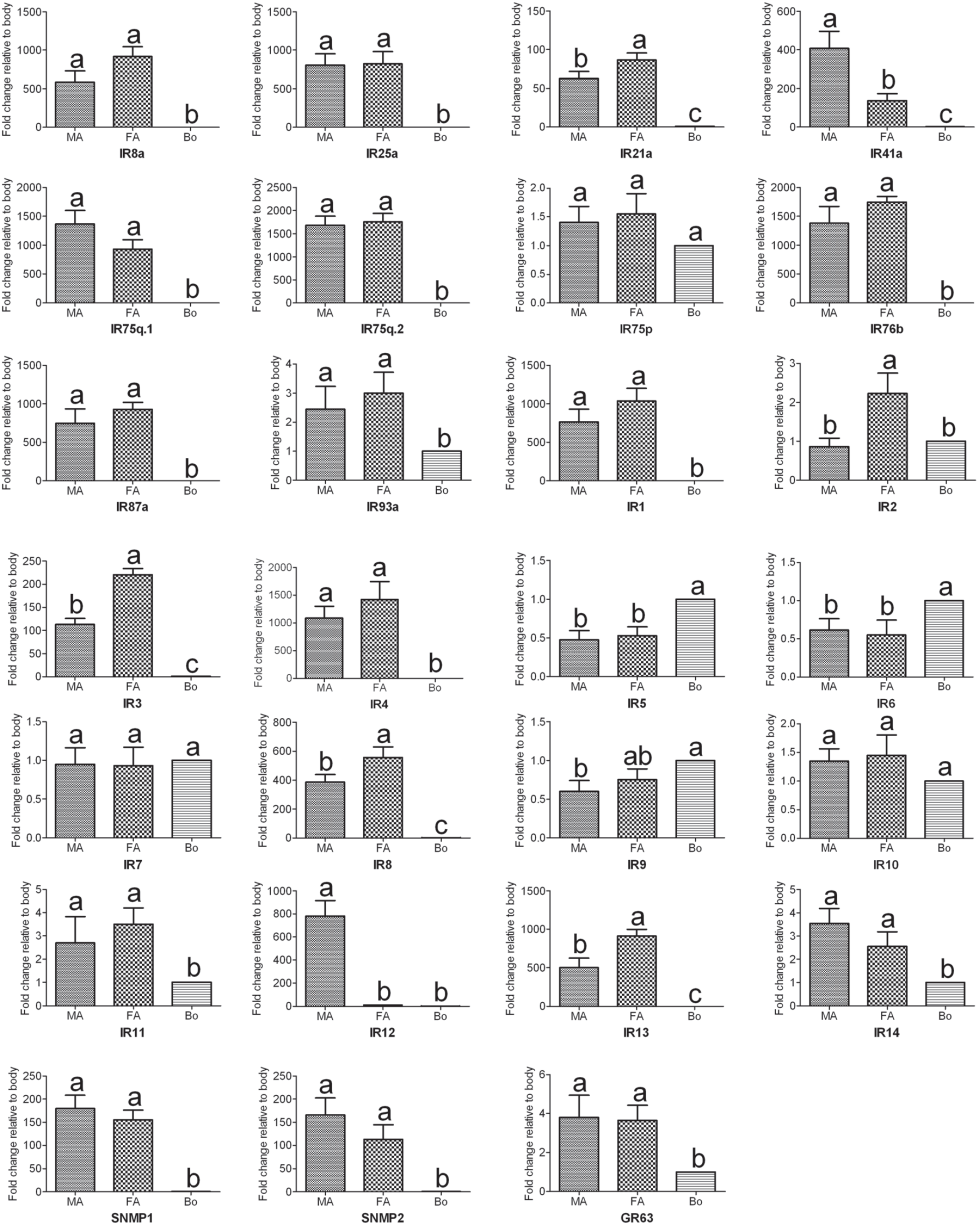
*A. ipsilon* IR, SNMP and GR transcript levels in different tissues as measured by RT-qPCR. MA: male antennae; FA: female antennae; Bo: body. The internal controls *β-actin* and *ribosomal protein S3* were used to normalize transcript levels in each sample. This figure was presented using *β-actin* as the reference gene to normalize the target gene expression and to correct sample-to-sample variation; similar results were obtained with *ribosomal protein S3* as the reference gene. The standard error is represented by the error bar, and the different letters (a, b, c) above each bar denote significant differences (p<0.05).

**Figure 11:**
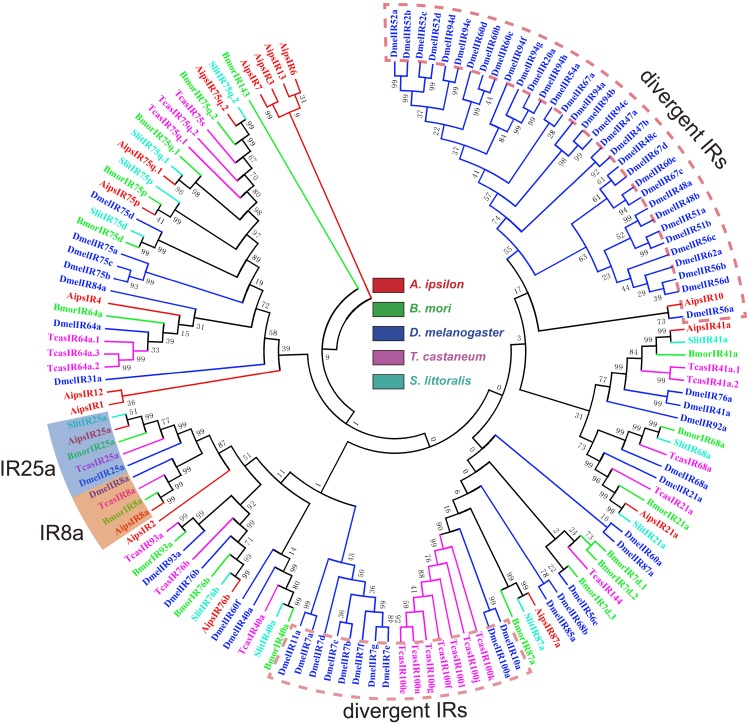
Neighbor-joining tree of candidate ionotropic receptor proteins from different insect species. The protein names and sequences of IRs that were used in this analysis are listed in Table S6.

Insect chemosensory ionotropic receptors (IRs) belong to an ancient chemosensory receptor family, that was first discovered in *D. melanogaster* and are expressed in sensory neurons that respond to different odorants but that do not express either ORs or gustatory receptors (GRs) [40]. The misexpression of *D. melanogaster* IRs conferred ectopic odorant responsiveness [40]. At present, 66 IRs in *D. melanogaster* [41], 12 IRs in the noctuid *S. littoralis* [42], 15 IRs in *C. pomonella* [45] and 12 IRs in *H. armigera* [46] have been identified. In the present study, we have identified 24 IRs, including two highly conserved coreceptors, IR8a and IR25a, from the *A. ipsilon* antennal transcriptomes (Table 5). Five of the IR genes, including coreceptors IR8a and IR25a, had intact ORFs. Eighteen of these 24 IRs showed high amino acid identity (52%–90%) with three Lepidoptera insects, *C. pomonella*, *S. littoralis* and *B. mori*. Similar to the ORs, the RPKM value analysis revealed that all the 24 IRs showed a relative low expression level (RPKM value ranged from 0 to 69) compared with the OBPs and CSPs. The antennae-enriched IRs may play important roles in odorant detection; 15 *D. melanogaster* IRs [40], 10 *H. armigera* IRs [46] and 7 *S. littoralis* IRs [42] were expressed exclusively in the antennae. Our RT-PCR and RT-qPCR results indicated that 14 *A. ipsilon* IRs (*IR8a*, *IR25a*, *IR21a*, *IR41a*, *IR75q.1*, *IR75q.2*, *IR76b*, *IR87a*, *IR1*, *IR3*, *IR4*, *IR8*, *IR12* and *IR13*) are highly expressed in the antennae; in particular, one IR *IR12* was specifically expressed in the male antennae (Figure 7 and Figure 10), which suggested that this IR may be devoted to the response to the female sex pheromones. IRs from different insect species are extremely divergent; however, the two coreceptors IR8a and IR25a are highly conserved among different insect species (Figure 11).

**Table 5:** List of IR, GR and SNMP genes in *A. ipsilon* antennae.

Unigene	Gene	Length (bp)	ORF (bp)	BLASTx annotation	Score	*E*-value	% Identify	RPKM value	
								Male	Female
**Ionotropic receptors (IRs)**									
Unigene_13668	IR8a	3301	2442	gb|AFC91764.1| putative ionotropic receptor IR8a, partial [Cydia pomonella]	1047	0.0	82%	68	28
Unigene_1879	IR25a	2960	2772	gb|AFC91757.1| putative ionotropic receptor IR25a [Cydia pomonella]	1562	0.0	87%	60	35
Unigene_13062	IR21a	2175	---	gb|ADR64678.1| chemosensory ionotropic receptor IR21a [Spodoptera littoralis]	1204	0.0	83%	24	19
Unigene_149	IR41a	975	---	gb|ADR64681.1| chemosensory ionotropic receptor IR41a [Spodoptera littoralis]	608	0.0	73%	12	17
Unigene_368	IR75q.1	1464	---	gb|ADR64686.1| chemosensory ionotropic receptor IR75q.1 [Spodoptera littoralis]	524	2e-173	58%	15	25
Unigene_302	IR75q.2	2168	1881	gb|AFC91752.1| putative ionotropic receptor IR75q2 [Cydia pomonella]	811	0.0	69%	30	23
Unigene_11960	IR75p	492	---	gb|ADR64684.1| chemosensory ionotropic receptor IR75p [Spodoptera littoralis]	208	2e-60	65%	40	14
Unigene_21404	IR76b	2236	1629	gb|ADR64687.1| chemosensory ionotropic receptor IR76b [Spodoptera littoralis]	917	0.0	85%	37	18
Unigene_31214	IR87a	534	---	gb|ADR64689.1| chemosensory ionotropic receptor IR87a [Spodoptera littoralis]	330	3e-109	90%	21	9
Unigene_16111	IR93a	228	---	gb|AFC91753.1| putative ionotropic receptor IR93a, partial [Cydia pomonella]	117	3e-28	69%	15	0
Unigene_14129	IR1	897	---	gb|ADR64688.1| putative chemosensory ionotropic receptor IR1 [Spodoptera littoralis]	333	9e-106	74%	10	5
Unigene_1452	IR2	1651	1452	ref|XP_001655464.1| glutamate receptor [Aedes aegypti]	477	1e-155	55%	3	0
Unigene_11336	IR3	876	---	gb|EHJ72198.1| ionotropic glutamate receptor-invertebrate [Danaus plexippus]	187	6e-51	39%	19	4
Unigene_13871	IR4	1317	---	gb|AFC91763.1| putative ionotropic receptor IR4, partial [Cydia pomonella]	166	5e-45	52%	15	7
Unigene_18619	IR5	342	---	ref|XP_002431269.1| glutamate receptor [Pediculus humanus corporis]	74.7	3e-13	41%	5	11
Unigene_11730	IR6	972	---	gb|ABD36124.1| glutamate receptor Gr1 [Bombyx mori]	329	2e-100	80%	67	69
Unigene_23096	IR7	615	---	gb|ADR64688.1| putative chemosensory ionotropic receptor IR1 [Spodoptera littoralis]	332	7e-108	76%	9	18
Unigene_22453	IR8	330	---	gb|ADR64681.1| chemosensory ionotropic receptor IR41a [Spodoptera littoralis]	156	8e-43	68%	11	0
Unigene_19516	IR9	307	---	ref|XP_002431270.1| glutamate receptor [Pediculus humanus corporis]	201	7e-59	89%	6	12
Unigene_8307	IR10	714	---	gb|EHJ70235.1| ionotropic glutamate receptor-invertebrate [Danaus plexippus]	99	4e-22	46%	3	2
Unigene_19025	IR11	255	---	gb|ADR64683.1| chemosensory ionotropic receptor IR75d [Spodoptera littoralis]	152	8e-42	86%	15	0
Unigene_11460	IR12	723	---	ref|XP_001845244.1| ionotropic glutamate receptor [Culex quinquefasciatus]	59.7	5e-07	31%	5	8
Unigene_945	IR13	1134	---	gb|ADR64689.1| chemosensory ionotropic receptor IR87a [Spodoptera littoralis]	172	6e-78	84%	13	7
Unigene_17908	IR14	287	---	gb|ADR64684.1| chemosensory ionotropic receptor IR75p [Spodoptera littoralis]	138	7e-36	56%	9	4
**Sensory neuron membrane proteins (SNMPs)**									
Unigene_4432	SNMP1	2756	1569	emb|CAB65739.1| sensory neuron membrane protein-1 [Heliothis virescens]	947	0.0	85%	136	165
Unigene_34376	SNMP2	1869	1563	emb|CAP19028.1| sensory neuron membrane protein-2 [Heliothis virescens]	884	0.0	84%	354	481
**Gustatory receptors (GRs)**									
Unigene_10727	GR63	720	---	tpg|DAA06395.1| TPA_inf: gustatory receptor 63 [Bombyx mori]	131	6e-31	46%	11	6

“---” represent that gene is partial and has not intact ORF. The nucleotide sequences of all 24 IR, 2 SNMP and 1 GR genes are listed in Table S2.

### Candidate sensory neuron membrane proteins and gustatory receptors in the *A. ipsilon* antennae

Insect SNMPs are two trans-membrane domain-containing proteins that are suggested to play significant roles in insect chemoreception [74]–[76]. Two SNMP subfamilies, SNMP1 and SNMP2, were identified in insects; however, these subfamilies showed different expression profiles in the antennae sensilla: SNMP1 proteins are detected in pheromone-sensitive olfactory receptor neurons (ORNs) [77]–[79]; however, the SNMP2 proteins are expressed in the supporting cells [78], [79]. In the present study, we have identified two SNMP genes, *SNMP1* and *SNMP2*, in the *A. ipsilon* antennal transcriptomes (Table 5). Both have intact ORFs with lengths of 1569 bp and 1563 bp for *SNMP1* and *SNMP2*, respectively, in agreement with our previous analyses [79]. The RT-PCR and RT-qPCR results revealed that both *SNMP1* and *SNMP2* were primarily expressed in the antennae of both sexes (Figure 7 and Figure 10). Furthermore, one gustatory receptor (*AipsGR63*) was identified in the *A. ipsilon* antennal transcriptomes (Table 5); AipsGR63 showed 46% amino acid identity with the *B. mori* gustatory receptor 63. The RT-PCR and RT-qPCR analyses showed that *AipsGR63* was expressed in both the antennae and body part (Figure 7 and Figure 10).

## Conclusions

Olfaction is an important sensory modality in insect. In present study we have successfully identified and annotated several groups of olfactory genes in the antennae of the noctuid moth *A. ipsilon*. The expression profile analysis revealed that 22 OBPs, 3 CSPs, 35 ORs, 14 IRs and the 2 SNMPs are uniquely or primarily expressed in the male and female antennae. These antennae-enriched OBPs, CSPs, ORs, IRs and SNMPs may play important physiological function in the pheromone and general odorant detection; thus, these genes could be meaningful targets for the study their biological functions, both *in vivo* and *in vitro*. An important direction of our future research will be the functional study of these olfactory genes.

## Materials and Methods

### Ethics statement

The black cutworm moth *Agrotis ipsilon* is common agricultural insect pests and are not included in the “List of Endangered and Protected Animals in China”. All operations were performed according to ethical guidelines in order to minimize pain and discomfort to the insects.

### Insect rearing and tissue collection

The *A. ipsilon* colony was established in our laboratory in 2006. The larvae were reared with an artificial diet that was composed of wheat germ, casein and sucrose as the main components [63], [64], [79]. The laboratory colony was kept at 24°C with 75% relative humidity and a 16∶8 light:dark cycle. Pupae were sexed and maintained separately in hyaline plastic cups before emergence. Adult moths were given a 20% honey solution after emergence. Antennae were excised from 3-day-old male and female moths and immediately frozen and stored in liquid nitrogen until use.

### RNA extraction and cDNA library construction

400 antennae from each sex were polled for total RNA extraction using TRIzol reagent using TRIzol reagent (Invitrogen, Carlsbad, CA, USA) following the manufacturer’s instructions. The quantity of RNA samples was determined using a NanoDrop spectrophotometer (Thermo Scientific, Wilmington, DE, USA) and 1.1% agarose electrophoresis. Approximately 500 ng messenger RNA was further purified from 50 µg total RNA using a PolyATtract mRNA Isolation System III (Promega, Madison, WI, USA). The mRNAs were then sheared into approximately 800 nucleotides via RNA Fragmentation Solution (Autolab, Beijing, China) at 70°C for 30 sec, then cleaned and condensed using an RNeasy MinElute Cleanup Kit (Qiagen, Valencia, CA, USA). The first-strand cDNA was synthesized using N6 random primers and MMLV reverse transcriptase (TaKaRa, Dalian, China). Then, the second strand cDNAs were synthesized using secondary strand cDNA synthesis enzyme mixtures (Autolab, Beijing, China). The cDNAs with the desired length were purified using a QIAquick PCR Purification Kit (Qiagen, Valencia, CA, USA) and eluted with 10 µl elution buffer. After blunted and appended with a poly-A tail at the 3′ end according to Roche’s Rapid Library Preparing protocols (Roche, USA), the purified cDNAs were linked to GS-FLX Sequencing Adaptors (Roche, USA). Finally, the cDNAs that were shorter than 500 bp were removed using AMPure Beads according to the manufacture’s instructions (Beckman, USA) before the preparation of the cDNA library for next generation sequencing.

### 454 sequencing

Pyrosequencing of the cDNA library was performed by the Beijing Autolab Biotechnology Company using a 454 GS-FLX sequencer (Roche, IN, USA) according to the manufacturer’s instructions. All sequencing reads were deposited into the Short Read Archive (SRA) of the National Center for Biotechnology Information (NCBI), and can be accessed under the accession numbers SRR838973 and SRR838974 for the male and female antennal transcriptomes, respectively.

### Sequence analysis and assembly

Base calling of the raw 454 reads in SFF files were performed using the python script sff_extract.py that was developed by COMAV (http://bioinf.comav.upv.es). All the raw reads were then processed to remove low quality and adaptor sequences using the programs TagDust [80], LUCY [81] and SeqClean [82] with default parameters. The resulting sequences were then screened against the NCBI UniVec database (http://www.ncbi.nlm.nih.gov/VecScreen/UniVec.html) to remove possible vector sequence contamination. The cleaned reads that were shorter than 60 bases were discarded based on the assumption that these reads might represent sequencing artifacts [83].

Two steps were taken to assemble the clean reads. First, the sequence assembler MIRA3 [84] was used with the assembly settings of a minimum sequence overlap of 30 bp and a minimum percentage overlap identity of 80%. Then, CAP3 was used with the assembly parameters of an overlap length cutoff >30 and an overlap percent identity cutoff >90% [85]. The resulting contigs and singletons that were more than 100 bases were retained as unigenes and annotated as described below.

### Homology searches and functional classification

Following the assembly, homology searches of all unigenes were performed using the BLASTx and BLASTn programs against the GenBank non-redundant protein (nr) and nucleotide sequence (nt) databases at NCBI [86]. Matches with an E-value that was less than 1.0E-5 were considered significant [54]. Gene names were assigned to each unigene based on the best BLASTx hit with the highest score value.

Gene Ontology terms were assigned by the tool Blast2GO [87] through the BLASTx program with an E-value less than 1.0E-5. Then, the WEGO [88] software was used for the assignment of each GO ID to the related ontology entries. The longest open reading frame (ORF) of each unigene was determined by an ORF finder tool (http://www.ncbi.nlm.nih.gov/gorf/gorf.html).

### Identification of *A. ipsilon* chemosensory genes

The tBLASTn program was performed, with available sequences of OBP, CSP, OR, GR, IR and SNMP proteins from Lepidoptera species as “query” to identify candidate unigenes encoding putative OBPs, CSPs, ORs, GRs, IRs and SNMPs in the *A. ipsilon*. All candidate OBPs, CSPs, ORs, GRs, IRs and SNMPs were manually checked by the BLASTx program at the National Center for Biotechnology Information (NCBI). The nucleotide sequences of all chemosensory genes that were identified from the *A. ipsilon* antennal transcriptomes are listed in Table S2.

### Comparative analysis of chemosensory genes in the *A. ipsilon* male and female antennae

To compare the differential expression of chemosensory genes in the *A. ipsilon* male and female antennal transcriptomes, the read number for each chemosensory gene between male and female antennae was converted to RPKM (Reads Per Kilobase per Million mapped reads) [89], using the formula: RPKM (A) = (1,000,000×C×1,000)/(N×L), where RPKM (A) is the expression of chemosensory gene A, C is the number of reads that are uniquely aligned to chemosensory gene A, N is the total number of reads that are uniquely aligned to all unigenes, and L is the number of bases in chemosensory gene A. The FDR (false discovery rate) was used to determine the threshold of the P-value for multiple testing. FDR <0.001 and absolute values of the log_2_ratio >1 were used as the threshold to determine significant differences in gene expression. The RPKM method eliminates the influence of gene length and sequencing depth on the calculation of gene expression. Thus, the calculated gene expression can be directly used to compare gene expression between samples.

### Sequence and phylogenetic analysis

The putative N-terminal signal peptides and the most likely cleavage site were predicted using the SignalP V3.0 program [90] (http://www.cbs.dtu.dk/services/SignalP/). Sequence alignments were performed using the program ClustalX 2.1 [91] with default gap penalty parameters of gap opening 10 and extension 0.2, and were edited using the GeneDoc 2.7.0 software. A neighbor-joining tree [92] was constructed using the program MEGA 5.0 [93] with a p-distance model and a pairwise deletion of gaps. The bootstrap support of tree branches was assessed by re-sampling amino acid positions 1000 times.

### RT-PCR and RT-qPCR analysis

Two biological samples each with 150 male antennae, 150 female antennae and two moth body part (mixture of heads, thoraxes, abdomens, legs, wings) were used for RNA extraction using TRIzol reagent. Before transcription, total RNA was treated with RQ1 RNase-Free DNase (Promega, Madison, USA) to remove residual genomic DNA. cDNAs from male antennae, female antennae and the body part were synthesized using a GoScript Reverse Transcription System (Promega, Madison, USA). An equal amount of cDNA (200 ng) was used as RT-PCR and RT-qPCR templates. Specific primer pairs that were used for RT-PCR were designed with the program Primer 3 (http://frodo.wi.mit.edu/) (see Table S3). The *β-actin* (GenBank Acc. JQ822245) of *A. ipsilon* was used as the control gene to test the integrity of the cDNAs. The PCR was performed under following conditions: 95°C for 2 min, followed by 25–35 cycles (depending on the expression level of each gene) of 95°C for 30 sec, 56°C for 30 sec, 72°C for 1 min, and a final extension for 10 min at 72°C. PCR products were analyzed on 1.2% agarose gel and visualized after staining with ethidium bromide. To reach reproducibility, each sample was performed at least six times with two biological samples.

RT-qPCR analysis was conducted using an ABI 7500 Real-Time PCR System (Applied Biosystems, Carlsbad, CA). The primers that were used for RT-qPCR were designed using the program Beacon Designer 7.90 (PREMIER Biosoft International) (see Table S4). Two reference genes, *β-actin* (GenBank Acc. JQ822245) and *ribosomal protein S3* (GenBank Acc. JQ822246) were used for normalizing the target gene expression and for correcting for sample-to-sample variation. Each RT-qPCR reaction was conducted in a 25 µl reaction mixture containing 12.5 µl of SuperReal PreMix Plus (TianGen, Beijing, China), 0.75 µl of each primer (10 µM), 0.5 µl of Rox Reference Dye, 1 µl of sample cDNA, and 9.5 µl of sterilized H_2_O. The RT-qPCR cycling parameters were as follows: 95°C for 15 min, followed by 40 cycles of 95°C for 10 sec and 60°C for 32 sec. Then, the PCR products were heated to 95°C for 15 sec, cooled to 60°C for 1 min, heated to 95°C for 30 sec and cooled to 60°C for 15 sec to measure the dissociation curves. Negative controls without either template or transcriptase were included in each experiment. To check reproducibility, each RT-qPCR reaction for each sample was performed in three technical replicates and two biological replicates. The comparative 2^−ΔΔCT^ method [94] was used to calculate the relative quantification between tissues. The comparative analyses of each target gene among various tissues were determined using a one-way nested analysis of variance (ANOVA), followed by Tukey’s honestly significance difference (HSD) test using the software SPSS Statistics 18.0 (SPSS Inc., Chicago, IL, USA). When applicable, values were presented as the mean±SE.

## Supporting Information

Table S1 The 500 most highly abundant unigenes in the *A. ipsilon* antennal transcriptome.
(XLSX)

Table S2 The nucleotide sequences of 33 OBPs, 12 CSPs, 42 ORs, 24 IRs, 2 SNMPs and 1 GRs identified in present study.
(DOCX)

Table S3 Primers used for RT-PCR analysis of olfactory genes of the *A. ipsilon* moth.
(DOCX)

Table S4 Primers used for RT-qPCR analysis of olfactory genes of the *A. ipsilon* moth.
(DOCX)

Table S5 Protein names and sequences of ORs used in Figure 9.
(TXT)

Table S6 Protein names and sequences of IRs used in Figure 11.
(TXT)
