# Supplementary material for: Molecular Characterization and Differential Expression of Olfactory Genes in the Antennae of the Black Cutworm Moth Agrotis ipsilon
Source: PLoS One. 2014 Aug 1;9(8):e103420. doi: 10.1371/journal.pone.0103420 (PMC4118888; doi:10.1371/journal.pone.0103420)
Supplement: Table S2 — The nucleotide sequences of 33 OBPs, 12 CSPs, 42 ORs, 24 IRs, 2 SNMPs and 1 GRs identified in present study. (DOCX) [file pone.0103420.s002.docx]

**Table S2.** The nucleotide sequences of 33 OBPs, 12 CSPs, 42 ORs, 24 IRs, 2 SNMPs and 1 GRs identified in present study.

>AipsPBP1 ORF 513 bp

ATGGCACCGCATCCATCTGTCACCATGTATGTTCGTTTGGCGCTGGTGATCATCGCGGGACTTTTCATCACGGTAGAGTGCTCGCAGGAGATCATCAAGAATCTGTCTTTGCAATTCGCTAAACCTTTGGAGGACTGCAAAAAGGAGATGGACCTCTCTGACACTGTGATCACAGACTTCTACAACTTCTGGAAGGAAGGCTATGAGTTCACGAACAGACAGTTCGGTTGTGCCATCCTGTGCCTCTCATCGAAGCTGGAACTGCTAGATCAGGATCTGAAGTTACATCACGGCAAGGCGCAGGAGTTCGCGAAGAAACATGGCGCTGACGAGGCGATGGCGAAGCAGCTAGTAGACATGATCCACAGCTGCACACAGTCGACCCCTGACGTGGCTGATGACCCCTGCATGAAGACCCTCAACGTGGCCAAGTGCTTCGTGGCGAAGATCCACGACCTCAAGTGGGCGCCGAGCATGGACCTTATCATGGGGGAAGTTTTGGCTGAAGTCTAG

>AipsPBP2 ORF 498 bp

ATGGCGGCCTCAAGATGGTGTATCGCGTGCCTCGTCTGCGTCCTGTTTGCTGCAAGGTCGGTGATGACGTCGCAGGAGGTCGTCGCCAGCTTCAGCAAAGGGTTCACCAATGTTGTGGAGCACTGTAAAGCTGAGGTGAACGCGGGAGAACACATCATGCAAGACATATACAACTTCTGGCGCGAGGAGTACCAGCTGGTGAACCGCGACCTGGGCTGCATGGTGCTGTGCATGGCCAACAAGCTGGGGCTCATAGGAGAGGACCAGAAGATGCACCATGCGAAGGCTGAGGAGTTCGCTAAGAGTCACGGAGCCGACGAAGCAGTAGCCAAGCAGCTGGTAGCCATTCTCTACGAATGCGAGACCAAGCACGCGGCCGTCGAGGACGAGTGCGGCATGGCGCTGGAGATCGCCAAGTGCTTCCGCACCAAGATGCACGAGCTTAAGTGGGCGCCCAGCATGGAAGTAGCCATGGAGGAGATCATGACGGCCGTTTAG

>AipsPBP3 ORF 495 bp

ATGGGAACGTACAACGTCTTTTTCGCATTTGTGTTGATGGCAGCAGGAGTGAGGGAGATAGAACCCTCGAAGGATGCAATGAAGTACATCACTTCAGGGTTCGTTAAGGTTTTGGAGGAGTGTAAGCAAGAGCTTAACATGAACGATCGGATCATAGCGGACCTGTTCCACTACTGGAAGTTGGACTACACGCTGCTGAACCGCGACACCGGCTGCGCCATCATCTGCATGAGCAAGAAGCTGGACCTGCTGGACGACACCGGCAGAATGCATCATGGAAACGCTCAGGAGTTCGCGCTCAAACATGGCGCTGGAGAGGAAGTAGCGTCAAAGATAGTAACCATCATCCACGATTGTGAGAAGAAGTTCGAGAGGGACGATGACGAGTGTCTGCGAGTGCTGGAGGTGGCCAAGTGCTTCCGCACCGGCATCCACGACCTGGACTGGCAGCCCAAGGTGGAAGTCATCGTCTCCGAGGTCTTCACCGACATGTAG

>AipsGOBP1 ORF 495 bp

ATGACGCAGCCGGGACAGGTGCTGGTGCTGGTGCTGCTGGCGGCGGCCGCGCTCGCCGACGTCAACGTCATGAAGGACGTCACGCTCGGCTTCGGGCAGGCACTGGACAAGTGTCGGCAGGAGAGTGACCTCACGGAGGAGAAAATGGAGGAGTTCTTCCACTTCTGGCGCGATGACTTCAAGTTCGAGCACCGCGAGCTGGGCTGTGCCATCCAGTGCATGAGCCGCCACTTCAACCTGCTCACCGACTCCAGCCGCATGCACCACGTCAACACCGAGGAGTTCATTCAGTCCTTCCCCAACGGCGAGGTGCTGGCGCGGCAGATGGTGGCGTTGATCCACGGCTGCGAGAAGCAGTTCGACCACGAGGACGACCACTGCTGGCGCATCCTGCACGTGGCCGAGTGCTTCAAGCACGCGTGCGTGGCGCACGGCGTGGCGCCCTCCATGGAGATGATGATGACCGAGTTCATCATGGAGGCGGAGGCGCGGTGA

>AipsGOBP2 ORF 489 bp

ATGACGTTGAGGTGTTGTTTGTTGCTGGTTGTCGTGGCTGCCGTCACCAGGTCCGTGGTGGGCACCGCGGAGGTGATGAGCCACGTCACCGCACATTTTGGTAAAGCTCTTGAAGAGTGCAGGGATGAGTCAGGGCTCTCAGCGGAGGTGCTGGAGGAGTTCCAGCACTTCTGGCGCGAGGACTTCGAGGTCGTGCACCGCGAGCTGGGCTGCGCCATCATCTGTATGTCCAACAAGTTCTCGCTGCTGCAGGACGACAGTCGCATGCATCACGTCAACATGCACGACTACGTCAAGGGATTCCCTAATGGTGAGGTTCTCTCCGGAAAGCTAGTGGAACTGATCCACAACTGCGAGAAGCAATATGACACGTTGACGGACGACTGCGACCGCGTCGTCAAGGTAGCTGCATGCTTCAAGGTGGACGCGAAGGCGGCTGGCATCGCGCCCGAGGTGGCCATGATCGAGGCTGTCATGGAGAAGTACTGA

>AipsOBP1 ORF 543 bp

ATGGACATCTCAAAAAGACGAAGCAAGAATGCATTTAGAAGATTGCTGGTTAATACCTGGTTACGACTTGTGCAGATCTTCACCTGTCTCAGCGCTCCGCCAGTGGTTAGTGCTGACGTGACGTCCAAATGCCAAGGATCCAAGTACGAAAATGAGTGCGACAAACTGACCTGTGTGTTCCGCAAGGCCAAATGGCTAGATGGTAACGCGGTAGACAAGGCGAAGCTGATCACGTACTTCGAACAGTTCGAGAAGGATCACCCGGAGTGGGCACCAGCCATGCAGAATGTGAAGACTTCCTGCCTGGGGGCTGAGCTGAAGACACAGGGAGTCTTCCTCAACTGCCCAGCTTATGATGTCATGCATTGTGTGTTGGGTAGCTTCATTAAGCACGCGACTCCAACTCAATGGTCCACCTCGGCGTCATGCTCATACCCCCGCGCATACGCAGCTGCCTGTCCCATCTGCCCAGAAGATTGTTTCAGCGCACAAGTGCCCTTCGGATCCTGTAACGCCTGTTATTTGCCGCCGAGGACACCCTAA

>AipsOBP2 ORF 447 bp

ATGTCTAAGTTCACTTGTTTGGTTTTGTGTGTAGTGGCTGCGAGTATTAGCAGGGTTCATGCAGATGATGATGCAAATAAAGCAGCTTTCCGTGAAGCTTTCAAACCCATCTTAGACGAGTGCTCAAAGGAACATGGAGTTAGCAACGATGACATTGATGCTGCAAAGAAAGCAGGCAGCGCTGACGCTATCAAACCTTGCTTCTTCGGATGCATCTACAAGAAAGCCGAAGTTTTCAATGCCAAGGGAGAATACGATGTTGACAGCGCTCTGAGCAAGCTGAAGAAGTTTGTGCCCGATGAAGCTAAATTCGCTAAATACGCTGAAATTGGAAAGAAATGCGCATCAGTAAATGAGAAGCCCGTAACCGATGGCGACGCTGGATGCGAGAGGGGTGCGATGCTGACTGCTTGCTTCTTGGAAAACAGGGCCGAGATGCTCATTTAA

>AipsOBP3 ORF 588 bp

ATGATCAGATCCTGTCGTTGTTTAGTGTTTGCTGCTGTGTTTCAGGTTGTATTAGGCCAGGGCCTAACCGGCACAGACTCGGGACCACCAGGATTTCAAAGGCCACAATCATATGTACCAAAGCATTGTTTCGCTCCACCTCCTGGGGTGGACCTCCACACATGTTGTCCAATACCCCAACTATTTCCTGATGAAGACATGGAAAGTTGTGGAATTCAGAAATTGACAAAGGAACAATACGAAAACCCATCACCAGCTAGAATTCCCTGCCAAGAAAGCATTTGTTTGCTGCGCAATGCTAATTTACTCAAGCAAAACAATAGTATAGACTATGAGAAAATGGGAGATTTTGTAGATAACTGGGCAAAGATGGACCCTGATTTTACCATACCTATAACTAATGCCAAGAAGGTATGTTTAATAGAGGGAGGACCACCAGCTCCACCAGTTTGTGAACCAGACAGGATCTTCACTTGTTTGACTTCTTATGTATTATGGAACTGTAAGCTAAGATTGGACAGTGGAGAGGGCTGTAAGATACTAAAGGAGCATATGGATGGATGCAGACCATTTCTGGCCGGTCCATGA

>AipsOBP4 ORF 453 bp

ATGTTTGGATACCAGTTCCTGTCGTTTGCGGCTGCTCTGATCTGTTTTGGGAGCAGTTATGCTCTAACTTCAGAGGAAGAGGCTAATATAAAAGAAGCTTTTCATCCATTCATAATGAAATGTGCAGAGGAATATGGTATAACTGAGGAACAATTTGAAGAAGCGAAGGAGAAACACAGCGCTGAGGGCATTGACCCTTGTTTCATGAGCTGTTTTATGAAGGAATCTGGATTTTTTGACAGCGCGGGTAAGTTTGACGCGGACAAGACCAAGGAATTTGTTGACGCCCATTTGACGAGTGAGCGAGCTATTACGTTCATGGAAGCAGTTGGTTCAGAATGTGCTAAAGTGAACGATGAAGAAGTGACTGACGGTGATAAGGGATGTGATCGAGCAAAGTTGATGTGGGGCTGTATACAAGATCTTAAAGAAAAAATGGAGGGTAGTGAATGA

>AipsOBP5 ORF414 bp

ATGAAATACTTCGTGTTATTTGTTGCCCTGGTAGCTGGGATTCACGCTAACGTGACTCTCCCTCCGGAACAGAGCGAGAAAGCTCTAAAGACAGCATCGGAATGCATCAAAGAAACTGGAGTCTCGAAAGAGGTATTAGCCGAAGCGAAGAAAGGTCACATTGCTGATGACGAGGGTCTAAAGAAATTCACTCTATGCTTCTTCAAAAAAGCAGGAATCGTCGACAATGATGGCAAGTTGAACTTGGAGACAGCACTCGCGAAGTTACCTCCAGGAGTCGACAAAGCTGAAGCAAAGAAGGTTCTGGAAGGATGCCAGGCGAAATCTGGAAAGACACCACAGGATACTGCGTTTGAAATCTACAAATGTTACCACGCTGGTGCTAAAACCCATATTGCTTTAGCAGGAATTTAA

>AipsOBP6 partial 378 bp PG-enriched expressed

CAAAGAGAAAACAAAGGCGCTTCTTTGAAACCTTTATCAGTATGCTGTGATATACCAGAGCTGGGTGATCCAAAACATCTGGCAAAGTGTTCCAACCCTAAACTACCAGGACCGTGCAACGACGTCCAATGCGTGTTCGAAGAATCCGGTTTCCTGACAGACAAGAATACTCTGAACAAGGAAGCTTACAGAAATCACTTGAAGCAATGGGAAGAGAACAATAAAGGGTGGACTGTCGCTGTGGACAAGGCTATCAAGGAGTGTGTGGACAACGACCCCAGGCAACATCTGGACATCCCTTGCAAGGCGTATGACGTCTTCACTTGCACTGGAATTGCTATGTTGAAGAAATGTCCAGATTCAGCATGGAAATGTTAG

>AipsOBP7 ORF 438 bp ATGTCTAAGTTTACTTGTGTATTGTGTGTCGTGGCTTTGAGCTTAAGCAGCGTTTATGTTACGAGAGCTCATAAACCCAATCTTCGCGATGCCTGGAGGTCGGAATTGGATGAATGTGCAAAGGAATATCCAGTCACGAACGATGAAATAGACACGGCTGTGAGATCAGGCGATTCTTCGAATTTAAATCCTTGTTTCAATTTTTGTGTCTTTAATAAAACAGGATTCTTTACTGAAAATGGTGAATACGATCTGAAAAACGGTCTCATCAAACTTAGGAAGGCTATTCGAGATGATGAAGAATATACTAAATTTGAAGAAGTTGCTACAGAGTGCACAGAGGACAAAAACACATCATGCGATGAAAAAGCTAAATGTGACAGCGCCAACCGTTTGTCTCTTTGCTTTTTGCGATTCAAAGACAAGGTCCGGATATAG

>AipsOBP8 ORF 507 bp

ATGTATTTGCGGTCTACAAATGGAGGAGTTCGATCCTTCCCGTTAGGTGAATCTGCCTACACAACTAAGATCGTTGAAATCTGTAGCAAGGAAACTGGACTCAAAAAACAAGTGCCTCCCGAGGAGAAAGAAATAAAATTCAGTCAAAGAAAAGGCCTCAGGGAATTCAATGATTGCTACTTAGCAAAAACCGGTGTCACCACTAGTGATGGGAAACTAAATATCGATGAGGCTTTAGAAAAACTCCCGCCAGGTTTTGCTAAGCCGTTCGTAGAACACTGCCAAGCAAATATTATTTTAGGGTACATAGAAGAGAACGTCAATGATTTTTCTACATGTTTCCATCAAGAAGTACAGAATCATCTTTTAAGTTTTTACGGGTTTGAAAATTATTGGGTAATGCTTGTTCTTGGGACTTCTTTTGATAAGACTCGTTTTACAACTTTATTTTTTGATAAACACTTTGATTTCTGGTTAGCTGAACGGGCTGGATTCGTTAATTTGTAG

>AipsOBP9 partial 423 bp

GTATTTATTTGTGGTGTGTTGTCTTTAAACGTTAAGGCATCATCCTTAGACGAACTGAAGATGAAATACGTGGAGATGATAATAGAATGTTCAGACACATATCCGATAACAGCGGCTGATACGCTACAGCTCAAGACCAAGACAATGCCTGATAACGAGTCAATCAGATGCCTTTTTGCTTGCGTTTATAAGAAGGCTGGAATGATGAACGAGCAAGGAGAATTATCAGTTGAAGGTGTGAATGAGATGACCCGACGATACTTATCTGATGACCCTGATAAAATTAAGAAGAGTGAACAATTCACGGAGGCATGTAAAAGCGTAAACGATGTCCCTGTCAGCGATGGAACAAGAGGCTGTGATAGAGCTGCCCTGATCTTCAAATGCACAGTTGAAAAGTCACCTGATTTTGACCTTCTGTGA

>AipsOBP10 partial 234 bp

TGTTATCGCCTTAAGACACAATATTCTGTGTTCCATTTCAGGAAAATAAAACGTTATGTTCTTAGATTTTCAAACTACGTCTTTTTCATTTCAGACCCATTTCCTGGTTTTAAAAAATGTAAGAGCGCGGATGAGGAGTGTGTGCTAGAAAATGCTAAAGCTGGAGTAGTGCCCTTTGTTAATGGCATACCAGAGTTTGGTGTAAACAAATTGGATCCAGATTTTTTAAGGTAG

>AipsOBP11 ORF 726 bp

ATGACCTATAAAGTTTTTATTTTAGTTTTTCTAACGTACGTCTCTTTGGCGACGTCTGCTTTGGCACCTTTCATCACAAAATGCAAATGGGACGACTCGAAGTGTATCAAAGAGTCAGCGCAGAAGGTGATACCACTATTCGCTGACGGTATTCCAGATCTACATGTGGAGAAGCACGACCCCCTTTTGATTAAAAGGGTAGACGCCAGTTCTCCAAATTTGAAACTTATCGTCACCGACATCGAAGTTAAAGGACTCAAGAATTGTGAGGCTAAGAAAATAACGAGAGATCTCAAAGCTATGAAGCTGAGCGTCAAATTTCTATGTGCTGTAGACTTCAAGGGAGTTTATGACATGAAAGGACAGCTCTTCGTTTTACCTATTGAAGGCAATGGAGATCTCACAGCTCATGTCCCTAAGATCCAGCTAAACGCAGAAGTAGATATGGTCGACAAGACAGGAAAAGACGGAAAGAAGCATTGGGGAGTTAAATCGTGGAGGCACAGTTTTGAATTGAAGGAAAAATCGAATGTGAAATTCGAAAACTTATTCCCCGACAACGAATTCTTGCGCAAAACAACCGAAGAACTGATTGCTAGCAACGGCAATGATGTTATCGTAGAAGTAGGACCTGAAATCATAAAAGCGGTAACAGCTAAGGTCATCGAATCCATAAAGAAACTTTTCGATGAAGTTCCAGTGGAAGAACTTGCAATTGATGAATGA

>AipsOBP12 ORF 441 bp

ATGTACTCCGGTACCATATTTCTCTTCTCGTTTATTTTGCTGATTGTGTCAAACGTTACTTTCGTCAGTTCGCAAATGACAAGGGAACAAGTCAAAAACTCTGGAAAATTGGTTAAAAAGACGTGTTCAGCGAAAAATGATCTTACTGAAGATGAAGTGAAAGATGTAGACAAAGGAAAATTCATAGAGGAGAAAAAATTCATGTGTTACGTGGCATGTGTGTACAAAATGGGACAGGCCGTAAAAGGCAACTCTTTAAACCATGATATGATGATCAGACAAGTGGATATGTTGTTCCCAGCAGACATGAAGGCTCCTGTTAAAGCAGCAATAGAACATTGTAGGCCTGTTGCTAAGAAATACAAGGACATCTGTGAGGCATCTTATTGGACAGCAAAGTGCGTTTATGAATTTGATCCACCAAATTTCATGTTCCCTTAA

>AipsOBP13 ORF 420 bp

ATGGTTCTCATTTATATTGTGAAGTTTTTGATATTGGTTGCAATGTGTGAAGCTATGACAATGAAACAAATCAGGAATACTGGCAAAATGATGAGGAAATCGTGCCAACCAAAGAATAATGTTGAAGATGAAAAAATCGATCCCATTGCGGAAGGAATCTTCATTGACGAGCCAGAAGTAAAGTGTTACATGGCTTGCATTATGAAAATGGCTAACACGCTTAAAAATGGGAAATTAAATTTCGATGCCGCCTTGAAACAAGCTGATTTATTGCTACCTGATGATATAAAGGAGCCAGCTAAGGAAGCTATCATAGCTTGTAAGAAAGCTGCGGAAGGCCACAAGGATATTTGTGACGTTTCATTTCATGTCACAAAATGCATCTACAATCAGAACCCGGGCATATTTTACTTTCCGTGA

>AipsOBP14 ORF 759 bp

ATGTTTGATCCAAAAACAGTTTTTTATTTGTTGACCGTGTTCAGTGTTTGTTTTGGTGCTGTTGATATTAGGAAATATCTAAAAGTATGCGACAGAAATGCAATAGATGTGAGCGACTGTTTAACGGATGCAGTACAAAAGGGCATAGCTGTTATGGTAAATGGCATCGAAGAGCTTGGAGTGCCACCCATAGACCCTTACCTGCAAAAGGAGTTCCGAGTGGAGTACAACAATAATCAGATCGCTGTTAAAATGGTGATTAAAAATATATACGTGGAAGGTTTGAAGGATGCCAAGGTCCACGATGCCAGATTGCGAGCCGATGATGACAAGTTCCATTTAGAAGTGGATATGACCAGCCCACACGTGTTTGTCAAAGCCCATTATCATGGCGAGGGGCAGTTTAATTCCCTAAAGGTCGTAGCTTATGGCGATTTCAATACCACCATGAGTGATTTAGTTTACACCTGGAAGCTCGATGGAGTCCCAGAGAAGAACGGCAGTGAAACATATGTGCGAATTAAAGAGTTCTACATGAGGCCCGATCTTAGTAGCATTGTGACTTCGTTCAGAAACGAAAACCCGGAAACAAGAGAATTGACTGAATTGGGGGCGAGATTCGCGAATGAAAACTGGAGAACATTATACAAAGAATTTCTACCTTACGCTCAAGCCAATTGGAATAGAATCGGCGTCAGAATTGCAAATAAATTATTTTTGAAAGTTCCTTATGATCAACTGTTTCCATCGTCTTCATGA

>AipsOBP15 ORF 429 bp

ATGGATCATAACAGATTGTGTTTATTGGTTATCGCCATGTTTTTGGCTACAGGAAGTGATGCTATGACTAGACAACAATTGAAGAATTCGGGGAAAATACTAAAGAAAAATTGTATGAATAAGCATCAAGTTACAGAAGACCAAATAGGTACCATAGAAAAGGGAAAATTCGTAGAAGACAAGAAAGTTATGTGTTATATAGCTTGCATCTATGAATTGACGAGTGTGATAAAGAACAATAAATTAAATTACGAATCCTCCTTGCGACAAATCGACATCATGTACCCAGCAGATTTAAAGGAATCTGCGAAAGCTGCAGTCGAAAATTGCAAGGATGTTCAAAAGAAATACAAGGACATATGTGAAGCTTCATTTCATACGGCAAAGTGTATGTACGACTTCAAACCGGAAGATTTTATATTCGCATAA

>AipsOBP16 ORF 516 bp

ATGTTTCCAGGAAGTATTCCTTTTATTTCCGGGTGTGTGCACCTAGGAGTGTCCAACTATTTTCGATCTACTCAATCAAACTTAGTCGTGCACTACGAAGACGATCAGATCGTTGATGCTATTTACAACTGTCAAGACGAAAACGGATTCGATGAAGTCCTCTCAAACAGCACTAATTTAGAAGAAAATTTTCCTGAAAAAGAAGGTTTAAAAAAATCCAACGACTGCTTCTTGAAAAAAACCGGTTTCGTCACTAGTGATGGGAAATTAAATATCGATAAAACTTTAGAAAAACTTCCTCCTAGTTTTGTTAAGCCTATCGTTGAGCATTGCCAGGCAAATATAGCTTTAAACTATACGACAGAGAGTGTCGAGAATTTTTCGTCGTGTTATCACGATGGAATCCTGAACCACATTTTTGCGGCAACAGAAGTAGGAATTTTTCCGTTCATTCAAACTTGGAAATTCTTTGTGCCAGGTACTTCTTTTGCAGATACGATTCTAATTTTAAACTAA

>AipsOBP17 ORF 423 bp

ATGAATCAGTTATTAGTATTTGTTTTAATAGTCGCTTGTGTAAGAATCAGTAATGGGATGACCCGAGAACAAGTAAAAAAGACAATGACCGTAATAAAAAAACAATGTATGCCGAAAAATTCCGTAACAGAAGATCAAATTGGCAAAATAGAACAAGGTGTGTTCAATGAAGATCGCAATGTTATGTGCTATGTCGCTTGTGTCTACAAGAGTCTTCAAGTGGTCAAAAATGAAAGATTAGATTTGGGGCTCATATCGAAGCAGATTGATGCTCTCTATCCTCCAGAGCTAAAAGAGCCTACTAAGAAGGCCGTTTCACAGTGCATAAATATCCAGGATAGCTACAACGACTTGTGTGAAGCAGTATTTCATTCTGTAAAATGTTTGTATGAAAAAGACCCAGCTACTTTTATTTTTCCTTAA

>AipsOBP18 ORF 402 bp

ATGAAGACTTTATTTGTGTTTGCTGCGTGCATTTTATTAGCTCAGGCTCTGACAGACGAACAGAAGGAGAAGCTTAAGAAACATCGCACAGAGTGCCTGACTGAGACCAAGGTTGAGGAGGCCCTGGTCAACAAGCTGAAGGGAGGTGATTACAAGACTGAAAGCGAGCCCCTGAAGAAGTATGCCCTCTGCATGATGACAAAGTCGGAGCTGATGACGAAAGACGGCAAGTTCAAGAARGATGTTGCTCTCGCTAAGGTTCCTAATGCAGCTGACAAACCTGCAGTGGAGAAGCTGATCGATGCCTGCCTGGCCAATAAGGGCAACACACCCCACCAGACTGCCTGGAACTACGTCAAATGCTACCATGAAAAAGATCCCAAACATGCGATTTTTTTGTAA

>AipsOBP19 ORF 426 bp

ATGTTCACAGGGACGGTGCCCTTCGTCCTCTGTTTGGTTGCTGTCGCGTTTGGCGGTAAAGACAAACCTGTTTTCAGTGAGGAAATCAAAGAAATAATACAAACAGTTCACGACGAGTGTGTCGCTAATACAGGGGTGGCAGAGGAGGATATAACGAATTGCGAAAACGGCATATTTAAGGAGGACCCTAAATTGAAGTGCTATATGTTTTGCCTGATGGAGGAAGCGAGCCTCGTAGACGATGATGGTACTGTGGACTATGACATGCTGGTCAGTCTGATACCAGATGAGTACTACGAGAGAACTACCAAAATGATATTTGCGTGTAAACATCTCGATACCCCGGACAAAGACAAATGCCAGAGAGCATTTGAAGTGCATAGATGTTCGTATGAAAAGGATCCAGACTTATACTTCTTATTCTGA

>AipsOBP20 ORF 465 bp

ATGTTAGTAATCAATGCTACAGATTACGATTACGAAGGCTACGGAACTGGGAATATGGGTGAAAAACTGCTCACTTCCGTCCCGCGTCCCGCGTCTTCAAGCAATAACATTAACAATAATGATACAAGTCGGACAAGAAGAAGCGAACCACTTTTGAACAAACCAGATTTGGACCAGTGCCTCAGTCAATGTGTGTTCGCGAATTTGCAAGTTGTAGACAGTCGGGGAATACCGCGCGAAGCAGAGTTGTGGAATAAAGTGCAGTCTTCTGTGACGTCGCAACAGTCTCGTTCTGCACTGCACGACCAAATACGAGCGTGTTTTCAGGAATTACAGTCAGAGGCCGAAGACAATGGATGCTCTTACTCGAACAAGTTAGAACGATGCCTAATGCTTCGCTTCTCGGATCGAAAAGTTGAAGGTAAAGCAAGTACTCCCAAACCGGCTTCCACGGAACAATCGTAA

>AipsOBP21 ORF 435 bp

ATGTTGAAGTTCAGTGTTGTTTGTCTATATTTTTCGGTGGCGGCTGTTAATTTTTGGAATGTTCATTGCATTTCCGAAGATGAGAAAAAAGCCTTCATAGAGGCAATGAAACCGATGGTGGAAGAATGTGGATCTGACTGTGGTCTTACCGAAGAGGACTACAAGAAACACTCCAAAGGCGAGGACATGGATCCGTGTTTTAAGAAATGTATGATGCAGAAATTGGGTTTTTTGGATGAGGATGGCAAATACAATCGGAAACAGTTACATGAATCGATATCGGAATACACAGGAGACAAAGATGAAGCTAAGAGAGTACAAGAGCAATTAGATAGCTGTTTTGACGCGAACGGAGATAATGATGGTGATGATGAAGAGTCACAGATGAAACGAGTCGACGTTTTGTTCAAATGTCTCAAAGAAATTAAAGAGTAA

>AipsOBP22 ORF 414 bp

ATGAGCATGTGGTTCCGAGCGATGGTGGTGGTGGGCGCGCTGGCGGCGGCGCGCTGCGGGGTGGTCATGGACGAGGACATGGCGGAGCTGGCGCGCATGGTGCGCGAGAGCTGCGTGGACGAGACCGGCGCCGACGTCAAGCTCGTGGAGGCCGTCAACGGCGGCGCCGACCTGATGGAGGACGACAAGCTCAAGTGCTACATCAAGTGCACCATGGAGACGGCCGGCATGATGTCGGACGGCGAGGTGGACATCGAGGCGGTGATGGCGCTGCTGCCGCCCGAGATGGCGGAGCACAACGGGCCGGCGCTGAAATCGTGCGGCACGCAGCGCGGCGCTGACGACTGCGACACGGCCTGGAAGACGCAGGTGTGCTGGCAGAACGCGAACAAGGCCGAGTACTTCCTCATATAG

>AipsOBP23 ORF 438 bp

ATGTCTAAGTTCACGTATTTAGTTTTGTGTTTTGTGGCTGTGAGCAGGGTTTATGCCAATGAAGATGAAAGAGCAGCTTTTCATGAAGCCGCTAAGCCTATCTTAGTAGAATGTTCGAAGGAGAATGGAGTCAGTTTTGACAAACTCAAAGCAGCTAAGGAAGCCGGCAGCGCAGATGGCATTGACCCTTGTTTCTTCAGCTGCGTCTTCAAGAAAACTGGAGTCTTCAACAGCAAAGGAGATTTCGATTTGGATAACTCTCTCACAAAGCTCAAGGAGTTTGTGAGCAATGACGAAGATTATGCTAAAGTGGCTGAAGTTGGAAAGAAATGCGAATCAGTGAATGAAAAGGACGTAAGTGACGGAGAAGCTGGATGCGAGAGGGCTTCGTTATTGACTGCTTGCTTCTTGGAACACAGAGCCGAGATCCCCGTTTAA

>AipsOBP24 ORF 501 bp

ATGGCGAAATTATTACTAGCCATGATTCTAACAGTGATGACATTCGCTCTAACGATGTCAGCGACAACGAAAGATGCGGGGACGAAGGAAGCCATCATGACGACCACTGTGGCCAACCAGGATAGCAGCATTGACAGCAATGATGTCGATGTTCTAGCTGTCATGAACGTCTGTAATGAGAGTTTTAGGATAGAGATGTCCTACATACAAGCACTGAATGAGAGCGGCAGCTTTGTAGATGAAACTGATAAAACACCTAAGTGTTTCATCCGTTGCGTGTTCGAGAACGTCGGCATTGTATCGGAGGATGGTCGCATGTTCAATCCTGCGCGGGCTGCGGTCATATTCGCAGGAGAACGGAATGGCAAGCCTATGGATGACATCGCAGACATGACTGCTCTTTGTGCCGCTGACCGAAAGGAAACTTGCCCTTGTGACAGATCGTACCAGTTCCTGAGATGTTTGATGTCGATGGAGATAGAAAGATACGAAAAGTCCTAG

>AipsOBP25 partial 357 bp

AGCCGAAAGCTTCGTGAGGCTATGCGCCCTATAATAGAGCAGTGCTCTAAGGAGCACGGCGTCACCGATGCTGACATCCAAGCCTCTAAGGACTCCAACAACGCCGCCAGCCTCCCGGATTGTTTCAACCACTGCCTGTTCGAGAAATCCGGATTTATTGATAAAAATGGTCGTTACGACCGGGATTCGGGTCTGAAGAATCTGAGCAAGTACTTGAAGGATGTCAACCAGTATAACAAAGTGGTGGAGGTTACTAAGGAATGTGCATCAGTTGAGGAAAAACCCGCAACCGGATGCGAGCTGGGTACTCGTTTGACAGCCTGCTTATTGGACCACCAGACTAGCATCCTCATCTAA

>AipsOBP26 ORF 450 bp

ATGTCCAAGTTCACCTGCATCGTCCTCTTTGTGGTGGCTGCGAGCCTGACCAAGGTTACGCAAGCCGTTTCGGAAGAAGAGAAAGCTGTAGCCCGTGAGGCAATGGCTCCGATTCTAGCTGAGTGCTCGAAGGCAGAAGGAGTAAGCGACGAGGACATCGAGGAAGCCAAGAAAAATCCTAGTGTTGATGCCGTTAATTCTTGCTTTATACGATGTGTCATGCGGAAAACTGATGCGTTGAATGAAAAGGGCTTGTTCGACTCAGATGCTGCACTTGCCAAAATCAGACCGTTCGTGAAGAGCGATGAGGACTTCGCCAAATTCGAGGAGATTGGAAAAGCCTGCATGTCTGTAAACGATAAAGAAGTCAGTGACGGTGAAGCTGGCTGCGATAGGGCTAAGCTTCTCCTAGCATGCTTCTTGGAACACAAAGCTGAAATGCTTTATTAA

>AipsOBP27 partial 495 bp

GACAGCGCAATATCAGCTGATGCCGAGTCAAGATGCCGAAACCCGCCCACCGCACCGCAAAAGATAGAACGAGTCATCACTCTGTGCCAGGATGAAATTAAACTCTCCATACTTAGAGAGGCGCTAGACGTGATCAAGGAGGAGCACACGATGCCGGCACAGAGGAGACGAGACAAGAGAGAGGTGCCCTTCACGCATGACGAGAAGAGAATCGCTGGGTGCCTGTTGCAGTGTGTGTACAGGAAAGTGAAAGCCGTGGACGGCTACGGTTTCCCGACCCTGGAAGGTCTGGTGGGTCTGTACTCTGACGGCGTGAACGAACGCGGCTACTTCATGGCGGTGCTCGAGGCCTCCCGCGAGTGTCTCATGAAGAACCACGACAAGTTCTCCAGAACTATGCCCATGGATAACGGCCGCAACTGTGACGTGTCGTTCGACATCTTCGAGTGTATTTCGGACCGTATCGGCGAATACTGCGGAACTTCGGGCCTGTAA

>AipsOBP28 ORF 642 bp

ATGACTCACATCTTCTCTTCTTTCATTCCTTACATGATCACGGTCTCTATGTTCTCTTTCCCTGTCTCTGTAAAAATCATCTCGCCGACTGTACCTGTCACGGTCGTCTCTTCTTCGGTCATCATTACGTTCATGTTCGACATGTATCGTCTTGTGATTTTGAGTATTGTTGCAGTCACAACTGTGGTTGCTGACACGGACTTACAAGAATGCAGACGTTTGGTTCACCCTCACTCAATGCGTTGTTGCAAGAAAAGTGCTGATGCTAAGGAGAAAATGATGAAGAATGATGATTTGAAAGAATGCTTCGATTTGCCGAAGGACCCAGTCAAATGCGAGCACGAATTGTGTATGGCTAAAAAGAAAGGCATTACAACATCCGATGATAAATTAGACAAAGCAAAATTCGAAGAAGTTGTGACCAAAGATATAGATGACAAGGATCTAGTTGCGGATATAAAAGCAAATTGTATCAATGGGGACCTCACAAAATATGGACCCCCGGACTTTTGTGATTTTGTTAAAATGAGACATTGTATGTCTATGCAAATATTGAACCACTGCACCGAATGGAACGACTTTGGCGACTGCCCACAACTTAAATCGATTATAGGGGATTGTGTAAAACTAGTTGCTGCTTAA

>AipsCSP1 ORF 375 bp

ATGAAAGCCGTCATAGTCTTGTGTGCCTTGGTAGTGGCTGTCTGCGCTCGCCCAGAGGAAGAGAAGTATCCAGACAAATATGACAATACCAACTACAAGGAGATCTTGGAGAACGACAGACTGTACCGGGCCTATTGTGACTGCTTACTTGATGCTGGCAAGTGCACTCCTGAGGGCAAGGAACTCAAATCTCGCATCAAGGACGCTCTAGAAACCAAATGCGAGAAGTGCACAGACAAGCAGAAGGAGGCTGTCCGTTACGTCATCAAATACCTGATTAACAAGAAACCTGAAGACTGGAAGAAAGTTTGTGACAAATACGACCCTGACGGTAAATTCAAATCCCAGTACGAGAAGGAACTTAAAGACTTGTAA

>AipsCSP2 ORF 360 bp PG-specific expressed

ATGAAGATCATTCTAGCTCTATGCGTGTTGGTCGCGGCGGTGTCAGCTTACGACACCCGGTACGACGACTTTGATGTCGAGACGCTGGTGGGGAACGTTCGGCTGCTGAAGTCTTACGGACACTGCTTCCTGGGCACCGGACCTTGCACTCCTGAGGGAACTGACTTCAAGAAAACCATCCCCGATGCACTGCAAAGCGGCTGTGGCAAATGCTCGCCGAAACAGAAGCACCTTATCCGTACCGTAGTCAAGGGATTCCAGACTAAGACCCCTGACATCTGGCAACAGCTGGTCAAGAAGGAAGATCCCCATGGCGAGTACAAGGAGATCTTCACCAGGTTCATTAACGGTTCAGACTAG

>AipsCSP3 ORF 387 bp

ATGAACTCCTTCATCGTTCTGTGCATCGCTTCCCTGGCCGTCATGGCCTACGCCCGCCCTGAAGAAGCCAAATACACAGACCGTTACGACAATGTCGACCTGGATGAGGTTCTCAGCAACCGCCGCCTGCTGGTGCCCTACGTGAAGTGTATCCTCGACCAGGGCAAGTGCGCACCTGACGGCAAGGAGCTCAAAGAACACATTCGCGAAGCTCTAGAGAACGAATGTGGCAAGTGCACTGAAACGCAGAGGAAGGGAACCCGCCGTGTCATCGCACATCTGATCAACAACGAGGCTGACTACTGGAATGAGCTGACTGTCAAATACGACCCTCAGAGGAAGTTCACCGCCAAATACGAGAAGGAACTCAAAGAGATCAAGCAATAA

>AipsCSP4 ORF 363 bp

ATGAAGGTAGTTCTACTCACCCTTTGTTTCGCTCTCGGCGTGCTTGCTCAAGACAAGTACGAATCTGTCAACGACGACTTTGATGTCTCAAAAGTTCTGAATAATGACAGATTACTGCAATCTTACGCGAAGTGCTTGCTGAACAAAGGACCCTGTACTTCAGAAGTAAAAGAAGTTAAAGCCAAACTTCCGGAAGCTTTGGAAACTCGTTGTGCTAAATGTACGGACAAACAAAAACAGATGGGCAAGGTATTGGCTCAAGAAGTGAAGAAAAATCACCCTGACATCTGGAAGGAACTGGTGGCTATGTACGACCCTCAAGGCAAATACCAAGAGGCGTGGAAGGAATTCCTTCAAGAATAA

>AipsCSP5 ORF 324 bp

ATGCAGATCAAATACGCGCTATTGTTATGTTGCGTCGCTGCAGTGTCAGTTGCGCAGACGCAGCGACCTGCCGTGTCTGACACCGCCCTGGAAGACGCCCTTCAAGACAAGCGCTTCATTCAGAGACAGCTCAAGTGCGCGCTAGGCGAAGCACCCTGTGATCCTATCGGAAAACGGCTAAAGACTCTTGCGCCGCTAGTACTTCGGGGAGCCTGTCCTCAATGCACGCCACAAGAAACTAAGCAGATACAGCGCACTCTATCGTACGTTCAACGAAACTTTCCACAACAGTGGGCCAAAATAGTTCGCCAGTACGCGGGATAA

>AipsCSP6 ORF 384 bp

ATGAAGCTGATAATCGCAGTTGCTTTACTATGCATGGTGGCGGCTTCTTGGGGAAAGCCCGCGTCTACGTACACCGACAAATGGGACAACATCAATGTTGACGAGATCCTGGAGTCACAGCGTCTTCTCAAAGCATACGTGGACTGCCTGATGGACCGAGGACGATGCACTCCTGATGGAAAAGCCCTCAAGGAAACCCTCCCCGACGCCTTGGAGAATGAGTGCAGCAAATGTACAGAGAAGCAAAAGTCTGGCTCAGATAAAGTTATCAGGCACTTAGTGAACAAGCGCCCGGACTTGTGGAAGGAGTTGTCGACTAAGTACGACCCTGACAACATCTACCAGGACAAATACAAGACCCAAATTGAATCTGTGAAGCAGTAA

>AipsCSP7 ORF 387 bp

ATGAAGTTCGTCTTGCTATTGTGTGTCATGGTGGCTGTAGTCTACGCTGAAGACAAGTATACTGACAAGTTCGACAATATTGACCTGGATGAGATCCTGACCAACAGACGACTCCTCCTCAGTTACTTCAACTGCGTGATGGGGAAGGGAAAGTGTACTGCTGAAGGCAAGGAGCTCAAAGATAACCTGGAGGATGCCATTAAGACTGGCTGCGCAAAATGCACGGAGAACCAAGAGAAAGGATCGTACAGAGTCATCGAGCATCTGATCAAGAACGAACTGGACCTCTGGCGTGAGCTGTGCGCCAAGTTCGACCCCACTGGCGAATGGAGGCAGAAGTACGAAGACCGTGCCAGGGCCAACGGCATCGAGATCCCCAAAGACTAA

>AipsCSP8 ORF 372 bp

ATGAATTTTCTCGTGTTGTCAATGGTGATAGCCCTGGCGGGTTTTGTGGCAGCTGAAACTTACACAGATAGGTACGACCACATCAATATCGACGAGATCATCGAGAACAGGAAGCTGCTGGTTCCCTACATCAAGTGTACCCTCGACCAGGGAAGATGTACTCCTGAAGGAAGGGAGCTGAAAGCACACATCAAGGACGCTATGCAGACTTCGTGCTCCAAATGTACTCCAAAACAAAGGAAGGGGGCCAGAAAAGTAGTCAAGCACATCAGGGCTAAAGAACAGGAATACTGGAATCAAATCCTCGCTAAATACGACCCTGAAAATCAGTACTCTGAAAACTACGAAGCCTTCCTGGCCGCTGATGATTAA

>AipsCSP9 ORF 447 bp

ATGATGATGCATTCAACATTATCAATGTTGCTGGTACTATACTTGACAGTTCAGAGCAAAGCCATTGAGACGCCAACTTACACGACCAAGTACGATGGCATCGATCTAGACGAGATCCTGAACAATGAGCGACTGCTCACCGGCTATGTCAACTGTCTGATGGACCTTGGACCCTGCACCGCGGATGGAAAGGAACTGAAAAAAAACATCCCCGATGCGATTGAGAATGACTGCAAGAAGTGTACCGACAGACAACGTGAAGGTTCCGACAGAGTGATGCACTACCTTATAGATCACAGACCTGATGACTGGGTCAAACTGGAGGAGAAATACAATTCCGATGGAAGCTACAAAATGAAATACCTATCAAGTAAACCAATTGAAGAAACTAAGGAATCAAATGTCACAAAGTCTGATGAAGACACTAAGGATGCCGCTAAAGAATAA

>AipsCSP10 ORF 366 bp

ATGAAGGCCGAATGTGTTCTCCTCGCTACTTTAACAGTAGCAGTTGCAGCAGACTTCTACAGCTCCAAATACGACAGCTTCGATGTCCAGCCCCTGCTGGAGAATGATAGGATACTCCTCAGCTACACCAAGTGTTTCCTCGACCAGGGACCGTGCACGCCTGATGCTAAAGATTTTAAGAAAGTAATCCCAGAAGCCTTACAAACGACTTGCGGCAAGTGCACTCCTAAGCAGAAGCAGTTAATCAAGACGGTGATCAAGGAAGTCATAGCAAGACACCCTGAGGCGTGGGAGCAGATCACCGATAAGTACGATAAGGACAGGACATATAAAGAATCTTTTGATAAGTTTTTAGCAGAGGAATAA

>AipsCSP11 ORF 897 bp

ATGCGTGCAGTGCTATTATTGTGTGTTTTTGTGTACACTGTGGTGGGACAAGATGTAAATCAAATGGAAAATTTACCGAAATACGATTCGAGATATGATTATCTGGACATTGATGCGATTTTTACTAATAAAAGACTAGTTAGGAATTATGTGGATTGTTTGATCAACGCTGTTCGGTGCACGCCTGAAGGGAAACAGCTGAGAAAAATTCTTCCCGAAGCCCTAAGAACGAAGTGCATCCGCTGCACAGAGAGGCAGAAGAGAACAGCAGTGAAGGTGATCAAACGGTTGAAGTACGAGTACCCCGACGAGTGGGCCAAGGTGTCCTCCCGCTGGGACCCCACCGGAGACTTCACCAGATACTTCGAGGAATTCCTCGCTAAGGAAAGCTTTAACTCTATTCCCGGATCAGGGATCGCGATCCCCACGTCACAACCGCTGGCACCACCGGCCCCCACGATGCCTCCCACAGTCGCCAGCCCGGCTCCTGCACCCACGGAGCCTACGCCACCTAGACCTGTTGTACTTAATAGGTTTGGAGACGGCGAAATAATGCAGGGCAGTCCATCATCAGCCGGCGTGACACCAAGCCCGATGACTCAGGCTACCACGAGACCCACAACCACCATGAGGCCAACCATGAGGCCTAGTATGAGGCCAACCATGACACAACCCGCCACAAACCCAACCAGACCAACAATAATGACGATGACATGGGCCGGCGCAGCATCGAACACGCAGCCGACGCGTTTCCCACTACGTCCCGTGTCGGATCTCCCAACTCCATACTCCACAGCCATCACCATAATAGACCAGATTGGAATCAAGATCATCAGGACCACAGAACTCTTCACTGACATACTGAAGAACACAGTGAGGGCTGTCGTGGGTCGATAA

>AipsCSP12 partial 360 bp

GTCCTGATAGTGTTGTCTTGTCTGGTCGTGGCGGCATTCGCAGCTGATAAGTACAACGCCAAATATGATAACTTCGACGTGGAAACGTTGATCTCCAATGACAGACTTCTCAAGGCCTACATCAACTGCTTCCTGGACAAGGGCCGGTGTACACCAGAAGGGACAGATTTCAAAAAGGCCCTTCCCGAAGCCATAGAGACCACATGCGCCAGGTGCACTGAGAAGCAGAAAGCCAATATCAGGAAGGTGATCAGGGCGATCCAGCAGAAGCATCCCAAGGAATGGGAAGACCTGGTTAAGAGAACGACCCAACGGCAAGAACCGCTCCACTTTGACAAGTTCATCCAGGGAAGCAGATAA

>Orco ORF 1422 bp

ATGATGACCAAAGTGAAGGCCCAGGGCCTTGTGTCAGACTTGTTGCCCAACATCAAGCTGATGCAAGCGGCTGGGCACTTTCTCTTCAACTACCACTCAGAAAATGCGGGCATGTCAAACCTCCTCCGCAAGGTCTACGCCAGCACCCATGTCGTCCTCATCATCATCCACTTCGCGTGCATGGCTATCAACATGGCGCAGTACTCCGACGAGGTCAACGAGCTGACTGCTAACACCATCACCGTTCTTTTCTTCGCCCATACCATCATCAAGTTAGGCTTCTTTGCTTTGAACTCGAAGAGCTTTTACAGGACCTTGGCAGTATGGAATCAGTCAAACAGTCACCCTCTGTTCATGGAGTCAGATGCCCGCTACCACCAGATCGCGCTCACCAAGATGAGGAGACTGCTGTACTTCATCTGCGGAATGACTTGCTTGTCTGTTGTCTGCTGGATCACTCTGACATTCTTCGGGGAGTCCGTCCGCATGATCACGAACAAGGATACAAATGAGACCTTGACGGAGGTGGTCCCTCGTCTACCTCTGAAAGCCTGGTACCCGTTCAACGCTATGAGTGGAACCATGTACATTATTGCGTTCGCTTTTCAGGTCTACTGGCTCCTCTTCTCAATGGCCATCGCTAACCTGATGGATGTCATGTTCTGTTCCTGGCTGATCTTCGCGTGTGAGCAGCTCCAGCATCTGAAAGCCATCATGAAGCCGCTGATGGAGCTCAGTGCTTCTCTGGATACCTATAGACCGAATACTGCTGAGCTGTTTAGAGCTTCTTCTACTGAAAAATCAGAAAAGATCCCAGATGCGGTAGACATGGACATCCGCGGCATTTACTCCACACAGCAAGACTTCGGTATGACACTCCGCGGAGCTGGAGGTAGACTGCAGAACTTTGGACAGCAGAACTCAAATCCTAACGGTCTGACACCGAAACAAGAGATGCTGGCTAGGTCTGCTATCAAGTACTGGGTTGAGAGGCATAAACATGTGGTCCGATTAGTGGCGTCCATTGGTGATACTTATGGTACTGCTCTGCTGTTCCACATGTTGGTGTCAACGATCACACTCACTCTGCTGGCCTACCAAGCAACTAAGATCAACGGTATAAACATATACGCTTTCAGTACGATTGGCTACCTCAGTTACACTCTCGGTCAAGTATTCCATTTCTGTATCTTCGGTAACAGGCTCATTGAAGAGAGTTCATCAGTAATGGAAGCAGCCTACTCCTGCCAGTGGTACGACGGCTCTGAGGAGGCCAAGACCTTCGTCCAGATCGTGTGCCAGCAGTGCCAGAAGGCTATGAGCATCTCTGGAGCGAAGTTCTTCACGGTCTCACTGGATTTGTTTGCTTCGGTGCTTGGTGCTGTAGTAACTTACTTCATGGTGTTGGTGCAACTCAAGTAA

>OR1 ORF 1308 bp

ATGCAAAGACTAAATAAAGTACTTTTTGAAGATGTGGCTCTTTTGGGTGACATCAAAAAAGTTACGGAATTTAAATACTTGAAAATCCTACGTTTCAATCTGTCATTTGTGAACGCATGGCCCGCAAAAGAGATAGGAGAGAGTCACTCAAAGTACATAGGATTAAAAAGTTGTGTACAAGTTATGGTAAATCTATTTTGCTTTGTTACTGGTATGATTTTCATGGTGAAAAATTGGCATATGAGCTTTTACGAACTTGGACATATGATGATAACTTTGATGATGGGATTTGTTGCTTTGACGAGAGTTTTACTCACGCTTCCCCAGTCAACAAAATACAGAAACCTGACAACTATTTATCTGACTAAAATGCATTTACTATTCTTCAAAGATGTGTCTGAATATGCAATGAAGACGCACAGGAAAGTACATTTTTTGTCCCATCTATTTACGCTGTATTTGACAGGACAAATGATCGGTGGTATTGTCCTGTTTAATGTTATTCCAATGTGGAGCAACTATTCTTCTGGAAAGTATAAACAAAAAATCCTCTTAAATTCAACGTATGAACATTCCTTATATCTTTCTATACCCGTACTTAAAGTTTTTACACACTTTGATGCGTACATCATTGGTTGTATTTATAATTGGTTAATGTCATACTTATGTTCAGCTCTTTTCTGCATGCTGGATCTTCTACTATCACTGATGGTTTTCCATCTATGGGGACATTTGAATATTTTACTCCACAATTTGGAAACTTTTCCATTGCCTGCAAAGGAAGTTGTCTTATCAATTGAAGATGGATACACTAGCGTCTCTGCAGAAATGTATTCCAAAGAAGAATTGAAAGTAATTAATCAAAAGTTGACACAGTGCATAGAACACCATCGGCTTATCAAAGATTTCACTGATCAAATGTCAGAAGCTTTCGGTCCAATGTTGTTCATTTATTACGCGTTTCATCAAGTCAGTGGGTGCCTACTCCTTCTCGAATGTTCTCAAATGACACCAGAAGCCTTGATGAGATACTTACCGTTATCAGTAATATTGTTCCAACAATTAATTCAGCTTTCAATCATTTTTGAGCTGGTAGGGAGTACGAGCGATAAACTAAAGGGCGCCGTCTACGGTGTGCCATGGCAATATATGGACTCAAAGAATAGGAAGGTAGTTGGCATATTCCTGTATCTCGTTCAGGAACCGATACATGTGAAGGCACTTGGCGTTGTCAATGTTGGGGTCACTACTATGGCCTCGATTTTGAAAACATCGCTGTCTTATTTTACATTCCTTCGCAGCATTTAA

>OR2 ORF 1308 bp

ATGCTTGCTAGAATTGAAAAAATAGCTGGAGAACCCGTTGTCGGAATAAACGGACCCATGGATTACACATATATGAAGGCACTACGTTTAGTACTTCGAATTATAAGTGGCTGGCCAGGAAAAGCCCTTGGGGAGAAAATATTAAAAATCGAGGGATGGGGCCATGCGTATTACAATACGATATTGTCGCTAGTATATCTGGCATTAGGCGTTGCTTATTTGAAAAAGAATAGGCACAAGTATGAGTTTTTGGAACTGGGACAGTTATACATTGTGTTGCTGATGAATGCGTTGTGTACGTCACGAGCTTTCACGTTGTGTCTATCGGAGAAGTACCGAGTTGTGGCAAAGAATTTTATCCAAAACATTCACCTGTTTCATTTCAAAGATAAATCTGAATTTGCTATGGGCATTCATGTATTTGTGCACAAACTATCATATTTCTCGACGGTATATTTAACTTCGTTGTTGATATTAGCGGCCTGTATGTTCAATTTGATCCCGATGCACGCGAACTATAGCTCCGGCAAGTACAAAGATTTGGAGAATAGGACTTACGAACAAGCCATCAACTGCCTATATCCTTGGAACTATGAGACGAGTATGGCCGGCTACGTTGTGGCTAATTTAAGTGGCTGGTATGGCACATTTTTGTGTGGAAGCAGTGTATCGATGTTTGATCTATTTCTCTGTTTAATGATCTACAATTTGTGGGGACATTTTAACATTCTCATTCATAACTTGGATCACTTTCCGAGACCTGCCGCGGAGGTAGTGGACGGAGCTGGAGAAGCGATGAGTAGCATGAGGGTCGGACCGGAGATGTATTCACAAACTGAACTAAATCAGGTCGCGATATTGTTGAAGGAGTCCATACAATATCATAAACTTATCTACGATTTCACAAACGACATGTCGGAGGCATTCGGAATGGCGCTCTTCATTTACTACTCTTTCCATCAAATCACCGGGTGTCTGCTTCTGCTGGAATGTTCTACAATGACCGCAGCAGCATTGTCGCGTTACTTGCCCCTCACACTTATTATGTTCGGAGAACTAATTCTGCTTTCCATCATTTTCGAAACTATCGGCACCATGAGTGAAAAATTAAAGGATGCAGTTTACAAAGTGCCATGGGAGTACATGGACACCAAGAACCGCAGGACAGTCCTCATTTTCCTCATTAAGGTCCAGGAGCCGATTCACGTAAAAGCTGGAGGTTTAGTCGATGTGGGGGTCACTACTATGGCTTCAATCCTGAAAACATCGTTCTCATATTTTGCTTTCCTGCGTACCTTTGATAATTAA

>OR3 ORF 1272 bp

ATGAAATTATTCTCTGATCTGTCTGACCTGGAAGGAGTCGAGAGAGCAGAAGATATTCCGTACATGCAAATTCTTAGAAAGAGTACATGGATCATAGATTCATGGCCAAAAAAACCTGGTGTAACTGGGTACTACAGATATTATGCGATTATTCTACAGATAAGCAGTCTTATACCTGGCATCATATACTTGAAGAACCACACTGGTAAACTGTCCTCTTTTGAGATGGGACACACCTACATTACCGTGTTTATGAATAGCATATCTCTTACTAGAGCTTTGATGATTATCGGTCCAAAATATAATGAACTCCTTAACTATTTCTTGGATGAAATGCACCTTTTTAATTACAGAAATAAATCGAAGTTCGCTTATGAAGCTCACATTCTGGTTCACAAAATATCTCATTTCTTCACGGTCTACCTCCTTCTGCTGATGTGTCTTGGTTTAATTTTATTCAACGTGACGCCAATGTACAACTGTTACTCGAATGGAATGTATCGAGATGTAGCTGATCGACCTCCAAATGCCACGTTTGATCATGCTGTCTTCTATGAATTACCTTTTGACTACACAACGAAAGTGGACGGATACATTGTCCTCTTTATGTTTAATTGGTACATCTCAGTTTCCTGCTCGACCAATTTCTGTGTCGTGGATTTAACTATTTCTCTTTTAGTTTTCCACCTCTGGGGTCACATGCGTGTTTTAAAACAAAATCTCGAGAATATTCCGAAGCCTTCTGGGTACGTGGCTGCCTCAAATCATTATGAGGAAAAGAAGTATACCGAGGAAGAGGCGAAAGAAGTGCACAAACAATTAACAGAGAATATCCACTACCATAGTATTATCATTGATTTCCAATCGAGGATGTCCGAAACTTTTGGTGAAGTACTCCTGGTATATTTCCTATTCCACCAAGTAAGCGAATGTCTTCTGATGTTGGAGTGCTCTCAAATGAACCGGCAGTCACTGTTGCGTTATGGACCCTTGACAATAGTGATATTTCAGCAGCTGATTCAGTTAAGCATTATATTCGAGCTGTTAGGATCATCGAATGACAAGTTAATAGACGCAGTTTACTCTGTGCCTTGGGAACACATGGACACTAAGGACAAGAAGCTCGTGCTGAAGATGCTGGAACAGGTACAACGGTCCATGAACCTGAAGGCGATGGGCATGCTCACTGTGGGCGTCCAGACCATGATCACGATCTTAAAAACGTCATTCTCCTATTTCGTAATGCTACAAACAGTTGCGGAGGAAGATTAA

>OR4 ORF 1299 bp

ATGACTTTACGACAATTTCTTTTCGAAAACGAAGCAGTTAATGGCATCAACACACCATCCGATTACCTGTACATTAAAATCGTACGTTTCATGCTTATCACTATCAGTTCTTGGCCTCAGAAAGAACTTGGGGAGTCCGAACCGGCCTACCAAGCCGTCATGAAGATTTTCTACTTTGTGCTATGTATTTTTCTGAGTTTTGGATACGTTCTATATATCTATAACCACAATAGCGAACTTAACTTTTTGGAAGCTGGTCACATGTATCTTATTGTATTAATGAGTGTTATTGATATGTCCAGAGTTAAAACCTTGACACTTTCCGCGAAATACCTAGCGGTGGCTAAAGATTTCCTCAGAAAAATCCATCTGTTCTACCACAAAGACCGTTCTGTGTATGCGATGAAGACTCACAAGATGGTCCACCAAATATGTCATCTCTTCACGCTATTGTTACTAATTCAGATGTGGTCCGGTTTAACTCTATTCAACCTGATTCCTATATACAGTAACTATGCCGCGGGAAGGTACAAAAGCGGAGGAACTCAAAACTCTACGTTTGAACATGCTGTTTATTTACCGTATCCTTTCAATACGTCGACTGAAATGAAGGGTTATATTATTGCTTGCATTATTCATTGGTGGCTGTCATATCTGACTTCAACTTATATCTGCATGTTTGATCTCTTTTTGTCTCTAATGGTTTTCCATTTATGGGGTCATTTCAAAATACTGCTCAACCTTCTGAATGAATTCCCAAGACCAAGTTCAAGCACTAAGATCATTACAGAAGAAAATGAATTAGAAATAGAAGTTGAAATATATTCCAAAGAAGAGCTTTTAGAAGTTTCGGAAAAGTTGAAAGAATGCATTACTTACCACCGGGAAATTATCAAATTTACAAACACAATTTCAGACGTATTTGGACCGATGTTATTCGTGTATTATATTTTTCATCAAGCTAGTGGTTGTTTGTTACTCCTGGAATGTTCACAGATGACAGCACAAGCTCTGATGCGTTATTTACCTCTAACTGTAGTATTGACACAACAACTAATACAGATATCAGTTATTTTTGAATTAGTTGGAAGTGAGAGTGAAAAACTGAAAGACGCTGTATATGGTGTGCCGTGGGAATGTATGGATACGAAAAACAGGAAAGTTGTGATCTTCTTCTTGATGAATGTTCAAGAACCGATTCATGTGAAGGCACTGGGTCTTGCTAATGTTGGAGTCACTTCTATGGCCATGATTTTGAAAACTTCAGTGTCTTACTTCACCTTTTTACGCAGCATGTAG

>OR5 ORF 1209 bp

ATGGATAATTATAGCGGCAGCTACAAGCCCACAAAGACAACAGAGTTTCTAATCAATCTGAACAAATTCGTCTTCATATTCGGTTTGCCAAATTTTTGGGTTGAAGATCTAGATTTTTCAAAGAGATTCACGGAAATTATTCGAAAACTAAGTACAATTGGGAATATGGCAGTTTTCGTTATGATCATTGCAGAATATAGTGCTTACATTACGCAGAAGAACTTGACGGAGCGACAGAGCTCTGATTTGCTTCTCTTCATCATCTCCCATTCTATTATCACTGGATTTCGAGTTCGTATGACTCATCAAGAGAGGCAGATAAGAGATGTCATGTACAAACTGGGGATAGGACTGAAGGAGATCTACAATAATGGCGACGCAGAGCAGCAGATGATAAAACGTTCGAAGGTGTTTTCCTGGGCTCTCATCTTGAACTGCGTCATCTCTATCATCCTGTACACTATTGAGGCTATTCTTAGGGTCATACGTACGGGAGTAACATTCAACACAATAATCACTGCCTGGCCAGACGTCGAGGATAGATCTATGCTTGCCAACATCGGGAGATCAGTCTTCTACATATTCTGGTGGATCTACCTGTCACGTATCTTCGCGGTCTACACCCTGGTGATATGCCTGACCATCGCCGTCAGCCATCAGTTCAAGAACTTGAACAGCTACTTCCGAAGTTTGGAGGGTATCTTTGAAGATGATAGCCTGACGCAAGAGGAGAAGGAGGAGGAGTATGAAAGAGCTCTCAAAGTTGGCATCAAAATGCATTCGGAGACGTTGAAGTGTACCAGTGAGATACAGGCAATATGCCGAGATGTGTTCAGTGGACAGATTATATTCAATCTGACTATGCTGATATTGTTGATGTACCAAATGGTGAATTCTGCGCGTAACCTGACGAATGCTCTGACACTGGTGGTGACGGCACTCACGATTCTATTCAGCACTGGCTTCTTCATGTGGAACTCTGGTGATATTACCGTAGAGGCAGAGATCCTGCCGACGGCGATGTACAGCTCTGGCTGGGAGAACTGCAGACGCCACTCGTCGGTGCGCGTACGCAAGTTGCTCGTCATTGCTATGATGCAGGCACAGGAACCAGTGGTTTTAACTGGTCTTGGTATCATAGCGCTTTCGTATCAATCCTATGTGTCTATCGTGAAATCATCTTATTCAGTGTTCTCTGTGCTGTATTGA

>OR6 partial 581 bp

ATTTTTTATGGTTTACTTGCGTTGTACCGAGTGGTTATTGAAGGTGACAACTTCATCACGATCACAGCAGCCTGGCCTGACGTCCACGACACATCCACAGCAGCAGGAGTGATGAGAACCTTCTTCTATCTGTGGTGGTTTCCCTTTGCAGCCAGGATCATGTTCACCTTTATAATGCTGGTTGCAACAATGCTGGCTATCTGCTACCAGTTCAAAAACCTGGAGGTTTACTTCTACAACCTTGAAGATATCTTTAGCGATAGAAGACTGAGTCAGGCTGAGAAGGAAGTGAAGTATGAAGAAGCATTTAAAGTTGGGATGCAGATGCATTCACTGACTTTGTGGTGTAAGAAGCAGCACCAGCACATCAGCAAGGAGATGTTCGCCGTGGAGATCATTCTGTTCTTTGGCATGCTGTTGACTCAACTTGTTACTTTACTGAGCGGCGCACATAATTTGGCGCAGCTATCCATAATGGTGCTAATGTCGATATCAACTTGTTTGTCCTTTGGCTTCTTCATGTGGAATGGTGGCGATATTACAATAGAGGCAGCCAAGTTATCAGATGCGATGTACAGTTC

>OR7 ORF 1179 bp

ATGGAAGAAGAGGTTGAATTTAAGCCGTTCAGCGACACATACAGAATGATAACGTTTGCAATGAGTGTGGGCATGATATACCCGAACGAGCGAACCGAGAGAGTGAGGCTGGCCAGCATACCGTTACTCATAGCGTCCATAGCTCCGCTTGCTACGATGATTCTAATCGACATTTTCAAATGCTGGTTAAGAAAAGACATCGTAAACATCATCAGGCATAGCACAGTTGTGGGACCATTTCTTGGAGGATTCTTTAAGATGATCCTGATGTACCACAAACGAGTCCAAGCGAAACAGATCCTAGACGAAATAGACCGCGACTATCATCTTCTCAACGACTTTGCAGAAACATATAAGGTCATAGGTCGCGCTTCTATAAAAAATAGTCAGGTTTATAGCGAAAGATGGTGGGTTAATACTGTGACGATTTGTGTCATGACCTTTCCCGTGATGGCTATAGCATCGACCGTCTATAGTTTCCTGTTTACCTCTGAACCACAAAAGTATATGATACATGACTTGGTGAAGCCGTATGGAGATCCCGAAGCGAGATTTGTTACGCCTTATTTTGAGGTCATGTTCTTCTACCAATTCTACTGCTCGATTCTCTACGTGGTCAACTTTATTGGTTACGACGGGTTCTTCGGGTTGGCGATCAACCACGCTTGTCTGAAGATGGACCTGTACTGCAAAGCTTTAGAGGCAGCTATGCAAGCGCCGGAAGACGAGATTTGTGGTCAAGTCATCGCTGTTATCAAGGAGCAGTGTCGGTTGTTTAGATACGTAGATGTTATTCAAGATACGTTCAACATTTGGCTGGGCATCATATTCATAGCGACCATGATACAGATATGTACTTGCTTGTATCATATCACTGAGGGCTACGGTTTCGATCTACGCTATATGATATTCGTGACCGGGGCCGTTATTCACATCTACCTGCCGTGCCGGTATGCAGCTAAGTTGAAAGCTATGTCGGTGGAAACATCAGAGCGACTGTACTCCAGCGGCTGGGAGCACGCCGACGAGCACAGAGTCAAGAGGATGATACTATTCATGGTGGCGAGAGCACAGCAGCCCATTGTGATCACCGCTTTCAATATGCTGGCGTTTGACATGGAGCTGTTCACTTCGATCCTGCAATCGTCGTATTCAATGTTTACGCTTTTGAGGTCATAA

>OR8 ORF 1185 bp

ATGGAATTACATCAAATAGACTGCTTTAAAATAAACATGAAGTTTTGGAGGTTCCTTGCTATTTGGCCCGGTGACAATTCCACTAGTACTTACAGCTACTATTCCAAAGCATTTCTTGTTACCTTCGTTTTTGTCTACTATGTCCTATTCTCCGTTAACTTCTATTTCTTGCCGAGACGATTGGATATTTTCATTGACGACTTGATGTTTTATTTCACCGACTGTTCCGTTTTATCTAAAGTTCTGACGTTTCTATTGTTGCGTAAGAAAATCATTGCGATATTAGATGTTCTGGAAAGCGAAATATTCCAACCTGACGATGCTGAAGGGAAAGCTATTATAGAAAAAGCGAAGACGTTTAACAAGTTGTACTACAAAGCTGTAGCATTTGTGTCCGTTACATCAAATGGTACACTGCTCTCTCCGTTAATAGTGCATTTTATAAAGAGAACACAACTAATATTTCCGGTCTGCAGTTACGGTTTCTTTTCTGAGTCTTTCAGAGACACGTTTGTATATCCTTTATATTTCTACCAGAGTGTCGGCATAACTTTTCATATGCTCTACAATTTGAATGTTGATACTTTTTTCTTGGGATTATTGGTTTTCACAATAGCTCAACTGGATGTCCTAGATGTGAAGTTAAGGAAAGTAACTGATGATAATAAACCTGATGACGGGGATGGTCAAGTATCTAGGGAACCAACTGACAGAAATAAGGAAGCTGTTTTGAAAATTAACAAATGTATCGTACATTTTGATGAAGTGGGCAAGTTTTGCGATCTCGTAGAAGAAGTTTTCAGCGTGACACTATTTGTGCAATTCAGTATGGCATCCTGTATAATTTGCGTTTGCTTGATGAGATTTACTATGCCAGCACCATTGGACTATTACTTATTTCTTGGTACCTACATGTGCGTGATGATTCTACAAATTATGGTTCCTTGTTGGTTTGGTACTCGGATTATGGACAGGAGCAATCTACTAGCTTTCTCCATCTACAACTGCGACTGGACTGCTCGATCGCGTCAATTCAAGAGCAATATGCGCCTCTTTGTGGAGAGAACTAACAAGCCTCTGTCTATCACTGGAGGCAAGATGTTCTGTCTTTCTCTTCCCGCTTTTACCTCTATTATGAATTCCGCTTATTCTTTCTTCACTTTGCTACAACAAATGCAAGACTAA

>OR9 ORF 1251 bp

ATGAAAATCATAGAGACTCTTCGCAAGTTCGGCCTGGAGCACTGTGATGTGGGGACCATGCTGTGGAATGTATCAGTCTTGCTGAGACCTCTGACCCTGAATGTTGATATACGGAATAAGAAACCGATCTCTTTAATCTTGTACATCGTGGCGGTTTCCGTGGCCGTGGGTTACTTCTACGTGTACGTGGTCTCCTTGATCTGGTTCTGCTTCATCCACTGCCCTGCCACCGGTGACCTGCTCGCTGCCATCATCGTCTTCTCTCTGGGGGTCTCCAGCGAGATTGGAACTGTTAAATTGGTGTACATGTTCATGCATATTGGCAAAGTCAGAGATATAGTGTCAGAATGCCTAGCTTGCGACTCCTCAGTGGTGCCAGGAAGTCGAGTCTCCACCAATCTGATGAGCACCCTGAGGCAGGTGAAGAAGAATGCCATGATGTTCTGGCTGGTCATCATTGCCAACGGCGTGGTGTACGTACTGAAGCCTTTGCTACTTCCCGGGAGGCATTTCACTGAAGACATGTTCATACTCTTTGGTTTAGAGCCAATGAAGCAGAACCCAAACTATCAAATAGCAACAGTACTATTTTTTTTAGCTACAATCTTCTCCGTCTACCTTCCAGCAAACATGACAGCCTTTCTCATAGTCGTCACAGGGTATATCGAGGCTCAAATGTTATCTTTAGCTGAAGAATTAATACATCTCTGGGAAGATGCTGAAGAATATTATTACAAAACAAAACAGGATATGACTGTATTAGATAAGAATGAAAAAGAATTAGAGCGCAAAAACAAAGCTATGAATGATTACGTGGAAATACATCTCAAGGAGATAATAACGGCACATTCAAGAAATATCAATTTATTACATCAAGTTGAGAATGTGTTCAATGGAGCTATAGCATTAGAATTCTTTCTTCTAGTAATTGGTCTCATAGCAGAACTATTAGGAGGACTAGAAAACACCTATATGGAGATACCGTATGCTTTTATCCAAGTTAGCATGGACTGTTACACAGGACAGCATATTATGGATGCAAGTGATATATTCGAGAAAGCTGTGTATGATAGCAAATGGGAGAACTACAATGTATCCAACATGAAGATCATACTGACGATGTTACAGAGTTCACAGAAAACGATGAAGTTGTCTGCTGGGGGCATCTTCAAATTGAGTTTCAGTTGCCTGATGCAAGTCATAAAGTCAGTATTCTCATCATATACGACTYTGAGTGCTACCATGAAATAG

>OR10 ORF 1290 bp

ATGCGTCTTCAAATCATAAAAGATTTTCTTCTGAAGGAAACTTTTGATTTTGACAGGGAAGATATTAATCTGTATAATTTTCACCCACAGCTGCGGGTATTTCTTGTTTTCAATGGCGTTTTTTTTAGTAACCAACATTCTAAACTCAGATTCATATGGCCGGCTATTTGCATTCAACTGTCCATCGTCACAATGTCCCTGGAAGAAGTATTTATATGGCATGGGGTTACCACAAGAGATTATTCGTTCGCCACAGAATGTTTCTGCTACTGGGTAATCCTTGGATGCATTCCAACAGTCTATTGTGGGATAATCTTCAAAACTGACAGGATTCGAGATCTCGTGTTGAAGATGAACGATGATTTTATCTTCATTTGTAGTCTAGGACTTAAATACAGAAAACCTTTTCTAGAGGTTCAGCTCTTGATATGGCAGTTGTGCTACGCGTGGTTCACATTCGTCTCTATAGTAGGTGGCTTGTACGTGGTGTTCCCAGTAGTTTCTCTGGTCTACCAGAGCCTCTTCGCTACCATTGATGAGAACACCGTGAGACCTCTCCAGTTTCCCATGTGGCTCCCTCACGATGACCCCTACAAAACACCAAACTATGAACTATTCTTTATGATAGAATCGGCCATGTGTTTTATTTTTGTTCAAACATTTTGCGTATACATCTACACGCTATTTCACATACTCCTGCATTACTACGCAGTAATGAACATGATTATCATAGACTTCGACGTGATATTTGAGGGTCTGGACAAGTCCGTGGCACTGCTACCTCGGGATGACGAGCGGAGGATGGAGGCGCAATTCATACTCAACGCGAGGATTAAACGAATTGTCACCTGGCATCTCTCGGTGTTAAGGGCCGTCAGCACCGTGTCAACTGTTTACGGAATACCGCTGGTGTACCAGGTTTCCTTGAGTTCCGTCGCCATATGCCTCATAGCGTATCAGATTGCCGATAACATTGACCATGGCACATTAGACATAATGTTTTATCTGCTTGGTATAGCTGCGCTTTTGCAACTGTGGATACCATGTCATCTCGGCACCATGATAAGGAACAAGGCATTCGAAGTAGGAGACGCAGGTTGGACATGCGGCTGGCACGAGACGCCGTTAGGCATGATGATTCGAACTGACATCATCATCATCATCCTGCGAGCTCAGCAGCCTGTCACCATCGAGTTCACCGGGTTGCCGCCTGTTCAGCTTGAAACTTTCTCCTCTTGTATGAGTTCGTCATACTCATATTTCAATATGCTACGTCAATACAGCAAATAA

>OR11 partial 480 bp

CATATACAGCCAAGAGTACGACGATGCGACGGTGTCCGCACTCAAAGACTGCGCAGCCCTCTGCCAGGTGGTAACCAATTCAAGGTGACGTTTGAGAAGTTCGTGTCCCCTCTCCTGGTGCTCAGAGTGGTGCAGGTTACTCTGTATCTGTGCACGTTGCTGTATGCTGCTACGGAGAAGTTCGACATGATAACAGTAGAGTATCTCGCAGCTGTAGCGTTAGATATGTTCGTGTACTGCTACCATGGGAACCAGATCATTTTGCAGGCTGACCGCGTCTCGACTGCAGCTTACCATAGCATGTGGCATACGATGGGTGTTCGTCCACGAAGAGTTCTACTGAACATACTGCTGGCTAACAGACGACCAGTCGTCGTGAGAGCTGGCCGGTTTCTGCACATGGACTTGCATACTTTTGTTGTTATAATAAAGACTTCATTCTCTTACTACACGCTGTTAGTCAACGTTAATGATAAGTAG

>OR12 partial 861 bp

AAAAAGGATAAAATAATAAAGTATGAACATAATTTCGTGACTACAGTCATATCAGTTTTAAACGTTTTCTACTTCGTCTTGATAGTAGCATTTGCATTGAGTCCTTTAACTTTAGTGGCTCTGAAGTATTATTCTACTAATGAGCTTGAATTACTCTTACCATTCCTTATTATTTATCCTTTCGATGCTTATGATATTCGATATTGGCCCTGGGTGTATCTTCGGCAGGTTTGGTCTGAAGTAGTCGTGGTTATGGATATCTGTTCTGCCGATTACATATTCTACACTTTTTGCACGTACATCCGGATGCAGTTTCGTCTTTTAAAACATTGCATTGAAAGATTTATTTCTGATGATGTTGTTGATGGGAGATTGCGCAACATTCAGGAGACAAGAGCTGAGTTTATACTTTTGATCAAATGGCATCAAGAATTGATACGATCAGCAAATATGTTGGAGACCGTTTACACAAGATCAACTTTATTTAATTTCGTTGCAAGTTCTGTCCTCATCTGCCTTACGGGGTTCAACGTTACGGCAATCACGGACATAGCATTTGTGGCAACATTTCTGTGTTTCCTGTTTATGAGTTTACTACAAATCTTCTTTTTATGTTTCTTTGGCGACCTTTTGATGACATCGAGTATGGAAGTCAGCGATGCGATATACAATTGTCGATGGTACTTAGCAGATAAACCTCTTGGGAAGGATTTACTTTTGGTGCAAACGAGAGCTCAAACTCCGTGCAAGCTGACAGCTTCTGATTTTGCTGATGTAAATCTTATGGCTTTTATGAAGATTTTGAGCACAGCCTGGTCTTACTTCGCCTTATTGCAAACATTATACAGCTCACCAACTTAA

>OR13 partial 495 bp

AGAGCTAATATTTTATTTCACAAACGTCGGAGCTTTGTCGAAGGCATTGGCATTCATTTTCTTGCATGGTACTGTAAATTAATTCAGGTTGCCTTCAGTGAGATTTTATTTGTGTTATTCAGTTCTGGGTCCTGTAAGATATGCATGTGCCTGTTCCGGTTTACGATGCCTGCAGAAACGAAATATTTTGTTTTTCTGACTCTGTATACTGTTGTGATGACTCTTCAAGTTATGGTTCCTTGTTGGTTTGGATCTCGGTTAATGGAAAAGAGTAGTCAAATAACTTTTGCAATTTACGATTGTGATTGGACGCCACGTTGTCGCAGATTCAAGAGCAATATGCGACTTTTAGTTGAAAGAGCTAACAGACCCATTACGATTAGAGGAGGAAAGATGTTCTTACTTTCACTTGGGACATTCACTGCGATTATGAATTCTTCATATTCGTTCTTACTTTACTGCGTCACATGCAATCTCGTTAGGTATTTCTCGTAA

>OR14 ORF 1248 bp

ATGCTACGGCACCTCTTATCAGTGATAAGCTCCTGGCCTCTGAAGCTAGTGGACCCACTGGACACTGATGCTGTACAAAGAAGAAGAATCTGGGTATATACCCAGCGGTCGTTCCATTTGGTTTTGGTTTTGATTACTTTTGTTGGCGGAGCGATGTTTGTATTGCTGCATATGGATTCTATGTCTCTCTTTGAACTTGGTCATTTGTATATTTCCCTGTTGATGACTATTTTGATTTTTTCTAGGATCACAACATTGTGTTTTTCTAATCAGTACGCAGCCACTGCTAGACTGTTTTTAGAAAAAATCCATCTGTTTTTCTACAAGGAGCATTCTGAATTTTCCATGAAGACTCACAAGCAAATTCACCGAATCTCTCACCTTTTCACATTGTATCAAATTGTCTCAATGATAGCTGCCCTCGCCCTCTTCAATTTAGCTCCGATATACAATAACTGGTCTGCTGGAAACTATGGCAGGAGTGGCATACAGGGAAACGCCACATATGAACAAGCTCTTTATTTCTCATATCCTTTCCCTGTGTCGACAGATTTCAAATTTTATCTTCTTGCTAACTTTTTTCATTGGATTATCTCCTATTTATGTGGAAGCTGGTTTTGCCTGCACGATTGTTTCCTATCCTTAATGGTGTTCCATATCTGGGGACATTTCAAGATTTTACTTTATAATCTGGATAACTTTCCGAAACCCTCTTATAAGACTCTTTTCAAGGTCGAAGAACAAAGCAATGCAAATCGAATTTGTGAAATGTATTCTCCACATGAGCTCATAAAAGTTAGTGATCAGTTAAAGGAATTGATAGAATTTCATAAAAATATTGTTAGTTTCACAGATGAAATTTCGGGTGTGTTTGGGCCAATGTTGTTAGTTTATTACGGATTTCACCAGGCAAGCGGATGTTTACTCCTGCTGGAATGTTCACAAATGACGGCCGATGCTTTGATCCGCTATCTACCGCTAGCAATTATCATATTTGTACAACTTATCCAGCTGTCTATTGTCTTCGAACTTATTGGCAGTATTAGTGACAGATTGCAAGGCGCTGTATACGGCTTACCCTGGGAGTATATGGATACAAGGAACAGACGAACTGTGGCGTTYTTCCTCATGAATGTGCAGAAACCTGTACATGTGAAGGCACTAGGGTTAGCTGAAGTTGGTGTTACTTCTATGAGTACGATACTGAAAACTTCGATGTCATACTTCCTTTTTCTGAGTAGCAAATGA

>OR15 partial 183 bp

TGTCCATGGTACGAGCAGGACACCAAGTTCAAGCGAGCTCTCTTCATAGCCATGGAACGGATGAAGAAACCCATCATATTCAAAGCGGGACACTACATCTCACTGTCTCGACCAACATTTGTTGCGATTCTACGTTTGTCATACTCTTACTTCGCAGTCCTAAACCGGGTGAACACTGAATAA

>OR16 partial 693 bp

TATGTGGATGCCACTAAACCCAGAGAAACGGTTCAGTACTACATCGGATACGCCTTCCAACTGGGGACCATCTGCATCAGCGCTTACATGTACTTCGGAGTGGACAGCGTCGCTTTCTCAAGCGTCATCTTTGGGTGTGCCCAGATAGATATCATCAAGGAGAAGATATTGAATATAACATCGGTAGATAAAACACGTGGTACAAGGGAGGTAGATGAAGCCTTAGCCGACAACTACAACAAGCTCGTTGATTGTATCAAACACCATCAAGCAATTGTTAAATTCACAGAGCTAGTGGAAAATGCTTACCATCCATATCTGCTGTTCCAATTAGTAGGCAGCGTTGGAATCATTTGCATGTCCGCATTAATGATTTTAGTCGTTGATTGGCAGAGCATGCAGTTCTTCTCCATCCTGACTTACTTGTCAGTAATGATCAGTCAACTGTTCGTGTGCTGCTGGTGTGGGCATGAGCTCACTGCTACCAGTGCTGACCTGCACACTGTGTTGTACAAATGTTACTGGTACGAGCAGGATGTGAAGTTCAAGCGCGAGCTATGCTTCGCCATGATGCGCATCAGTCGCCCTCTGGTGCTGCGCGCTGGACACTACATCATTCTGTCAAGGCAGACTTTTGTTGCTATCCTTCGAATGTCGTACTCCTATTTTGCAGTGTTGAATCAAACTAAGTAA

>OR17 partial 673 bp

ATTGCATACTTCATGATGATCGCTGGTGTCTTCTTCATCTGCTACGTCCCAGCCACCGTGACTGCCTTCCTCATCGTCATTACAGGATACGCCGAAGCCCAGATGAGGGCCTTAAGCGAGGAGATGCTGCAACTCTGGCCTGATGCCAAGAAATATGCTGAAGCAAGGACAGAACTGACAGCTGCTGAGGAGTTCAGCATTTATAACGCAGAAGTGAAGATACTCATAAATCAGTTCGTACAAAGGCGTTTGAGAGAGATTATTGGTCGTCACGCGAATGTGATCAATCTGCTCAATCAAGTGGAGATCGTTTTTCGGCAAGCTATTGCGATGGGGTTCCTGCTGTTGATTGGTGGGTTGTTATCTGAGCTGCTGGGGAAGCTGGAGAATACGTTTCTGCAGCTGCCTTTTGCGTTGATGCAGGTATCCATGGACTGTTTCGCTGGCCAACGGGTCATGGACGCCAGCATGGAGTTCGAGGCGTCAGTCTACGACTGCAAGTGGGAGAACTTCGATAAAGCCAACATGAAGCTGGTGCTGGTGATGCTGCAGAACTCCCAGAAGACCATGACCCTCTCTGCTGGAGGAGTGAGAGCCCTCAACTTTACCTCCTTGATGTCTGTTTTCAGAGGGATCTATTCTGCTTATACTGCTCTTCGGTCTACTATGAAGT

>OR18 partial 1080 bp

AACGTCCTCAGAAGTCGCTCCAGAAAAAAATACAAAGGGTTCAATGAAACTTTCAAATTGTGCGCTTTCTCACTAGCTTTCGCTTTCCTTTACCCAAACAGGAACACCGCTATCAAGAGATGTGTTACTATCACTGCAATCGTCACGTTCTGTGGTGGTCAGCTATTCTGGTTCATCACATACACCTTCAAATGTCTCTACACCTTGGACATCTACAATTTCGCCAGAAACATGACTTTGGCTGTCGTTCTTGTTCTTTTCTTTATAAAAACGTATTATGTTATATATGCGACGAGCAAATTCGCTCCGTTGCTAGACAAAATATCTGAAGACCTACTAGAGGCTAACAATCTCGAAGAGGAATTTCAAGCGCTGTACGATGAACACATCAAAATAGCAAAAGTTGGTGAGATATCCTGGTTACTCATCCCGACAATCATGAGCGCATTATTTCCTATATATGCTGGAAGCTTGATGACCATAGAAAGTATACAAACTGATGACTACGAGAGACGAATGGTGCACGACATGGAACTTCTATTCGTGGAGGACATTCAGAGTGAGACTCCTTTCTTCCAATGCATGTTTGCGTACAATTGTGTGCAATGCGTTGTTCTCGTGCCTAATTACTGCGGATTCGACGGTTCTTTCTGCATAGCGACGACACATCTCCGGCTCAAATTAAAGTTGTTGACTCTTAAAGTAGATAAGGCTTTTAAAACTTCTCGGAACCGTCAGGAGCTGCGGTTGAAAATGAACGAAGCAATCAGAGATCACCAGGATGCGCTAGATTTTTATGTTCAGCTGCAAAACGTGTACGGGCCCTGGCTATTTGCTGTATTTCTGCTAACGTCTTTCATGATATCTTTCAATTTATATCAAATATATCTACTGCAACGAATAGACCCGAAATACACGTCATTTGGGGTGGTCGGTGTCCTACACATCTACTTACCATGCCATTATGCAAGTGAACTGACGAAGACAAGTGAAGAGACTCCACTGGACCTGTACGTGGTCCCGTGGGAACGTTGGGCAGACAACGAGGTCACCAAGCTGCTGATATTCATGATCACAAGG

>OR19 partial 923 bp

AGACTTCTACAAGAATCTGGCGAGTTCCATGAATCCCCATTCTTCGATATATCAACTGAAGAGAGGAAGAAACTGGTTGAGTTCTGGTCGCAAACGAATGAGAGATATTTGAAGTTGTTGTTAGCTTTAGGGAACTGTACTCTTGCAGCTTGGTTCACCTTCCCCTTAATAGACGAAGTGGACTACAACCTTATAGTGGGGATCCGCCTCCCGTTCTTCTACAAGACCCGTATCCGGTACCCTCTCTCTTACATCGGCGTGGTGATATCTTTCTATTACATCTCGCACTTTGTGATGATAACTGATCTGAAGATGCAGACCCATCTCTTACACCTGCTGTGCCAGTTCACTGTGCTCGCTGACTGCTTTGAGAACTTGCTGAGGGATTGCAGAGCTGGATTTGAAGATGTAGCTGAAAACAGTTTAGTTTACAACAAAAGTTTCTCTGATAAGTACACAAAGAGACTCGGCGAGCTAGTTCAACAACACAAGCAAATATTAAGTCAATCGATGAACATGCGAGACACGCTGAGTGGCCCCATGTTGGGGCAGCTGGCGGCGAGTGGCACGCTCATCTGTTTCATCGGCTATCAGGCTACTACGACCTTAGACGAGAGCATCGCCAAATGTTTAATGAGTTTCCTGTTTCTTGGCTACAACCTCTTTGGTTTCTACATCATTTGCCGCTGGTGTGAGGAGATTACAAATCAGAGTGCAAGAATAGGCGAGGCCATATACTGCTCAGGGTGGGAGTGCGGAGTGACCAAACTGCCAGGAGTGCGGGCCTCCATACTGTTTGTGATGGCGAGGGCCAACAAGCCCCTAATCCTCACAGCGGGAGGGATGTACAACCTCTCCCTCACTTCTTATACGACTTTGGTGAAAACATCATACAGTGCATTGACGGTGCTGCTTCGTTTTCG

>OR20 partial 291 bp

TTCGCTGCGGTAATCACCGCGACCGCGGTGCTCATAGGCACTGGATTCTACATGTGGAACGCGGGAGACGTGACTGTGGAGGCTTCCCATCTGGGCACTGCGATATATTTCTCTGGGTGGCACAAACCCCACGGACAGAGTTCTGTGCGACTCCGGAACCTTGTCGTTATAACTATGAGCCATGCTCAGCGTCCTGTGCTCCTCAAAGGTCTCGGATACATAGAGCTGTCGTACCAGTCATATATTAGAATCGTCAAGTCGTCGTACTCCGTGTTTTCAGTGCTGTTTTAA

>OR21 partial 532 bp

CTTGATGGTGTACGCTTTATCGCACCCGATGCTGTTTTCCTTCGTCTGATGATGGCGAATATGCAGGATAAAGTGAGGCTGGTGATGTACAAACTGACGGTGGGGCTGAAGAAGGTGTACAACGACGACGAGGTAGAGAGACACATGCTCAGGAGTACGAATATGTACCTGTTCGCGATGGTGTCCAGTTGCGTGTTGTCTATGGTCATGTATGCGGCTGAAGCATTATGGGACGTCATACGATATGATGAGACTTTTACGACTTTAGTCACTGCATACCCAGATGTTGAGGACAAGTCTGACCTGGCCAACGTAGTGAGAGCCCTCTGCTTCATAGTGTGGTGGATCTTCCTCACCAGGATCGTGGGGGTATACATGCTAGTCATTGCCCTGAGCAACTGCCTCAGCCATCAGTACAAAAACCTGCAGGGCTACTTTACAAGCCTTAATGAAATATTTGAGAGAGAAGACTTGAGTCAAACAGAGAAGGAGCAGATGTATGAATCTGGCTTTAAAGTTGGGATTCAACTGC

>OR22 partial 138 bp

TTCCTTATACAGAGAGCTCAAAAACCTATAGCATTCACAGCGATGGGTTTTACTAACATTAGTCTCGTGACATATTCGTCGATTCTTTCGAAGTCATACTCATACTTCGCATTACTGTATACGATGTACAGTGAAAAT

>OR23 partial 915 bp

AACGGGATTTTTCATTTTATTTGTACGGACGGAGAAAAATATCGAGTCCGATATTTTGAGGAACAACTGAAAACTTGGAAGATCTGTTTATTCGTATGCTTATTCTCCGCTCTTGTGCCATTTGCGATGAACATATTCGCTTATCTTTCGCTCTTCTACTTTCTGTCTACTCATGATTCCGAAGCTGGTGGAAGTCGACCCCTTCTGTTTCCATTTTGGCTGCCTAACGTCGATTTTGGCAAATCTCCGGTTTACGAAATAGCGTTCATGTTTGCTAATATTTGTTGCGTATTGTATTCCTACAACTACGTTTTTATGATGCAAACACAAATTGTTTGGGTAAGACAGATAACTTCAAAAGTTGATATCGTAATATGGAGTCTCCAAGATCTACTTGCGGACATACATCCAGCCACTAGTAAAGAGGAAAGTAAACAATTCTCTTATTTGATTCAGACTCGAATGAGAGACATCGTGATGCATCATCAATTAATGTACAGCCTGCTAGAAGATCATGCGATTGTTTACAAGAAATTACTACTATTCGAACAAGCAATTTCTAGTCCCGTGGTTTGTTTGACAGCCTACTGTGTTGCAGTGAAATTGGACCAAGGTGAGCTACAAGCTATACTTTTAGTACTACTTATTGCTTGTCTCGTACTTAATTTTATATCAAGCTTTTTGGGTACCTACCTGACTTACAAGATACAATCAATTTGTGACGCATGTTGGGACATTCCTTTCTGGAACGCAGGGCCCGTGATACGTTCCTACTTGGTTCTTATAATACAGCGCTCATTACGGCCATTGCCCCTTCAAGCTCCTGGTTTTCAAGACGTTTCTATTGCAACGTTTTCTGAAAAAATGTCCTCTGCTTATTCGATGTTCAATATGCTACGACAAACTAATCTATAG

>OR24 partial 675 bp

ATCACGAAATACAACGGACAGAAAAAACTGGATCTTTTAGACGCTTTTACGAAGTATTGTAGAAAGATAACGTATCTATATTGGTCTTTGATGTATACAACGGTTATCATAGTCATGGTCCAGCCGATTTTCAAGTATGTTTCCTCGGAGACTTATAGAGAGAACGTAAAAAATGGGACGGAGACTTACTTGCAAGTCGTGAGCTCTTGGGTCCCTTGGAATAAAAACTCTATCGGCGGGTACTTAGCTGCCTCAGTTTATCAAAGTTATGCAGCTATCTACGGAGGCGGATGGATAACCTCCTTTGACACTAACTCTATGGTAATCATGGTTTTCTTTAGAGCCGAATTGGAGTTATTAAGAATAGATTCTATGAATATTTTTGGAACGGAATCTTCGCCAGTGGAACATGATGTGGCTATTGAGAGATTGAAGAACTGTCATCGGAGACATGTTATGTTGGTCAAGTTTGCTCGTCTCTTCGACTCTTGCCTATCACCAATAATGTTGCTATACATGTTCGTCTGCTCAGTTATGCTGTGTGTCACTGCCTATCAAATCACGATTGAAACCAACCCAATGCAAAGGTTCTTGACGACTGAATATTTAGTGTTTGGCGTTGCGCAGCTGTTTATTTACTGTTGGCACAGCAACGATGTTTTGTTTGCAAGTGCT

>OR25 partial 744 bp

TTTATTAACAGAGCCATCTACTGGGCAGCCGTAATCGGTACGGTCCAATACATGCTCTCACCTTTATTTGAAACTATGTTCCGGAGATTTGTATTGCAACAGGACTGCGAGCTGCGTCTACCTTTAGCTTCGAGCTACCCATTCAACCCGACTAAGAACTGGACGATTTATTTCTTGGTTTATATTTTCCAGCTATATTCTATGACCATTCTTGTCAGCGTATACGTAGGAGCAGCCTTGATTATGGCAACGTCTTGTGCACTACTTGGAATACAGTTTCTGATGCTGATACATGATCTAGAGCAGGTCAATCCGAAGCTCAGTAGAGGAAGACATTACACAGATGAGGACGGACTTAGTATTGAAACGGTTGTGGAACGACATCAGATACTATTGAGGTTATCGCGGCACCTGGATAACGTTTTCAACGGATTGGTATTCATAGATCTATTGTTTGTTGGGATCACGACTTGTGCTTTCAGTTTCATGGGGCAGTTTTCCCGCGGTCCGGGCTACATGTTGCTGAGCTACATTGGCATAGCCTCGTCCATGTCGACCATTTTGTGTTTATGTTACTACGGAGAGATACTTACTAGAGCGAGCACCAGTATCGGAGACACGGCGTACAATAGCTTATGGTATGAAGGTGGTAAACAATACAAAATGACGATACTGCTTATCATCAGAATGTCGCAAAACCCATGTCGTCTTACGTCCTTGAAATATGCTGTGGTGTACTTTTGA

>OR26 partial 648 bp

AGAAGAAGAAAATTGTACAAAAAGAAAGTGATTGACACTCATGTGTTAATGTTGACTAACATTATGAGGAAGATAAGGAAATTGATTATGGTGGGTCTGTTGATGTTTGCACTGACACCTGTTTTTATAAATCTTTCGGATTATTTCAATAATTATGAAGTCAAACTTGAAATGCCGTTTATAGCATACTATCCATTCAACGAATTCGATGTTAGAATATATCCTTGCGTTTATTTTCATCAGGTGTTAACAGCGTGTTTTGCTATTCTCATGGTTTATGGTCCCGACTGCTTATTTTTTACTTTTTGTACATTTCTCCATATTCAGTTTTCATTACTGAATTACGACATGGAGAGAATTGTAAGTGAAGACACTTATAGATGTGATGGACTAAAATTCAAAAAGTTAGCTGTGCGTCATATCGAATTAATGAGATGCGTCAATTTGTTGGAGGGAATATTTTCGAAATCAATATTATTCAATTCTTTAACAAGTTCTGTAATTATATGTGTAACAGGATTTAATGTTTTGGTGGTTGATAACATAGTTATGATGGCATCTTTTACAGCGTTTTTAATATTTGGGTTGATACAAATATTTCTTTATGTTATTATGGGGACACTATTAGGACATCGAGCATGCAAGTGA

>OR27 partial 220 bp

ATCTGCTGCATCTTGACGGAGTTGTTTTTGTACTGCTACTATGGAAACGAGGTCACCGACGAGAGCGAGCGCGTGTCGGGGTCGCTGTACTGCATGGAGTGGCGCAGCGCGCGGCTGTCGTTCCGGCGCTCGCTGGTGCTGGTCATGGAGCGCGCCAAGCGCCCGCTGCGCCCCGCGCCGGCCTCGTCATCCCGCTCTCACTTGATACCTTCATCAAGAT

>OR28 partial 411 bp

GCAATTCCAGAAGAGATTGAGAGACTTGGTTACTCGACATCAGGCGGTTTTAAACAAGCTGTAGACTTGAAGGGAATACTCAGCGCTCCAATGCTAGGACAGCTAGTCTGCAGTGGATTAATCATCTGCTTGGTCGGCTATCAAGCTACAGCAACGATGGCAGCAAACGTGGGAAGATTTTGTCAGTCGCTACTATATTTGAGTTACACAATGTTCTCTTTGTACCTCCTCTGCGGATGGTGCGAAGAAATCACGTCTCAGAGTCAGCACATTGGCGAATCAGCGTACTTCTCTGGCTGGGAGAGTGGCATATCCCAGGCGCCTGGAGTTCGAGCAGCCATAATGGTGGTGATCGCCAGATCCAACACCCCGCTCGTGTTCTTTGCGGGGGCATGTACCCGCTTTCATTGA

>OR29 partial 1177 bp

GAAGACCCGGAGAAGCCATTCTTAGGTCCAAACCACTGGATTCTAAAGAAAACTGGATTACTGCTGCCTCAATCCTTGCTAGGAAAAATAACGTACATGTTCATACACGAACTAGTTACGTTTTTCGTAGTAACACAATATATTGAACTGTACGTCATAAGATCGGATTTAGATCTCGTGTTGACTAACATGAAGATTTCTATGTTGAGCGTCGTTTGTGTCGTGAAAGCGAACTCTTTCGTCTTTTGGCAAAAACACTGGCGAGAAGTTTTGGACTACGTCACAGAAGCTGATAAATTTGAGAGACAAAGTGATGATCCGGTCAATACTAACATCATTACAGTTTACACTAGATACTGCCGTCGCCTCTCGTACTTTTACTGGGCCTTGGTCTTTACAACATTCTTAACAACAACCTGTTCGCCGCTGATGAAATATCTGACTTCTTCTACTTTTAGAGAAAACTTGCGAAATGGGACTGAGCCCTTCCCGCACATCTTCAGTGCTTGGATTCCCGTTGATAAGTATCATTCACCTGGATGCTGGATCACGGTAGTTTGGCATACACTTCTATGCTTCTATGGTGCTGCAATCATGGGTGCGTATGATACAAGTGTCGTGGTTATCATGGTGTTCTTTGGTGGAAAGCTGGACTTGCTACGAGAAAGATGCAAGCAAATGTTTCGGTCGTATGAAACTGCGATCAGTGATGATGAATGCAAGCAAGTGGTGCAACAACTTCACGACATACATGTCGCAATGATCAAGTATTCGAGACTATTTAATTCCCTTTTGTCGCCTGTCATGTTCTTCTACATGGTCATGTGTTCCTTGATGCTTTGTGCGAGCGCCTACCAACTAACCTCTATACAAAACGCCGCACAGAAGCTACTGATGGCTGAATATTTAATATTTGGAATTGCGCAACTCTTCATGTTCTGCTGGCACAGTAACGACGTTTTGATAAAAAGTACAAACCTCTCATCTGGGCCCTTTGAAAGTAACTGGTATTTGGCGAACCACCGACAAAGAAAATATGTGTTAACTCTGTCAGGACAGCTCTCTATCGAGAACATGTTCACTGCCGGACCTTTCGCCAACCTTACTTTGCCAACCTTCATTAATATATTGAAAGGAGCGTACAGCTACTACACACTGCTGAAAAAGTAAAATAGAT

>OR30 partial 645 bp

GGTACAAGGGTAACTTTGTCGAAGACAACATTAATTTTGATGCCGATGACAAGCCCTTATTACGAAGTCGGATTGTTCCTGCATACTATTTATTTGGTAGAGATGGCATTTACTTATTGTTTGGTAGATCTGTGGTTTGCTACGTTAATGCTTTCTTTCTGTATGGCGAGTGACAGTGTAGCAAATGGGTTGAAGATTAGGCCTAAAGGATCCGATGAAACAGAAGTTGAATATATGGATCGTCTGAACAACACGCTCAGGACTTTTTATAAAAATCATGCAATTTTGATGGATTATTTTAATATTACAACGGATATGTTCAAGTGGCTGACAGTAATTCCTTTACTAAGCACTTTATTCGCAGTATGCTTGATCATGCTGTGTATGGCTGAGCAAATACAATGGATGTTTTTAACGAACTTAGTACCTACACTGGTAGAAATATTTGCCTACAACTTACTTGGTGAAGCAGTAAAGAGTAAGGAAACACAATTCTACATGGCTCTTATTAAGTTCGACTGGGCAAGCATGCGTTTCAAAGATAAGAAGAACTACCTAATTATTATCAGTTATATGAACAAGGAGTTCAAGATTAAGACCGCTGTGGGAACGACCTGTCTCTTGTTACTATCACCTCGGTATTAA

>OR31 partial 239 bp

TGCATGGATGCCTTTCGATCCAATGAAAGCACCATGAACCTGATATTGTCACTAGAAATAATAGCATTCTGCGTCTTTCCTGGACTCTGCAGAGCGTTTGCCATGCAAGGTTTGGTCTGCAGTATGATCATGTACTTGTGCGATCAACTGATTCATTTGCAGACAGAGCTAGGGAGCTTGAAGTATGTGAAAGAAACTGAGATGGCGACGAGGATGAAGTTTAAGAAGATTATAAGGAA

>OR32 partial 451 bp

ACATTGTCAGTGAGCATATCTCTGACTGTCCTACAGCTGGTGGGGTCGTGGGCACCAGAATATTTGTCTGCAACACAGAAACAGTTGTATAGTTTCTACTCCGTGTTCTCTTTCATGTTTCTATTGGGAACCTACCTAATCATCCAAGTAGTAGACCTAATCCTGATCTGGGGAGACCTGGCTCTGATGACCGGCACAGCGTTCCTGCTGTTCACCAACATGGCACAAGCAGCCAAGATCGTGAACATCCTCGGAAGGAAGGAGAGGATACAGAGTATTGTCAATGACAGTGACAGGGTGCTCTCAGAAGCCAGGACTTGGGAGGAGAAGGAGATTGTTAAGAGTTGTAACCGAGAAATGATAGTACTACAGGTGTTATACTTCGCACTGACACTAATAACAGGTCTCGGCTGGGCCACCAGCGCTGAGAAGCAACAACTGCCTCTGAGAG

>OR33 partial 537 bp

CATTCAGGAAATTGTGCACACTTTGGAGATAGGGATGTACGATTACTATCAAAATGTAGCAAAGAACAGAAGAACGGTAAAATATCCAAAATTATAACTGGAGCCTTTATTTTCTTCGGATGGCTTACGATTGGCAATGGAAATGTGTATGGGACGATTCAAGACATCAAATGGAAGTCTTTGGTTGCTACTTTAAACGATACTGAATCGCGTCCAGTACGGACGTTGCCACAGCCGATTTATATACCCTGGGATTATCAGAAAGATGCTTCATACATTCCTACTTTCGTTCTTGAAACTGTAGGATTGTTATGGACTGGTCATATCGTGATGACCATTGACACATTTATTGCGTCTGTTATCTTACACATGGGTTCGCAGTTTGAAATACTAAACGAGGCTATAATAACTGCGTATGATAGGACAATGAAGTCACTAAGGGAAGGCCTACGTCTCGATGCTAATGTTGTTCAAGAGAGAGAATCAGTGTTATTGAGTCCAGAAGATAATGATGAGAAATTGTGGAAGCTTTTGTAA

>OR34 partial 603 bp

TTCTGGAAAAAGGAGGTTTTTCTAGAATATTTAATGATGATAACCTTACGAGAAATTGAACATGAAATCCGCAAGTTTGGCCTCGAGTACTGCGACCTGCCGACCATGTTGGAGAATGTTTCCATCTTGTTGAGGGTCCTCACTGTCAATATAGACAGCAAATATCATAAAGGCATCACCATCTACTCGTACATCCTGACAGCAGTGACGGCAGTGTGCTTCTACTACGTGTTCCTCTTCTCCATGTCTTGGTTCGTGTTCTACCGCTGCCGCATCACCGGCGAGCTCGTGGGCGCCATGGTGGTGCTGTCGCTCGGCATCTCCAGCCAGATCGGGCCGTTCAAGCTCTTCTACATGTGCTTTTATATGGACAAAACCCAAAAAATAGCCGACGGCTTCTTGGAGTGCGACGCCAACACGATCAAAGGCACCCGGTTCCACGAGAATTTGATGAAGTGCTTGAGGAGTGTGAAGAAGCGCGCCATGCTGTACTGGGTCGTGGTCGCTGGCAATGGAGTCTTGTATGTGATGAAACCTGTCGCCATGCGGGGAAGGAATTTACCGGAGAACTATTTTATTATTTATGGCTTAGAACCAATGTTC

>OR35 ORF 1182 bp

ATGCTGTGGAACGTCTCCGTCTTTCTGGAGGTCCTCACTCTCAATATATACGGCAAGAATAAATCAGGAATCCCCTTCATGTACTACATCATAATGATCGCAGCCTCCCTCTGCTACTTCTACACGTACCTGTTCAGCATGATCTGGTTTGTCTTCGTGAGGTGCTGGGAGACGGGCGACATGGTGGCAGCTACGGTGGTGCTGTCTCTGGGAGTCTCCAGTGAGATCGGAACCCTAAAGCTGCTGTATGTGTTCGTGAACATCGACAAAATCCGCGGCCTCACAGATGCGTTCCTAGAGTTCGATTCCCGCATGATCCCGGGCAGCCGTCGGGCCACCAACCTGCTGAAGTACATGCGCAACGTCAAGCTTCGTGCCATCATCTACTGGGGGGTGCTGATGGGCAACGGCTCCTTGTACGTGTTCACCCCCTTGGTTCGCCCTGGTCGACATCTCACTGAGGATTTGTTGATCATTTATGGAATGGAACCTATGATGGAGTCCCCAAACTACGAACTCGCATGGATCATGATGACTACCAGCGTTTACACAATCTGCTACATTTCAGCCAACGTCACCTCGTACCTCATCGTCATTATAGGGTACGCTGAGTCCCAAATACTGACCATGAGTGATGAAGTAACTCACATCTGGACTGACGCCGAAGAACATTACAAAACCGTAGCTAGATTAGGAAACGTCAATTTCGGTATTGACGAAAAAAGGAGAATTATGAATGAACACGTTTGTAATAACCTTAAAGACATCATCAAACGCCATGCAACGATCAAGATTCTTCTGAACAAAGTAGAAGACGTTTGCAGTGGACCTATAGCTGTAGGATTCACCTTGCTAGTTATAGGATTGGTATCAGAACTGCTTGGAGGACTAGAGAACACCATCTTGCAATTACCATTCGCATTCATGCAAGTTGGTATCGACTGTTTTATTGGCCAGAGGGTAATGGATGCGGGTGAAGCGTTTGAGCAAGCTGTGTATGATTGTAAATGGGAGAATTTCGACAAGAATAACATGAAGATGGTGCTGGTGATGCTGCAAATTTCGCAGAAGCCCACGGCTATATCTGCAGGGGGAGTCACTAAGATGAGCTTCAGTTGCTTGATGGCGGCGATGAGGGCTACGTACTCTGCTTATACTGCTCTTAGGTCTTTTATAAAGTAG

>OR36 partial 312 bp

ACCTGTGGACAACTTGAAATACTTAGTCGACGCATCTGTAAGTTATTTGTTGAAGCCGAAGATAACAAAGAAATAGAACAAGGATTGAAACACATTATTGCAAAATTGCAGGATCTATACAAATTCGTTGATCGTGTAAAAGCCAATTTCTCTATTTTGTATGAATATAACATGAAAGCAACAACATTTTTATTACCACTAACAACATTCCAAATAGTTGAAGATTTGCGAGATAAACGGATAAACGTCGAGTTTATTTCTTTCTTTGTTGGTTGCATTTTACATTTCTACATGCCTTGTTACTACAGCGAC

>OR37 partial 417 bp

AAAAGAGATTATGATATGTCCAAACACATGAATTCTACTGAAATTGGAGTGTTGAACAAATATGTGAATCTAAGCATTTGGGTTTGCAAACAATGGCTTGTTATTTCCGTATCAGGTACTCTAGCCTTTTTACTCAAAAGTATTGGACTTATGTTGTACTATTATATCATTGGGGAATTCAAACTAGTGCATTTTCACGAATTGGTTTATCCATCTTTTATTGAAAATAATAAAGATAATTTCTTCTTTTTTTTGTTGACTTATACTGAGATTTTATTTTATGGTATGTATGCGTCTTGTATGTATGTTGCATTTGTACCTTTGGGTCCAATTTTTATGCTTCATGCGTGTGGGCAACTTGAGTTGGTGGAAAATCAAATTGATGATCTGTTGTGGAGTGTGATGCTGAAGTTATAA

>OR38 partial 312 bp

CCTTTGGTGAAGGGGAGATCAGCTTGGAATTCATTACGTTGTAGTGGGAGGCGCTTTGATCAGCAGTTCGCCTTGTATTACAGTGAATTGCTTATGGAAAAGGGTGAGGCATTCCGCCAAGCAGTATACTCCTGCGGCTGGGAGCGTCTGTATGACCGGCGCGTGCGCACTACTATAGCACTGATATTACAGCGAGCGCTCCGTCCCACAGCTATACGGACGATGTTCAGAACTGTCTGTTTGGATGCACTGGCTGATCTTTTCCATCAGTCTTACGCCATATTTAATCTCATGAACGCTATGTGGAATTGA

>OR39 partial 267 bp

ATGGCCGCCATCATGGGCGACTATCCCGAGGACGAAAAACGTGACTCGGTGAGCTTCGCCGTCAGTCACACGATAGTTATGATCAAAATATTCTCGGTTATAACCAACAAATCGTTGATAAAAGACCTGAATCGTAAAATGGTGACAGTGTGCGAGAGTTACGAAGAAAACGCGTTGATGGCGAAGAAATATAAGATCATGAAAATAAATGTTGTTGCTTACGTTTCGATCGTTTATGGCTCATGTTTTTTTTTGTTTTTGAAGGAT

>OR40 partial 402 bp

ACTTTCCTTTCGTTCCACTGGGTTCTATCCTTTGCTGGAATTCGCTTTTTCGCCATAAAAAAATGGAACTCAAAACCGTGGTTACTTTTTCAAATCTTCAACTTCATAATCGGTGTATTCTGTTTCATTTTCACAACGGGTTTTGTCGTTATTAACGTCTCAAATTTCCTCCTCTGTATCCAAGGAGCTTGCATTTGGACAACAGGAGTCATCATGACGATAACTTTGGGGGTCTGTCTCGTTTTTCGCAAGCAATTCCGGAATTTTCTGGAAGAGATGGCATTTCGTGATGCCATGCTTGACATGCCATTGATCAGGCATATTTCACTGTTGGATAATGGTGGAGAAAAGATTACCGAGTTGAGGAATCTGGTGTTTGATTTCCCAAGAGAAACTTATTAA

>OR41 partial 540 bp

CATGTTTGTGCCAACTTTAGTGTCATAAGTGACATGATCGAATGTTTAGATACGACCACAGTTGGTGGTTTGGCAAAAATTGTTAAGGAACACCAATACATTTTGAAGCTCGGCGAAGATTTGGAAGATATTTTTACGGCTTCCAATCTGTTTAATGTGCTGGTCGGCTCTTTGGAGATATGTGCGCTGGGATTTAATTTAACGACTGGAAGCTGGGAACAATTTCCAGGATGCCTCCTTTTTCTTCTTTCTGTGCTTCTACAAATCCTAATGATGAGTGTCTTTGGTGAAAACATGATACAAGAGAGCAAAAAGATTGGTGACGCCGCGTTTTTATGCAAATGGTATGAAATGGATGAAAAGTCTAAGAAGACCATTTTGACAATAACGATCAGAGCTAAGAAACCTCAGCAACTGACCGCTTACAAGTTTTCTACGATCAGTTACGGGAGCTTTACAAAGATTATAAGCACGTCGTGGTCATACTTCACTATACTAAAAACTGTATATTCGCCTCCAGAAGTTACACATGTCGACTAA

>AipsIR8a ORF 2442 bp

ATGATAATCGACCTCTCCTGGTCTCCGTGGACAATGGCTGAAGACCTGGCTTCGGAGACAGGTGTACCTCTCGTCAGGACGCTGTTAGGATCTCAACAACTCGTGAAGGCGTTAGATGACCACCTGGAGTCGAGGAATGCTACTGATGCTGCTATTATTTTGGAGAGTGAGAGTGATGTGGACAGAACCCTATACGAGCTGCTAGGAGTTTCCAACATCAGGGTTTGGGTACACGCTGGCCTGACGAGGGATTCCGCCAAAGCTCTGAAGACCATGCGACCTGAACCTAGCTTCTATATCATTGTGGGCGTCAATGGTTTTGTCATGGACACTTATAGAAGGGCTGTAAAAGAGAAGCTAGTCCGTCGTAACTACCGCTGGAACCTAGTCCTGACTGACTACTCCACTCCAGATGTTAGTCAGTTAGTCCTGCCAACGGTGATGCTTCAAGCAGACCAGGTGGAGTGCTGCAAGCCCTTGAAGAGGGAGGACTGTACCTGTCCTTCGGATTTTCAGCGCAAACAATACATTCTGAACGCCCTAATTCAGTACATAGCAGAGACCTACTCGAAGTTGGAGAGTGACTTACCTCTGACCACCAGTACCATCGACTGCGACGACCCTCAGCCTCTGATGAATAGTACAAGAGAGAGGTTGTTTAGGCAGTTCGGGGAAGATGCGGAGATGAGCAATGAGACGATATTCTTTTGGGATGTTGACAGGTCCGGTCTCTTCCTCCGCTCTCGCTTCATCTTATCGACGTACAAGCCTGACGAAGGCCAACAAGCCATAGCCACTTGGTCTGCTGATGACGAGTATAAACTGCTGCCAGGAGTCGAGCTGGAACCGCTCAAGATGTTCTTCAGGATCGGGACTGCTCCGGCTGTACCTTGGACCCTGGTGAAGGTAGACCCAAATACAGGAGAACAGATGTTCGATGAAGATGGCCAGCCATTATATGAAGGGTACTGCGTTGATCTGATTGCTAGATTGTCTGAGACAATGTCCTTTGACTACGAGATCGTTTCTCCAAAGTCCGGTGATTTCGGAAAGAAGTTGCCAAATGGAACATGGGATGGAGTTGTTGGCGATTTGATGAGAGGGGAGACTGACATCGCCATATCAGCGTTGACGATGACGGCTGAGAGGGAGGAGGTCATTGACTTCGTGGCGCCTTACTTTGAACAAACTGGTATTTTGATAGTGATCCGTAAACCAATCAGAAAGACCTCGCTCTTCAAGTTCATGACTGTACTTCGCACTGAAGTGTGGCTGAGTATCGTGGCAGCGTTGGTCCTCACTGGGTTCATGATCTGGTTGCTAGACAAGTATTCACCTTACTCTGCAAGGAATAATCCTGATGCTTATCCTTATCCGTGCAGGGAATTCACACTTAAAGAGAGTTTCTGGTTTGCCCTAACGTCATTCACGCCACAAGGCGGACGGGAAGCGCCTAAAGCGTTGTCTGGAAGGACCCTAGTGGCAGCTTACTGGTTGTTCGTGGTCCTCATGCTGGCTACCTTCACTGCTAATCTGGCCGCATTTCTAACCGTGGAAAGGATGCAGACACCAGTGTCATCCCTCGAGCAGTTGGCTCGTCAGTCCCGGATCAACTACACCGTGGTGGAAGGATCTTCGGTGCATCAGTACTTTATTAATATGAAGTTCGCTGAAGATACGCTTTACAGAGTATGGAAAGAAATAACTCTAAATGCGACATCAGATCAGGCGCAGTACAGGGTGTGGGATTACCCAATCAGAGAACAGTACGGTCACATTTTACTGGCTATCAACGCTTCAGAACCCGTACCCGATGCTAAGACAGGGTTTCAACAAGTGAACGAGCATACAGACGCAGACTTCGCCTTTATTCACGATTCTGCTGAAATTAAATACGAAGTAACAAGAAACTGCAACCTAACAGAAGTAGGCGAGGTCTTCGCGGAGCAGCCGTACGCTATAGCAGTACAACAGGGCTCCAGGTTGCAGGAGGACCTGTCCAGAGCGCTGTTAGAGCTGCAGAAAGAGCGGTTTCTTGAACAGCTGGCTTCGAAATACTGGAACGAATCTGCAAGACAGGCTTGTCCTGATGCTGATGAGTCTGAAGGCATAACCCTGGAGAGTTTGGGTGGAGTCTTCATAGCGACCCTCTTCGGCCTCGGTCTAGCAATGATAACCCTCGCCTGGGAAGTCTTCTACTACAAACGCAAGGAAAAGAACAAAGTCCAAGCATTCAATGCGAAGCCAGAAAAACCTACTTTTGAGACCAAAACGACTTTGGAGACGAAAGTCGCGAATTCCATGGCGAAGTTACGGAGAAGGGGCAAAGTTGGGAAGAAAGGCAATGTTGCGAAAACTGTTACAATTGGGGAGAGTTTTAAACCTGTAGCTGAGAAAGGTGTTTCTTATATAAGTGTGTTTCCTAAAGAGTATAGGCCTTAG

>AipsIR25a ORF 2772 bp

ATGTCTGCGTTCAGTATTTTCTTTTTTCTTTTCTATTTATTTCGGGTCGCTTTTGGTCAAACTACTCAAAATATCAATGTTTTACTGATTAACGAGGAGAGTAATGCTTTGGCTGAAAAGGCTTTTGAAGTAGCTAAAGAATATGTGAGGCGCAACCCGAGCTTGGGTTTGGCTGTGGATCCTGTTATTGTTGTGGGAAACAGAACTGATGCAAAGTCCTTTCTTGAGAATGTTTGCAGAAAGTACAATGACATGCTCTTGGCAAAAAAGACTCCTCACGTTGTTCTTGACTTCACGATGACCGGCGTGGGCTCCGAGACCATTAAATCATTTACAGAAGCTTTGGGCTTGCCAACCATATCCGGGTCTTTTGGACAAGTCGGCGACCTGAGGCAATGGAGGTCTCTGAATGCTAATCAGACAAGGTTCTTACTGCAAGTCATGCCCCCAGCTGATATTCTACCAGAAGCTATAAGAGCTGTCGTCACTAAACAAGATATTACAAATGCTGCTATTATTTTCGATGAATTCTTCGTTATGGACCACAAATACAAGTCTTTGTTACAAAACATACCGACACGTCACGTTATCACGCCCGTTAAGAGTTTTGAGGCAAATGAAATTAAAACCCAACTGGAAAGTCTTCGGAACTTGGATATTGTGAATTTCTTTATCGTTGGAAGTTTGAGAACGATAAAGAATGTTCTAGATGCTGCAGATAAAAATCAATATTTTGGGAGGAAAACTGCGTGGTTCGCTTTGTCATTGGAGAAGGGCGATATTAGTTGCGGTTGTAAAAATGCTACGATCGTGCACATACGGCCGACACCCGACGCCAACAGCAGAGACCGATTGGGAAAGATTAAAACCACATACAGCATGAACGGTGAACCAGAAATCACATCCGCCTTCTACTTCGACCTGTCTTTGAGAACATTTCTAGCGATAAAATCACTGCTGGACTCTGGCAAGTGGCCAAACGACATGAAATATATTACATGCGACGACTACGACGGCAAGAATACACCAAACAGGACCTTGGATCTTAAAACGGCGTTCCAAGAGATAAAAGAGACGCCTACATATGCTCCGTTTTTTATTCCGCAAGATGATCCTATGAATGGGAGAAGTTACATGGAATTCAGCACAGATCTGCTTGCGATCACGGTCAAAGATGGTGCATCTATAAGTAGCCACTCGCTGGGTTCCTGGAAAGCTGGCCTCTCCTCGAACCTAACCTTGACTGACCCCAATAACATGAGTAATTACTCCGCACAGCTAGTGTACAGAATCGTCACAGTCGAGCAAAAGCCATTTATAATACGGGATGACAAAGCGCCTAAAGGCTTCAAAGGTTATTGTATCGACCTCATCGAGGAGATTCGCCTAATCGTTAAATTCGATTACGAAATATCTTTAGCACCGGACGGTAACTTCGGCATAATGGACGAGAACGGAAACTGGAACGGAATTATAAAAGAGCTCGTTGATAAGAAAGCCGACATAGGGCTGTCGTCGTTGTCCGTCATGGCTGAAAGAGAAAATGTCGTCGACTTCACTGTACCGTACTACGATTTGGTCGGGATTACAATCATGATGAAACTGCCGAGAACGCCGACATCCCTGTTTAAATTCCTAACTGTTCTGGAGAATGACGTTTGGCTATCGATTCTGGCTGCTTATTTCTTTACCAGTTTCCTTATGTGGGTATTTGACAAATGGAGTCCATACAGTTATCAGAATAACAGAGAGAAGTACAAAGAGGATGAAGAAAAGAGGGAGTTTACTTTGAAGGAGTGTCTATGGTTTTGTATGACGTCACTGACTCCGCAAGGAGGTGGAGAGGCACCGAAGAACTTGTCGGGACGACTGCTTGCTGCCACCTGGTGGTTATTCGGTTTCATCATAATCGCATCATACACGGCGAATTTGGCAGCTTTCCTGACAGTTTCCCGCCTGGATACGCCTATTGAGTCCCTGGATGACCTGTCAAAACAATACAAGATTCAGTATGCGCCTCTCAACGGATCTGCGGCCATGACATACTTCCAGAGAATGGCTAATATTGAGGAGAAATTTTATGAGATATGGAAAGAAATGAGCCTGAACGACAGTCTAAAAGAGGTGGAACGCGCGAAACTAGCAGTATGGGACTATCCAGTCAGCGACAAATACTCTAAAATGTGGCAAGCAATGGAAGAAGCAGTTCTGCCAAATACCATAGAGGAAGCGATTCAAAGAGTGAGGGATTCCAAGAGTTCCAGTGAAGGTTTCGCCTGGTTGGGAGACGCTACTGATGTGAAGTATCATGTGATGACCAGCTGTGACCTTCAGTCCGTTGGAGACGAGTTCTCGAGAAAGCCGTATGCGATTGCCGTGCAGCAAGGATCTCCTTTGAAAGACCAGTTTAATAATGCAATACTGCAACTTCTCAACAAACGCAAACTGGAGAAGCTCAAAGAGATCTGGTGGAACAACAATCCAGAGGCGATGAAATGCGAGAAACAGGACGACCAGTCTGACGGGATCTCCATCCAGAATATTGGAGGAGTGTTCATCGTAATATTCATGGGGATCGGCCTGGCTTGTGTGACCCTGGGAGTGGAGTACTGGTGGTATAAGTGGAGGAAGAGGCCTATTGTTGGGGATGTTGTTCATGTAACTCAGGTGGAACCAGCAAAATCGTCAAGAACCAACGTAGACAAACAAGGTGAAGGTTTCAACTTTCGCGGAAGAAACTTAGGTCTTAACTTTAACAAACCTAAGTTTTAA

>AipsIR21a partial 2175 bp AAGTACTTATTCACTGATCCGAAAACTTCTAGAAACACACAATATTCACGGGTTGAAAGGAAAAGAGAAGTAAAAAGCAATGATACGTTTGAAAATGAAATCACAAATAATACAATTGAAGAAGTCAAATGGAGGCAATTCAATCAAGAAGAAAATGATGATGATGGAGATATTCATAAAAGAGCGCTAGATCCAGTGTTCCACGGTCATCCAAAAACTAGAGAAGAACTGTGGAATGAGCGTTTTCTAAACCAAACTTTAACTTTTGATCAAACACCATCACTTGTGAATCTGTTACATAACATTTCATTGACTTACTTAAAAGATTGCACACCAGTAATACTTTATGATAATCAAGTGAAATCCAAAGAAAGTTACTTAGTTGAAAATCTACTCAAAGGCTTCCCTATGTCCTATGTTCATGGATATATAACTGATGAAGGGGAGTTAGTACAGCCTGAACTTTTACATGCAAACACAGATTGCCAACATTTCATTCTCTTTTTGACGGAAATCAAATTAAGTGCGAAAATATTGGGCAAGCAACCAGAGAATAAGGTCATTATAATAGCAAGGTCATCGCAATGGGCAGTGCAGGAGTTCTTAGCAAGTGTCAATTCTAGAAACTTTGTCAACTTACTTATAATTGGCCAGAGCTTCAAAGAAGGGGAAGATGCAACTAGGGAATCACCATACATCTTGTATACGCATAGATTATATACAGATGGTCTGGGTGCTAGTCAGCCTGTTGTGTTGAATTCTTGGACACATGGAAAATTTTCGAGAGATGTCCATTTGTTTCCACCTAAAATGACCAAAGGATACGCTGGTCATCGGTTTGTAGTTGCTGCTGCGAATCAGCCACCGTATGTGTTCAGAAGGATAAAGTCAGATCTTGACGGTGGAAATCCTCGAGTGGTATGGGATGGAGTTGAACTACGATTGGTCAAGTTGTTGGCTGAACGAAACAACTTTTCAATAGAAATCATTGAACCTCACGAACCTAATTTGGGGCCTGGAGATTCAGTAGCTAAAGAAGTAAAAATGGGAAGAGCAGACATTGGAATAGCTGGAATTTACTTGACCAATGACAGACTCACAGAAATGGATGTGTCACTACCACATTCTCAAGACTGTGCGGTATTTGTAACTTTGATGTCTACGGCTTTACCTCGCTATCGAGCAATCCTCGGACCTTTCCACTGGCATGTCTGGGTTGCTCTAACCTTCACCTATCTTTTCGGAATGTTTCCGTTAGCTTTCTCGGACAAACATACTCTGAGACATCTATTACATAACAGTGGAGAAATAGAAAATATGTTCTGGTACGTCTTCGGAACTTTTACGAACTGCTTTACGTTCCTCGGTAAGAACTCTTGGAGCAAGACGAATAAGATTACGACGCGGCTGCTGATTGGTTGGTATTGGATTTTCACAATTATAATTACCAGTTGTTACACTGGGTCCATTATAGCTTTTGTGACGTTGCCTGTGTTTCCTGAAACCGTGGATACTATTAAACAGTTATTGGCTGGATTTTATAGAGTTGGAACTTTAGATCGAGGTGGTTGGGAGAAATGGTTCTTAAATTCGTCTGATCCTGATACAGCTAAGTTGTTGAAGAAACTTGAATTCGTACCGAATGTAGAGGCCGGGATAAGGAACACGACCAAGGCTTTCTTTTGGCCATATGCATTTTTAGGATCCAAAGCGGAACTTGAATACATCGTGCAAGCAAACTTTACAGCGACCAAATCAAAGCGAGCAGTCCTCCACATTTCCAACGAATGCTTTGTCCCGTTTGGAATAACTTTGGCTTTTCCAAATAATTCAGTGTATTCTGCGAAACTTAATTTTGATATCAGCAGAATGATTCAAAGCGGACTTATTGACAAGATTACTGATGAAGTAAGATTTGAAATGCAACGAAGTTTGACTGGGAAGTTACTTGCTGCTGGCAGCGGCGTTATCAAAATCCCCTCAGCAGAAGAAAAAGGACTCTCTCTAGAAGACACACAGGGAATGTTTCTGCTCCTAGGAGCTGGCTTCCTCATAGCAGGAACAGCACTGATCTCAGAATGGATGGGAGGCTTCACAAGAAGATGCAGATTCACAAGAAGAATTGATACACCAATCAGTATTAATTCTAGAGAACACTTAATTCCAACACCAAGA>AipsIR41a partial 975 bp

GAGTGGCGTGTGATGGACTTTTCAGTAGCTGGTGTTAGAACTGCTATCACTTGCATAGCCCCAGCACCTAGATTATTGTCAAGTTGGGAGATGCCTTTGATGCCGTTCACTTGGTATATGTGGATTGCTGTAGCATTTACCTATATTTATGCGTCAACCGCAATTTTAACAGCACAAGGGTGTTCTACTACTTCGTATCCTTTTTTAAAAACCTTTGGAATGATGATCGGACAATCTCAATACCAGAACACTGAAAGTCATTCATGGAAAATGCGAAGTGTAACCGGTTGGTTGTTGATAGCTGGATTGATTCTGAGCAGTGCTTATGGAGCTGGTCTTGCTTCCACGTTTACTGTGCCCAGATACGAATCCTCTATCGATACAGCACAGGATATAGTAGATAGAGAAATGGAATGGGGCGCTACACATGATGCTTGGATATTTTCCCTTACTTTGTCCTCTGAGCCACTAGTGAAACAGTTAGTGGGACAATTTCGTATTTATTCTTTTGATGTACTGAAAGAAAAAAGTTTTACACGAAGTATGGCGTACAGTATTGAGAAGTTACCAGCAGGTAACTTCGCAATAGGCGAGTACGTAACGCAAGAAGCTGTCCTGGACATGATGATAATGCTGGAAGACTTCTACTACGAGCAGTGTGTCCTGATGATGAGGAAGAGCTCTCCATACACGGAGAAAGTCAGCCAACTGGTGGGACGCTTGCATCAGTCCGGACTTTTGCTCGCTTGGGAAACTCAGGTTGCGCTAAAGCACCTAAATTATAAAGTACAAGTAGAAGTGAGACTATCGAGAAACAAGAACGATGTCGGTAGCACGGAACCCTTGAATCTTAACAATATTGTGGGTGTCTTCATTGTATACGCAATCGGACTTATAGTATCCACAGCATTTTTTGTGGGTGAATTGTATGTGTATCACCGTAGAACGAAGAACAAGTTGGTGCAATTTGAATAA

>AipsIR75q.1 partial 1464 bp

TTCGGAAGTTATAATAGCTCAAGAAAAGGAAATAATAATATCTTGTTACATACCATATACAAATTGAGGGCCAACAATGAATGGAATACAGAATACTACGGCACTTGGTCGGTTGACTATGGTCTGAATAAATCAGAAGATAGAATGCGCTCAAATATGCTAAGACGAAAAGATTTCAAAGGAGAACCATTGACAATGTCTGTTGCTATTGGCGATAACAGAACTAAAACTGATTTACTTGGACTCAGCAATATCCTCGTAGACACACTTGCAAAGAGCAGTTTCCGTTTCATCGATCCCCTGTATGACTTCTTAAATGCGTCCAGAGTAGTAATATTCGCTGATACTTGGGGATACTTAACTGATGGCACTTGGAATGGAATGATCGGAGACATTAAAAATGGGAAAGCGGAATTGTGCGGAATCGTGACGTATATAAGTATAGAACGAATGGCCATTTTGGAATACTTGACAATCCCGACACCAATAACAGCAAAGTTTGTATTCAGACAACCTCCTCTATCGTATCAAACTAATTTGTTTATCTTACCTTTCTCAACTAGTGTGTGGATGTGTGCCGGAGCATTTGTTCTAATACTGGGTGCCATACTGTATATCAACACGAAATGGGACAATAAAAAATATGAAAAGTATAACAAGCAAAAGATGGACCAAACATGCTTGCCACCAACTTGGGGCGACATCACAATTTTTGTTCTTAGTGCAATATCTCAACAAGGAAGCTCTAATGAGTTAAAAGGGACTCTTGGACGTCTTGTAATGTTCATTGTGTTTTTGGCTTTCCTTTTTCTATACACGTCATACTCGGCCTATATCGTAGCGTTACTACAGTCGACATCCAACCGCATCCGAACACTCACAGATTTGCTGAACTCAAAACTAGAATTGGGAGTTGAAGACGTACCTTACAACAGATATTATTTCTCAGCCGCTTATACTTCCAAAGATCCGATCAAAAAGGCAATTTTCGAAACCAAAGTAGCTCCTAGAGGTAAACCAAACTTCATGAGCATTGAAGAAGGAGTCAAAACTATGCAAAAGAGACCATTTGCCTTCCATATGAACATAGGCACTGGTTACAGAGTCGTTTCAGCGTTCTTTCAGGAGCATGAAAAATGTGGCCTTCAAGAAATCGATTTTATTCAAAACAACAAACCGTGGCTTTGTAGTCGGAAGTACTCTCCGTTCGGTGAAATGTTTAAAATTGGGTATATCAGGATCCAAGAGCATGGTTTGACTGACAGGGAAAACCGTTTAATTTACGCTAAGAAACCGGTTTGTTCAGTGATGGGTGGGAGTTTCGACTCAGTCAAGATGGTGGATTTTTACCCCGTGTGTCTTATGCTGTTGTATGGGATGATTCTGGCATTTGTCCTCCTTGGAGTCGAGATTTTGGTGCATCGTCGTCAGCAAAAGAGAGAAAGCAGTCGAAACAACATTTAA

>AipsIR75q.2 ORF 1881 bp

ATGCTTGTAACCACGAAGATATTTTTTACTGTTTTACTTTTTACCTTAAGCAATGGGCAAGACAGACAAGCAGCCATGATTGTTGATGTAATTCGATCCGTCGGTAGACCATCTTCGGTGATTGCTAAGCTTTGTTGGACCTCTACAAAAATCATTCAACTACACTCTCTTCTTACGAAAGAATACATTCAATTCAGTGCCGGAGACGTCATCAACGGTGATAACGCTCAGTTTTATGATGAAGAGCAACATGTCGTGTTCTTAGCTGATCTGCACTGCCCTGATATTGATGCATACTTTCAAAAGAACAGCTTTAGAAACTTTTTCCGAGCACCGTTTCGATGGATTCTTTTCGGAGACCCTGACAAAGGCGACATTGTACCAAAAACTGTTGCTAACATAGACGTGCTCGTCGACTCTGAAGTGCTGGTACTATGGAGCGTGGACGATACCTACGAAATGCATTATATTTACAAAGTCGGCTCCAACACAACTTGGAACACTGAATATTATGGGACTTGGGATACAAAAAACAGGTTCCAAAAATCTCCAAGATTTATTGAACCGACATCTTTGCGACGTCTCGATACTGACGGATACAAAATAAGTATCTGTTATGTACTAACAAATAACAAGAGCGTCGATCATTTAAGTGATGGCCTAGACGATCACATTGATACGATTACGAAGGTGAGTTTCCCCACGACCAACCATTTGCTGGACTTCCTTAATGCTAAAAGGAAATATGTTTTTGCTGACACTTGGGGTTATCGGGTCAATGGCACTTGGAATGGAATGACTGGCTACCTCGTTAGGGGTGAAGTTGAGGTTGGAGGATCACCAATGTTCTTCACATTTGAACGTGTATCAATAGTAGACTACATCTCAAGTCCGACTCCAACGCGTTCAAAATTCGTATTTCAACAACCAAAACTATCTTATGAGAACAATTTGTTCTTACTACCGTTTAATACTACAGTCTGGTACTGCACGATAGCTTTGATATTCATAATATATCTTGTGCTGCTAATGGTTACGAGATGGGAGTGGAAGAAAACCAGTCACATGATGGAATCGAGGGAAAAAGATTCTGGCGTTTTAAGGGCCAATGTCGTTGATGTTATCATTTTAATATTTGGCGCAGCGTGTCAACAAGGAAGTCCTTCTGAGCTGAAAGGATCGTTGGGTCGAGTGGTAATGCTGGTACTATTCTTAGCGCTGATGTTTCTGTACACCTCCTACTCAGCCAACATTGTAGCTCTTCTCCAATCTAGTTCATCACACATTAAGACTCTGGAAGACTTACTACATTCTAGGATCAAGTTTGGAGTTCATGATACTGTTTTCAACAGATATTATTTTTCGACTGCTACTGAACCAGTAAGAAAAGCAATTTATGAGAAGAAAGTGGCTCCACCAGGCACGACTCCTCGGTTCATGACCATGGATGAAGGGGTTAAACAGATGCGAAAGGGTCTATTTGCATTCCACATGGAAACTGGAGTTGGCTACAAGTTCGTTGGAAAATATTTCAATGAAGGGGAAAAGTGTGGGCTTCGAGAAATACAATATCTGCAAGTAATAGATCCATGGTTAGCTGTGAGAAAGAACACGCCATTTCGGGAAATGTTCAAAATTGGAACCAAACGTATTCAAGAACACGGACTGCAATATCGGGAAAATCGCCTCATGTACGAGAAACGCCCGAAATGTTCAGGAGGAGGATCCAACTTTGTATCAGTCAGCATGGTTGATTGTTATCCAGCTGTACTCATACTAACATACGGAGCTATCATTGCTTTGTTCTTACTTGGTCTAGAAATATTAGTATTTAAAAGAGAAAAGCTCATGCACTGCATGAAACACAAAAATGACCATTAA

>AipsIR75p partial 492 bp

AAGGGGAAAAAGCACTTCTATGATCTCAATGAAGGAGTGGAGCGAATCAGACAGGGTCTTTTCGCCTTCCACTCGATCGTGGAGCCAGTGTACCTGCGCATCGAGCAGACGTTCCTGGAGACGGAGAAGTGTGACCTGATGGAGGTGGACTATCTGAACAGCTTCGATGCCTTTGTGCCTGTGAGGAAGGATTCGCCTTATTTGGAGATACTGAGAGTTGCATTCAAACAAATCCGTGAGTCCGGTATCCAGTCAGCTGTTTCAAAGCGCTTACAAGTTCCAAAGCCGCACTGTACGACCAAGATGTCTTCATTCAGCAGCGTGGGCCTGATGGACATGAAGCCAGTTCTGATCTTCATGATGTATGGCGTCTGCTTGTCTGTGGCCATCGCTGTGGCGGAGATCGTTGTGTTTAAGCTGAATTCGCACAGAAAAAGAGCGAAACCCTCCAAAATAGAGATTACGGATTTAGATGTATCAAACTCAGCTTAA

>AipsIR76b ORF 1629 bp

ATGGCCGGCATAGAACTAATCATATCGTCGATATGTAACGCCACGTTTTGCGAAGTACCTTACAATGATACCTATAAAGGACCGGACGCGTCACAAGCAAAGGAGATCAATTTTATGAATTTGGCGAAGGAAGTCAATGGGAAGAATCTTAAAGTAACGACGTATAATAACACCCCATTGAGTTGGACTGAATTTCACAATGGCACGGTTGTCGGCAAAGGCGTGGCTTTTATCATCATGGATATTTTGCGGAAAAAGTTTAATTTCACCTATGACGTGGTTGAGCCTAAAAGAAACTATGAGATGGGCAATAAGATGAATGATGACTCTATAATAGGACTCCTTAATACCAGTAGAGTGGACTTAGCCGCAGCATTTCTACCAACATTGATAGCTTATCGGGAGAGAGTGTCATTCTCAATAGACCTGGACGAAGGCATATGGGTGATGATGCTAAAGAGACCTAAGGAATCTGCCGCCGGATCTGGTCTGCTCGCGCCTTTCAATGAACTTGTGTGGTACCTAGTCCTGGCAGCAGTTCTAACATTCGGGCCCTGCATCACTTTCTTCACGCGGGTACGAAGCAAGCTGATTACGGATGACGAAGGTGTTCTACCGTTAAAGCCTAGCTTTTGGTTCGTTTACAGCGCTTTCCTCAAGCAAGGCACCAATTTAGCACCTGAAGCAAACACAACCCGCGTCCTCTTCGTGACCTGGTGGCTGTTCATGATACTACTCTCAGCGTTCTACACGGCCAACCTGACTGCCTTCCTCACGCTGTCCAAGTTTACCCTGGCCATCGAGTATCCACGAGACTTGTACTCGAAGAACTACCGCTGGGTTGCTTCAGCTGGCAGTTCGGTTGAACACGTTGTTAAGTCGGATGGCGAAGAACTATATTACCTGAGCGCAATGATCAGCAACGGTAAAGCAAGGTTCCTATCAGTGACAAGCGACAAAGACTTCTTGGAAGCTGTGAAGAAAGGCGCAGTACTAGTCAAGGAACAGACCGTGGTAGACCATCTTATGTACAACGATTATACTTCGAAGAAAGACGTAGAGGAGTCTGATAAGTGCACCTATGTCGTGGCTCCTAATGCCTTTATGAAGAAGCAGAGGTCTTTTGCTTATCCTGTTGGCAGCAAGTTGAAGGCGCTATTTGATCCTGTACTAACCCAGATATTCCAATCGGGCATTCTGGACTTCCTGAAGCGATCTGACCTGCCGAGCACCAAGATCTGCCCGCTAGACTTGCAGTCCAAAGATCGGAAGTTGAGGAACAGTGATCTTATCATGACGTACCTCGTGATGGTCACCGGATCTGGTGTTGCTGTCGCTGTATTTGCTGCTGAGATATTCATAAAACGTTACATCTCTGGAAAAATAAGCACAGACAAAAAAGGAAAGAGAAAGAAGTCTAAAATTGGTAAGAAATCGACGAAGTACGACGACAGCCGACCTCCACCATACGATTCACTGTTCGGCAAGAATCCTCGGTTTAACGTCGAGACTACACGCACGAAAATTATAAACGGTAGAGAGTATTATGTATACGAGACGGCAAATGGCGAAAAGAAACTTATCCCTGCTAGAGCTCCTTCTTCGTTCCTCTATAGATCAGATAAATAA

>AipsIR87a partial 534 bp

ATACTGTCTGGAATTCCGCTGGGAGGTAGGGAGATTTATCGGTCTTATTTTGAGACTAATAACGCGAGTTCCTTTTATTTATATCGCAAGTACAATTCGACTACGTTTTCGGAGGGTGTAAGACGGGCAGCTTTGCAGAGAAACTTTGCCGTCGTTTCTTCGAGACGTCAAGCCATCTACCAAGATCAGAAATTGGGTAAAGGGGCGCCATTAATTTATTGTTTTCCTGAAAGTAATAATATGTATAAGTATGGAGTTGCTATTCTGACGAGGCGGTGGTTTCCGATGTTGGACAGATTCAATAATATAATTCGGAGTGTGTCAGAGAATGGTTTGATAGATAAATGGATGAATGAGCTCTTGATACATTCGGGAAATTCTGAAGAATCTAGTACTATTGAGCCTTTGAGCATCCAGAACTTACTGGGTGCCTTTATGTTCATCGGTTTCATGTATGCGGCTAGTATCGTTATTTTCTTGGGAGAACTTGCTATGGGTGTTTTAGAAAAAAGAGACAAAGTAAGAAGGATGTGA

>AipsIR93a partial 282 bp

CAAAAACTTTACAATAAAGTAATATTAGCGTCTGTTTTGAAGAAAGAATTGGCAAATGACACGATTGCGGATACAATGCATGGCATGGATGCTAAGCTGACGATGATAGCGATTTCCAAAGGACAAGGCGCTTTGGGAGCGGCATCTTTCACTGTTCTATCTGATCCTATGCCAGGTATAAACTACACTATGCCAGTGAGTATACAGTCGTATGCGTTCATGATAGCGCGACCAAGAGAGCTCAGCAGAGCTCTGCTGTTTCTACTTCCATTCACTACCGAC

>AipsIR1 partial 897 bp

GGCATCGCCTGCACCCTGGTCCTCACCGTCGCAGCTCGCCTGGAGAACAGACCCAAGTCCGGCTGGTATGCCTTCTTCAGCGTGTTTGCTGCTATATGCCAACAAGATTTTGAAGACGGAGTTCAACTTTTGGAAGAGACATTTTCGAGCCAAGGGCGTAAGGCGACTCTTCTGGTAATCGGCCTGACAAGTATGCTGCTGTATAACTACTATACCAGTAGCGTGGTGTCCTGGCTCCTGAATGCTGCAGCTCCGTCTATTGCTACCCTTGATGGCCTCATCAGTAGTGACTTTGAACTGATATTTGAAGATATTGGCTACACTAGGGGATGGCTTGACAACCCTGGATTCTTTTACTACAGTGGATTCACGAACTTGAAAGAAGATGAATTGAGAGAGAAGAAAGTGACTAAGGCGAAGCGCTCCGCAGCAGTGTTGCAGACTGTGAGAACAGGTGTCGAACTCATGCGGACTGGAAAATATGCTTTTCACACGGAGCCGTACACAGCGAGCCAGGTGATCTCCAAGACCTTCGAAGACGCTGAGCTGTGTAACCTGGGAGCTCTGCAGATGATGTTGCCAGCTCACGTCTACATCATGGCGCAGAAGAGGAGTCCTTATAAGGAGTTCTTTGATTGGAGTCTCCTGCGCCTCACAGAGCGTGGCCACGTGAAAGCCATTCGAGCTCGCTTCGCTGGCACCATGCCAGCCTGCTCGGGCGCACAGCCGCGGGCCCTGGCTCTGGGTCAGGCAGCTCCTGCCTTCCTCACACTGTTCCTCTTTGCTGTGCTAGCCTACATCATATTAGCTTTTGAGATCCTCTGGCAAAGAGTCCAGCTGAAAAAGCGGGCAGTGAGCGAGGCGCAAAAACTGCCGTCACCAGTGATCGGACTATGA

>AipsIR2 ORF 1452 bp

ATGAGTTACGTTTTGATGTCACTAGATGCCCACACTTTGGATCTGGAAGAATTGCGCTATGGATTATCGAACGTAACTTGCTTGAGGATTTTCGACCACTCAGACTCAAGAGCTAGGACGTATTTAGCAGATTGGAAAGTCAGAGCTTCTGCCGATGTGAAAATTCCAGGACAATCTTATGAAATAACTGTGGAAACTGCACTTGCAAGTGATGCAGCCAGGCTTATAACGGATGCCGTCGAAAGTGCTCCCGAAGAATTTAAAATAGAAGCCCAAGAACTTAGTTGTGATTCTGAAGACACATGGGAAGTCGGTGAAGATTTCATCAATCATTTGCTAACGAATCCAATACCTGGCATTACAAATGAAATCAAATTAGACAATATAACTGGCGAGCGATTGAATTTTAACGTTGAAATAATGGAATTATCGAACAGTGGATTTAATAGTATTGCAAAGTGGAGTCCAGAAGCTGGTTTTGAATATGTTCGAACCGCGGATGAAACGTCAAACCGTTCGGCAGAAAAATGGCAAAACAAAACGTTCAAGGTTGTGTCACGCATTGGCGCACCGTACTTAGTGGATGTAACCCCTAAGGCTGGAGAAACGCTAACCGGCAACGATCGATACGAAGGATATTCAAAAGACTTAATCCACGAAATATTAAAAGAAATACTTCACTTAAACTATGAAATCGAAATAGTGCCTGGTAATGGCTACGGGTCATACAACAAAGACACAAAAAAATGGGATGGTCTAGTGGGACATCTTTTAGAAAGGAAAGCTGATTTAGCCATTTGTGGTTTGACCATAACTTACGAACGTCGCTCTTCAGTGGATTTCACAACACCTTTTATGACTTTGGGTATAAGTATACTTTATTTAAAGGCAACTCCACCCGATCCGGAGCTGTTTTCATTTCTTAAACCATTTTCTGTTGATGTATGGATCTATATGGCGGCAGCTTATTTAATGGTGTCGTTATTGCTTCATATTTTGGCTAGACTGGCACCAAATGACTGGGAGAATCCACATCCTTGTGACAAATCACCTGAGGAATTGGAGAATATTTGGCACATTAAGAACTCTTGTTGGCTTACAATGGGTTCAATTATGACACAAGGTTCTGATATTTTACCAAAAGGTTATTCTACTAGATGGGTCTGTGGCATGTGGTGGTTCTTCGCCTTGATCATGTGCTCCTCCTACACTGCCAATCTGGCCGCTTTCCTCACCAATGCTGCAATGGATGACTCGATCAAATCTGCCGAAGATCTAGCTATGCAGACTAAAATTAAATATGGAACACTGAAAGGCGGGTTCACGTACTCTTTTTTCAAGAGATCCAATGTGTCTATGTACCAAAGAATTTTCGGTGCCATGGAATCAGCTCGACCATCAGTTTACGTCTTGAATAACGACGAAAGTTTAGACAGAGTGCTCAAAGGGTAA

>AipsIR3 partial 876 bp

GATACTGTAATCAATACGATCTTGTTTGATAAGGACCGCAATACGACCCTAATTGAAGTACTTGATAAACTGAATATAAGTGTCGATGCTGATATTGTGATGGCTAGAACAGATAACTCCAAATACACTCTCTACGATGTATACAACTATGGAAAGATCCAGGGTGGCAATTTGATCCTCCATGAGGTTGGGACTTGGGACCACTATAATGGCCTCCATTTTGATATTAATTTGAATGGCTACAAATACTATAGAAGATGGGACTTTCAGAATATAACAATGAAGATGGTTTTGGTTGTACAACCAGCCCCTAAGCACTTCGACCCGGAATTGTTAACTGGTCTAGAAACTGTTCCTGGAGTGTCGATGATAACTCAAACATCAGCTGCAGTTTTGTACGTTGTCGCTAAAATGCATAACATCAGGTACACTCCCACCATTATGGATAGATGGATTGGTACATACGAAAGAAATAGCTCTAGAGTGGTGTCCAATAGTTTATATTTTCGAGAACAAGATCTGTCACCAGTAATTCGTCTTCTCAAGTCCGTTCAAGAAAACAATGATATATTGATTTCACCGCTGACTGCCATCGAAACTCGCTACTACTACCGCATACCAACGGTAGGTCCTGGAAAGTTCGAGAACCAGTTCTTGCGACCCTTGAGTCCTACAGCATGGTGGTCTGTGATAGCTGTCTCTGGACTATGTGCTGCTCTCCTGCTGCTATCAGCATTGTTGGAACAACGTCCATCGTCGGTGCAGTATGCTGTGTTCTCCGTTGTAGCTTCAATTTGCCAGCAGTTTTTCCAAGATATTGATGATGGAGCAACTAAGAGAATTTCAACAGCTCGCAAGGTCACAATCCTGGTGACT

>AipsIR4 partial 1317 bp

GAGGATACTGAGGAGACTAGACTTGAAGAGATATCCTTTGAGAATTGCCACCCAGTAGGACATTATTCCAGTGACAAATATGATGGAACATTTGTGGATTTTCTGGAGGACGAGTCTATGTCAGACCAGGACCCTGGCATCCGCAGTGGCTACGGAACTGCTATGCTCCTCACTGAAGTTGTAAATGCACAAGACGTTCTCATAGAAAACGAGTTATGGGCAGCAGAGATCAACAACAACAGTATGTACGTAATGGTTTCCACCGGTGAGGCGGACATCTCAGGAGCTATACTGCGCATTTTGTACGAGAGAACATTTACTCTTGACTATGTCATGCCGATATGGCCGTTCAGGGTAGGCTTCACATACTTAGCGGAGCGCGAGAGTAGCAGCAACATGTATTTGGAGCCATTCTCACCAGCTGTGTGGTGGTCCTGCCTGGCCATGATGGTCATCCTGGCAATGGTCGAGTGGATAACCGCTAAGTCACCCAAAGAGAAGGATGGGGCCTTGTATACTGTATTGACTACCTGGTTGCAGCAAGACGCCAGCGCAGTGCCCGAAGGTGGATCAGGTCGCTTTGCGTTTACGGTTCTATCAGTAAGTGCAATGTTGGTGCACGCGTACTACACGTCTGCCATCGTTTCTGCACTCATGAGCACCGGACGCGGTGGACCTGACTCCCTGAAGGCGCTCGGAGACTCGAAGTATGCTATCGGCTCGGAGGATTACGATTACATGCGTTATTTGTTCTTTGATGTGAAAACCACATGGGACGACCTAGAATACTTGAAGAAGAAGAAGATGACGTCCAACTTCTACCAGGAGCTGGAGCGGGGCGTGGAGTTGATCAGGCAGGGCAGCCACGCCTTCCACAGCGAGTACAACCAGATATACCCGCACTTTAAGACCTTCAGCGACGACCAGATCTGCAAGCTGCAGCATGTTGATACCATTCCTGAGGTTAATACCTGGATACAATCAACTAAGGACGGGCAGTGGACGGAAATCCTACGCACAGCCGGAGGATGGTTGCTCGAGACTGGTCTAGGGAAGCGTCTGGTTGCTCGCTTGCGAGTTCCGCAACCACCTTGCAGAGCTTCGCTACTCGCTGAGAGGGTTAAACTGGGAGATATTGCTCCGTTACTAGCCCTGACTGTGTTTGGAGGCTTCTTGTCAGTAGTCTTGCTTGGTGTGGAGATCCTGGTTGCTAAAACAAGAAGTAAAAGGTTGCGGGAGGGTGATACTGAAGGAGAAGAAGATGTTGAGGATGTATCTGAAGGTGTTGAGTATGATGTTAACAATGTAGACCCGTGA

>AipsIR5 partial 342 bp

GATGACGACGCGGGCGGCGGTGAGGCCCAGCCCTTAGTCCTCGCGAACGTGGGCGGAGTATTCATTGTGCTCGCCGCCGGATCAGGCATGGCCGTTGTCTGTGCCTTCTTTGAAATGGTGTTCGACGTATGGATGATATCCCGCAAGATGAAGAATTGCATGTCCAGTGTGACCCTTCACCAAAATATAGTCGCTTCGAGTCATTCTCCGCAGTTGTTATTGTTAAAATTGGTATCATTCAGAGAAGAACTGATGGCTGAATTGAAATTCATACTAAGCTTCAGTGGTGACACCAAACCGGTTCGTCATAGAGAATCTACTGGCAGTGGATCAGGTGGATCT

>AipsIR6 partial 972 bp

ACTCGAAAGACCAACCCAATTACAACACCATGTATCAAAATCAAGGCGGTTATCCTCAGCAAGGAGGTTATCCTCCTCCCGGGGGCTACCCTCCTCCACAGGGAGGATATCCTCAAGGAGGCTATCCCCCGCCGGGGGGATATCCCCCTCCGGGTGGGTACCCCCCACCGGGCCGGATACCCCCCACCAGGGGGATACCCTGCACCGCCAACGTACGGTGGCGCGGGATATGGCGAGCCTCAAGGGTATGCAGCTCCCCCAGGAGTGGGCGTGGGCGAGGATGGCGACGTCAAAGGATTCGACTTCAACGAACAGTCCATCAGGAGGGCTTTCATCCGCAAGGTGTATGCCATCCTCATGTGTCAACTGCTGGTCACGATGTCCTTCATTGCTCTGTTCCTGTTCCACGCACCCACTAAGCAGTGGGCCTACCGGAACACCTGGCTTTTCTGGGTCGCATTCGCCGTGGTATTCGTATGTCTGATCGCGATGGCGTGTTGTCCGAACGTGCGGCGGCAGGCTCCTATGAACTTCATCTTCCTGGGGATCTTCACGGTGGCCGAGAGCTTCCTGCTCGGCGTCACCTCCAGTGTCTATGATGTTGATGCGGTGTTGATGGCAGTCGGTATAACGGCAGCAGTGTGTCTCGCGCTGACGCTGTTCGCCTTCCAGACGAAATGGGACTTTACGGTCATGGGCGGCTTCTTACTCTGTGCTACCGTCGTGCTCCTTGTCTTTGGTATCGTCTGCATATTCATACCCCACAACAAGATCGTGACCCTCGTCTACGCTTCAATCGGAGCTTTAATCTTCTCGCTGTACCTCGTGTACGACACACAGCTCATGATGGGAGGCAAGCACAAGTACAGCATCTCGCCAGAGGAGTACATCTTCGCAGCTCTGAACCTCTACCTGGACATCATCAACATCTTCTTGTACATTCTCACCATTATTGGAGCTGCTAGAGAATAG

>AipsIR7 partial 615 bp

CTGTTGTTTCAGGCTTCCAGAAACAAATTGTTCGATGCGATGCACCCCTGGCTTGTTTTGTCTGATACTGATGATCCTGATAACAACACCCGTTATATTGATGATACATTTCAACATCTAAACTTGAGTGTGGACGCTGATATCACCGTAATGGCCACAGATGGTGATAACTACACAATGACAGACGTGTACAATTTTGGTAAAATACAAGGAAACCAGTTAGAGACAGCTCCTCTTGGAACCTGGCGACCTGACGAAGGCTTAGATATTCACCTTAAAGGGTACAAGTATTACAATCGTTGGAACTTTCACAATTTGACATTGCGAGCCATCACTGTGATCGTGGAACAACCAGAAGTCTTCTACCCAGAGATGTTGTCAGAGATGGCGTACACGGCGGGAGTGGCCGCCATGACCAAGATCACATCACAGATGCTCAACACGATCAAAGAACAACACAACTTCAGATTCAACTACAGCATTGCAGGTCGTTGGATTGGAGCTCCGGAGAGGAATACTACTCTGGCTGTAACGAATTCTTTGCTTTGGGAGGAGACGGACCTATCCAGCACTTGTGCCAGGATCTTCCCCAACTGGCTCGATTGGGTCGACATA

>AipsIR8 partial 330 bp

GAGTCAGCATACTGCATAACGATTATTTCGGATGTACCTTTGACATTCTCCTGGTCCAAAAACTTTATTTCTTTAACCCCAAATAATGAAAATTTGGTAGAACAGATTTACAATGTGTCTGAAATGGGGTGTTCCGATTACATAGTGCGTATGCAACACCCGCAAAATTTTATGACAGCTTTTGAAACAGTTGTTCATACAGCAAAAGTAAGGAGAAGTGATAGGAAGATTATATTCTTACCGTATGATGAAGAGTACAACGAAGGTTTTGATATGGACCTGCCTTCACTGGTGTTTTCGATGAAAGAAAGCAGTTATGTTGCGAATATA

>AipsIR9 partial 307 bp

TTCGAGGGTTACGCCATTGATCTCATTCACGAGATATCTAAAATTCTGGGCTTCAATTATACATTCAAGCTGGCGCCTGACGGTCGATACGGGTCCTACAACCGGGAGACCAAAGAGTGGGACGGCATGATAAGAGAATTGTTGGAACAACGAGCCGACCTCGCCATCGCTGATCTCACCATTACATACGACAGAGAGCAAGTGGTTGACTTCACAATGCCGTTCATGAATCTCGGCATTTCCGTGCTTTACCGCAAGCCCATTAAGCAACCACCGAACTTGTTCTCCTTCCTATCGCCGCTCTCGC

>AipsIR10 partial 714 bp

ATGAATATAATATGCAATATTTTACTTACTCTTTCTGTAACAGACGTACCCTTTATTATTGACATTCTAAAACATAAAAATATAAAAAGCGGAGTAATATTTCATTGTTATGACAACTATTTCGTGAACGCGATGCATAAAATATTCAACGATCACGATATCCTGATCGCGAGTTCTAAAATTGACTATAATGGTACTTATGACGTCCCGAAGTCCTATCCCAGAGTTGGAATGGTGATAGACACTGCTTGTGGAGGCTGGACCTCGGTGTTGGACATGAGTACGTCGCCCTTTCAAGGATATTCCTATATAATAATCACGGAAAATCTGTCATTAACTACGGAAACTTTATCTCGGTACCCAGTAGAGGTCGACTCAGATGTTATCGTCGCACACAGGATGAATGGAACATTCATTTTATATGAGGTCTATAACACTGGATCTAATTTCAAGGGGAAATTCAATGTAAGATTAGTAGGAATCTGGGATACTTCATTATGTATCAAAGACTCAAAAAGATGGGACTTACAAGGCGCGTTTGTAAAGACGGTGGTTGTTATACTCCCGTCGCCCTGGCTGGCCAACCAGACTGTGGAGCAGTACATGGAGAAGCCTATTAAGTCCCAGATTGAGGTCGACACTGTGCATCGAATGAAATTCTTCACTATATTGAAGTTTATGCGAGATATGTATAATATCAGCTACGACTTGCAC

>AipsIR11 partial 255 bp

GCCATCCTGCAGACGCCCAGCGACGCGCTGCGTACCATCGACGACCTCACGCGCTCGCCCATGCTCATCGGCGTGCAGGACACCACCTACAAGAAGGTCTACTTCCTCGAGAGTCCGGACGAGTCGACACAGCAGCTGTACCGTCGCAAGATCCTGCCGCAGGGCGAGCGCGCCTACCTTAGCGAGGTCGAAGGCATCGCGCGCGTTCGCACCGGCTTCTTCGCATTCCAGGTGGAAAGGAGTTCAGGGTACGAC

>AipsIR12 partial 723 bp

GGCGTCTGGAGTACCTATCCAGAAGACGCGGGTTCGAATCCCACTTGCAATCTGAAATTTTTCTTTCCTCAAAAATCATTTAGACATACATCCATCCATTCGCTCAAACTTTCACGTTTTGAAAATAGATATTTATTCTTAGTTAATTTTATTTCTTTTTTGTTTGAGCTGTACATCATGGAGGGCCCGTCAACAAAGATACCACACCCACCGCCAATGGATTCGGGGTCGAAGCATAACTATTTTCAAATCAACAACCGCAACATCAGTGTAGTCCTGGGCAAACTGGCGCATCTTAAATCAGTCACTGTTGCGGATGGATTGGACGCCGTTAAGAAGGGGCACGTAGCTATACTGTCTGACTATACTACCATCTATCCGTACATAAAGAGCACATACGATAACGATGACATCTGTAAATTGGTTTCGATCCACTTGGCCTCGCGAATCAAGAAATATTTCTATACTTCGAAAGATTTCAAGTACAAGGAGCAATTTAAAGTTGGTACGCTCCGGATCAAAGAAGTGGGCATACTAAACCGTATAGTATCAACAAATTTCGAAGAGCCAAAATGCGAGAAATCACATCTCGTACAAATACGTTTGTCGCAGATCTCGATACCACTCACTATGCTCGCTTGTGTTTACGTCGTTTCATTATTGATATTAGTCGCTGAGAGAATTCATTACAAAAGGAATGTGGTCTGGCCTTATGTGGATTAA

>AipsIR13 partial 1134 bp

TTCATACATTTTACTTCAACCATGAGTGAAAACCCACTACTAATGACAACTGGAAATTCTGGACAAATAACAAAAACCGCTGAATGCGTTTTAAAACTCTCCGCGAAATATTTCGTAGAGAAAAAGGCTCTAAGTGGCAGCATCGTGATAATCAATATCAACTCTTATAAATCCACTACACAAGGACTATTATTGCAAACTGTACACAGTGGTATCAAATATTCTATTATGGTAAAAGATTCTTTTTACCCGCATGCAAACGCTTCACATTTCCCTGAGAAAGCGAAAAATTATATGTTGATTTTGGAAGAGAAATCTGAACTCGAGAGAAATATATTGCAATTGAACAAATTGCCAACTTGGAATCCTCTCGCAAAAGCTATAGTGTTTTATCAGTTGAAATATAACGACACCGCTGAGGAGACGGCGACGGAATTCATTAACGAACTTCGAGATTACAAGTTGTTTAGAACAATCGTGTTCATTTACGACGAATATAATGATGTGGTTATATCTTACACTTGGAAACCTTACAGTGACACTAATTGTGGAGGAAAATGTGACTCCGTATACATACTAGATCGTTGTACGAATAATACAATTTACGAATTCCAAAAACAGCACGAGTTATTCCCTTCAAACATGAAAGGCTGTCCCTTGGTGGCTTACGCTGTCATAGCTGAGCCATACGTAATGCCTCCAGTAAGTAAGCTTACTAATACTTCGTTTGAAGACGCCCACGTGTTTCCAAAGGGCGGTGAAATAAACCTTGTCAAAATTATAAGCCAGTTCACAAATATGTCACTAATAACTAGAACATCGGAGATTCAAGAGAATTGGGGCCAGGTGTTCCAAAATGGAACGGCTACGGGAGCTTTTGAGGTGTTGAGGAATGAGTCAGCGGACCTGGTCATAGGAAATGTAGAAGTGACGAGAATACTTCGAAAATGGTTTCATCCAACTGTAAACTATCTACAAGATGAGATGACTTTTTGTGTACCGAGAGCTGGCCAAGCGCCAACCTGGGACAACCTGGTCATTATATTCCAGTGGACTACATGGGTGGCCTACACTCTTCAGCTTCATCATAATGGGTCTAATGTTCCACGTCTTCTACTACAGAGAGCACGGTAA

>AipsIR14 partial 287 bp

CAGCTTTTTAAACTAATGGAACTCCATAAGCCTTCGCCCAACGGTTCCATGGCATCCACCGTCCGTGGCTACTATAATGGCAGCCTGGTGGATGAAAGACCTCACCGGGAGCTGTTCCGAAGACGCCATAATGTCATGGGACATACCATCACCATGTCCATCGTTATTCAGGATAGCAACACCACGCAGTATCATTTGCTAATGGAGGATAGAATGGAACCCCAATACGACTCCATTGCCAAGATCTGCTGGATGAATGTGAAACTAGCGTTCCAGATGTTGAACGC

>AipsSNMP1 ORF 1569 bp

ATGGCGATGGCAAAGGAGCTGAAGTATGCAGCAATCGCTGGCGGGGTAGCAGTGTTCTGGCTCATCTTTGGGTTCATCCTGTTTCCTGTCATACTCAAGGGGCAGCTTAAAAAGGAAATGGCCCTATCAAAAAAGACGGATGTCCGCAAGATGTGGGAGCAGATTCCGTTCGCCCTGGATTTCAAGGTGTATTTCTTCAACTTTACCAATGCAGAAGAAGTGCAGAAGGGAGCCAAGCCTATCCTTAAGGAGATTGGACCTTACCATTTTGATGAATGGAAAGAGAAGGTGGAAATTGAAGACAATGAGGACGAGGACACTGTCAACTACAAGAAACGGGATGTATTCTACTTCAATCCTGAAATGTCTGCGCCTGGACTGACGGGCGAAGAAATTATTGTTTTACCGCATATTTTTATGATGGCCATGGCACTAACAGTCCACCGAGACAAGCCGGCCATGCTGAACATGATTGGAAAAGCCATGAACGGGATCTTCGACGACCCCCCCGACGTCTTCATGAGGGTTAAAGCTCTGGACATCTTGTTCCGCGGCATCATGATCAACTGTGCGAGGACTGAGTTCGCTCCGAAAGCCACCTGTACTGCTCTGAAGAAGGAAAAAGTTACTGGACTGATTGTGGAGGCTAATAACCAGTTTAGGTTCTCGCTATTTGGGGCGCGAAACGGTTCGATAGATGAGCACGTGATCACAGTAAGGAGGGGTATCAAGAACGTGATGGATGTTGGTAAAGTTATAGCAATAGATGGCAAACCAGAGCAAACAATATGGAGAGACCACTGCAATGAATTCGTTGGCACTGACGGCACTGTGTTTCCACCATTTTTGAAGGAAACTGACCGAATTGAGTCGTTCTCTACTGATTTGTGCAGGCCGTTCAAACCCTGGTACCAGAAGAAGACGTCCTATAGAGGCATCAAGACGAACCGCTACATAGCCAACATCGGGAACTTTGCAGAGGACCCGGAACTCCAGTGCTTTTGTCCTGCACCTGATAGGTGCCCTCCTAAAGGTCTCATGGATCTGGTTCCGTGCATGAAGGCTCCTATGTTCGCCTCCATGCCTCACTTCTTGGACAGTGATCCTGCGTTGCTGGATAATGTGAAAGGGTTGAACCCGGATATCAATGAACATGGGATTGAGATTGATTTTGAACCGATAACAGGTACCCCGATGGTGGCTAAACAAAGAATTCAATTTAATATTCAGCTGCTGAAAACTGATAAGATGGAGCTTTTCAAGGATCTGTCTGGAGATATCGTGCCTTTGTTCTGGATTGACGAGGGTCTGGCTCTGAACAAGACGTTTGTGAACATGCTCAAGCACCAGCTCTTCATTCCGAAGCGTGTGGTGGGAGTGCTTCGTTGGTGGATGGTGTCCTTCGGAAGCCTCGGAGCTGTCATCGGGATCGTTTACCATTACAGAGATCACATTATGCGTCTTGCAGTTTCTGGAGACACGAAGGTCTCCAAAGTAACACCGGAGGATGGTCCTGAACAAAAGGACATCAGCGTGATTGGCCAGGAGCCGGCCAAGATCAACATATAA

>AipsSNMP2 ORF 1563 bp

ATGTTCGGAAAATATGCCAAACTGTTTTTGACTGTGTCCTTGGGATTTTTGGTGGTAGCCATTATTATGGTGGCCTGGGGCTTTGAGAAAATCGTTGACAAACAAATACAAAAGAATGTCCAACTAGAGAACAATTCAATGATGTTCGACAAATGGCTGAAACTGCCCATGCCGCTGGACTTCAAGGTGTATATCTTTAACGTCACCAATGTCGACGAGGTGAACCGGGGGCAGAAACCAATGCTGCAGGAAATTGGACCTTATGTTTACAAAGAATACCGTGAGCGGACGATCCTGGGTTACGGCGAAAACGATACAATCAAGTACATGCTGCGCAAGCGGTTCGAATTCGATCCTGAGGCGTCTGGCGGACTCACGGAGGATGATGAAGTCACTGTCATTCACTTCTCTTATTTGGCTGCCTTGCTGACAGTTCACGACATGATGCCGAGTTTGGTCGGAGTGATCAACAAAGCTCTGGAGCAGTTCTTCCCGAGCCTGGAAGACGCATTCCTGAGGGTCAAAGTGAGGGACCTGTTCTTTGACGGCATATACTTGAGCTGTGATGGTGATAACGCTGCCTTAGGACTTGTATGCGGTAAAATAAAGGGAGAAATGCCACCCACCATGAGAGCAGCGGAAGGAGCAAATGGATTTTATTTCTCAATGTTTTCTCATATGAATCGTTCAGAATCAGGCCCATACGAGATGATCAGGGGTCGCGACAACGTGTACGAACTTGGCAACATAGTCACCTACAAGGGCCAGCAGGTCATGTCAATGTGGGGTGATAAATACTGCGGCCAGATCAACGGCTCCGACTCCTCGATATTCCCTCCGATCAAAGAGAGCAATGTGCCCAAGAAGCTTTATACTTTTGAGCCGGATATCTGCAGGTCCCTTTACATCGATCTAGTGGGCAAGACAGAAATGTTCAACATCAGCTCGTACTACTATGAGATCTCAGAGTCGGCGTTAGCAGCAAAGAGCGCCAACCATGACAACAAATGTTTTTGTAAAAAGAACTGGAGTGCAAACCACGACGGCTGCCTCCTGATGGGTCTTCTGAACTTAATGCCGTGCCAGGGCGCACCAGCCATCGCCTCACTACCTCACTTCTACCTCGCCTCAGAGGAACTGCTTGATTTCTTCCAGTCAGGAATCATGCCTGACAAGGAGAAGCATAAGTCTTATGTTTATATTGATCCGACAACTGGCGTAGTTCTGAGCGGTTACAAGCGGCTACAATTCAACATAGAACTGCGCAAGATTGACAACGTCCCGCAGCTGTCGACCGTGCCCACTGGTTTGTTCCCTATGCTGTGGCTTGAAGAGGGAGCCACAATTCCACCATCAATACAACAAGAACTCCTCGACTCACACAAACTCCTCGGCTACGTAGAAGTCGCTCGATGGTTCCTACTCACGGTCGCTATCATAGCGGTCATAGTGTCAGCGGTAGCGGTCGCGAGGGCCAACGCGCTGCTCTCGTGGCCACGAAACAGTAACTCTGTTAGTTTTATATCAGGGCCCGGCGTTACTATGGTTAATAAAGGAAATTGA

>AipsGR63 partial 720 bp

CAGATTCGTGTAAGTAATAGGGAACTGGCTGTACCGCGACGAAGAAGGCGCCTTTCCCTCATGCAGTCAAAGAACGGACAATCGACGAGCTTCGAAATTAAGAGAATTAGCAAGTGTTACCTTTTATTAACGGAACAAGTGATGTTTGTCAACAAAATGTTCGGTCCTAGGATTCTTCTGAATACCATGAATCTACTTTTGGATATGGTGAAAATATTGAATTTAGCTATAAGAATCACCATTGGATCCCAGCGAACCTTATATCGAGGCGACAATTTAAATTATTTACCGGGAATAACTGGACTAATACGTTTCATGGCGTGTGCTTACATTATAATATCTATGGTATACCAATGTGAGCAAGCATATCGCCAGAGAGAGCAGGTCATGAACGTCATTGATCATCTTCTAATAAACAAAAAGCCTGACGAAGATCTACGAACCGAGATAGTTGATTTGCGGACTTTACTACAAGATCGACCTATAAGCTTCAATATGGAAAATTTCATCACACTGAATTATCCTATTTTTAGTTTCTATTACTTCGGTAGTAGTAACGTATACAATAATATTGTTACAAAATGTAGACTGAAAGTCGTTCAGAAACCCCAGATTCGAAAAAGACTTTTCTTTGCTAAAGCCATGATTATAATCCGTATACCTAAATATTTTTTGACAGTTTTCCAACTATTACTTTTTGTGATTATGGGTATGTATTAA
