# Supplementary material for: Molecular Characterization and Differential Expression of Olfactory Genes in the Antennae of the Black Cutworm Moth Agrotis ipsilon
Source: PLoS One. 2014 Aug 1;9(8):e103420. doi: 10.1371/journal.pone.0103420 (PMC4118888; doi:10.1371/journal.pone.0103420)
Supplement: Table S3 — Primers used for RT-PCR analysis of olfactory genes of the A. ipsilon moth. (DOCX) [file pone.0103420.s003.docx]

| Primer name  imer name | Forward primer (5’-3’) | Reverse primer (5’-3’) |
| --- | --- | --- |
| Odorant binding proteins | | |
| PBP1 | TCGCAGGAGATCATCAAGAA | CCACTTGAGGTCGTGGATCT |
| PBP2 | GCAAAGGGTTCACCAATGTT | CTAAACGGCCGTCATGATCT |
| PBP3 | ATTTGTGTTGATGGCAGCAG | CACTCGCAGACACTCGTCAT |
| GOBP1 | ACAAGTGTCGGCAGGAGAGT | CTCGGTCATCATCATCTCCA |
| GOBP2 | TGTTTGTTGCTGGTTGTCGT | CCGTCAACGTGTCATATTGC |
| OBP1 | ATGCCAAGGATCCAAGTACG | TGTGCGCTGAAACAATCTTC |
| OBP2 | TAGCAGGGTTCATGCAGATG | GATGCGCATTTCTTTCCAAT |
| OBP3 | TTGCTGCTGTGTTTCAGGTT | GGTCCATCTTTGCCCAGTTA |
| OBP4 | CGGCTGCTCTGATCTGTTTT | CACATCCCTTATCACCGTCA |
| OBP5 | ATTTGTTGCCCTGGTAGCTG | CACCAGCGTGGTAACATTTG |
| OBP6 | CAAAGGCGCTTCTTTGAAAC | TTCCATGCTGAATCTGGACA |
| OBP7 | ATTGTGTGTCGTGGCTTTGA | GGCGCTGTCACATTTAGCTT |
| OBP8 | CCTCCCGAGGAGAAAGAAAT | CCCGTTCAGCTAACCAGAAA |
| OBP9 | AAGGCATCATCCTTAGACGAA | TTTGAAGATCAGGGCAGCTC |
| OBP10 | TCCATTTCAGGAAAATAAAACG | TGCCATTAACAAAGGGCACTA |
| OBP11 | TTATCGTCACCGACATCGAA | CGATAACATCATTGCCGTTG |
| OBP12 | TTTTGCTGATTGTGTCAAACG | CGCACTTTGCTGTCCAATAA |
| OBP13 | TTGGTTGCAATGTGTGAAGC | TAAAATATGCCCGGGTTCTG |
| OBP14 | AGCCGATGATGACAAGTTCC | TCTGACGCCGATTCTATTCC |
| OBP15 | ATCGCCATGTTTTTGGCTAC | AATCTTCCGGTTTGAAGTCG |
| OBP16 | TCCTTTTATTTCCGGGTGTG | GTGGTTCAGGATTCCATCGT |
| OBP17 | TAATGGGATGACCCGAGAAC | GCTGGGTCTTTTTCATACAAAC |
| OBP18 | CAGGCTCTGACAGACGAACA | AATCGCATGTTTGGGATCTT |
| OBP19 | CCTTCGTCCTCTGTTTGGTT | TGCTCTCTGGCATTTGTCTTT |
| OBP20 | TACGAAGGCTACGGAACTGG | ATCCGAGAAGCGAAGCATTA |
| OBP21 | GCGGCTGTTAATTTTTGGAA | CAAAACGTCGACTCGTTTCA |
| OBP22 | ATGTGGTTCCGAGCGATG | GAAGTACTCGGCCTTGTTCG |
| OBP23 | AGCAGGGTTTATGCCAATGA | GGCTCTGTGTTCCAAGAAGC |
| OBP24 | ATGTCAGCGACAACGAAAGA | GCAAGTTTCCTTTCGGTCAG |
| OBP25 | AAAGCTTCGTGAGGCTATGC | AGATGAGGATGCTAGTCTGGTG |
| OBP26 | TGTCCAAGTTCACCTGCATC | ACAGACATGCAGGCTTTTCC |
| OBP27 | AGCTGATGCCGAGTCAAGAT | GATGTCGAACGACACGTCAC |
| OBP28 | ATCATCTCGCCGACTGTACC | TTTGTGAGGTCCCCATTGAT |
| Chemosensory proteins | | |
| CSP1 | GCCGTCATAGTCTTGTGTGC | CCGTCAGGGTCGTATTTGTC |
| CSP2 | CTTACGACACCCGGTACGAC | CCGTTAATGAACCTGGTGAAG |
| CSP3 | CTTCATCGTTCTGTGCATCG | TATTTGGCGGTGAACTTCCT |
| CSP4 | TCACCCTTTGTTTCGCTCTC | GCCTCTTGGTATTTGCCTTG |
| CSP5 | ACGCGCTATTGTTATGTTGC | CGTACTGGCGAACTATTTTGG |
| CSP6 | GCTGATAATCGCAGTTGCTTT | TCGACAACTCCTTCCACAAG |
| CSP7 | TGTGTGTCATGGTGGCTGTA | CTGGCACGGTCTTCGTACTT |
| CSP8 | TTTTGTGGCAGCTGAAACTT | GCCAGGAAGGCTTCGTAGTT |

**Table S3.** Primers used for RT-PCR analysis of olfactory genes of the *A. ipsilon* moth.

**Table S3.** Continued.

| Primer name | Forward primer (5’-3’) | Reverse primer (5’-3’) |
| --- | --- | --- |
| CSP9 | TCCTGAACAATGAGCGACTG | GCGGCATCCTTAGTGTCTTC |
| CSP10 | ATGAAGGCCGAATGTGTTCT | TCGTACTTATCGGTGATCTGCT |
| CSP11 | AATTCTTCCCGAAGCCCTAA | TTATTTCGCCGTCTCCAAAC |
| CSP12 | GGCATTCGCAGCTGATAAGTA | TCTGCTTCCCTGGATGAACT |
| Odorant receptors | | |
| ORco | GTGCATGGCTATCAACATGG | CACCTCCGTCAAGGTCTCAT |
| OR1 | GTGGCTCTTTTGGGTGACAT | TTTCCTGTGCGTCTTCATTG |
| OR2 | CGAACTATAGCTCCGGCAAG | CCGACCCTCATGCTACTCAT |
| OR3 | CTCTCAAATGAACCGGCAGT | CTCCGCAACTGTTTGTAGCA |
| OR4 | TGGAAGCTGGTCACATGTATCT | TTGAGTTCCTCCGCTTTTGT |
| OR5 | AGAACTTGACGGAGCGACAG | GGCCAGGCAGTGATTATTGT |
| OR6 | GCCAGGATCATGTTCACCTT | ATATCGCCACCATTCCACAT |
| OR7 | TCAACCACGCTTGTCTGAAG | CAGTCGCTCTGATGTTTCCA |
| OR8 | ATTTCCGGTCTGCAGTTACG | GCAAAACTTGCCCACTTCAT |
| OR9 | TGGCCGTGGGTTACTTCTAC | GGGAAGTAGCAAAGGCTTCA |
| OR10 | TGCATTCAACTGTCCATCGT | CACGGTGTTCTCATCAATGG |
| OR11 | AGCCAAGAGTACGACGATGC | ATGCAAGTCCATGTGCAGAA |
| OR12 | TATCTTCGGCAGGTTTGGTC | ATATCGCATCGCTGACTTCC |
| OR13 | GGCATTGGCATTCATTTTCT | GAGATTGCATGTGACGCAGT |
| OR14 | CAAGCAAATTCACCGAATCTC | TCGATTGCATTGCTTTGTTC |
| OR15 | ACGAGCAGGACACCAAGTTC | CAGTGTTCACCCGGTTTAGG |
| OR16 | GTCGCTTTCTCAAGCGTCAT | AACACAGTGTGCAGGTCAGC |
| OR17 | TTCATGATGATCGCTGGTGT | TACCTGCATCAACGCAAAAG |
| OR18 | CCAAACAGGAACACCGCTAT | CCATGCGTCTCTCGTAGTCA |
| OR19 | TACACCTGCTGTGCCAGTTC | CCTCGCCTATTCTTGCACTC |
| OR20 | GGTGCTCATAGGCACTGGAT | CAGCACTGAAAACACGGAGT |
| OR21 | TGCTGTTTTCCTTCGTCTGA | AGTAGCCCTGCAGGTTTTTG |
| OR22 | ACCTATAGCATTCACAGCGATGG | TTCACTGTACATCGTATACAGTAATGC |
| OR23 | CTCATGATTCCGAAGCTGGT | GTCAAACAAACCACGGGACT |
| OR24 | TGGGACGGAGACTTACTTGC | AGAACCTTTGCATTGGGTTG |
| OR25 | AGCTTCGAGCTACCCATTCA | GTCGACATGGACGAGGCTAT |
| OR26 | GGTGTTAACAGCGTGTTTTGC | CTTGCATGCTCGATGTCCTA |
| OR27 | TGCATCTTGACGGAGTTGTT | ATCAAGTGAGAGCGGGATGA |
| OR28 | GCAATTCCAGAAGAGATTGAGA | CTGGCGATCACCACCATTAT |
| OR29 | GGAAAGCTGGACTTGCTACG | GTGGTTCGCCAAATACCAGT |
| OR30 | TGAACAACACGCTCAGGACT | TTCCCACAGCGGTCTTAATC |
| OR31 | TCCAATGAAAGCACCATGAA | CGTCGCCATCTCAGTTTCTT |
| OR32 | TGGGCACCAGAATATTTGTC | TCAGAGGCAGTTGTTGCTTC |
| OR33 | TGCACACTTTGGAGATAGGG | CGTAGGCCTTCCCTTAGTGAC |
| OR34 | TTGAACATGAAATCCGCAAG | ACCACGACCCAGTACAGCAT |
| OR35 | TTTGTCTTCGTGAGGTGCTG | GAGGTGACGTTGGCTGAAAT |
| OR36 | TGTTGAAGCCGAAGATAACAAA | TCGCTGTAGTAACAAGGCATGTA |
| OR37 | TGAAATTGGAGTGTTGAACAAA | CAGCATCACACTCCACAACA |

| Primer name | Forward primer (5’-3’) | Reverse primer (5’-3’) |
| --- | --- | --- |
| OR38 | AGGGGAGATCAGCTTGGAAT | AAAGATCAGCCAGTGCATCC |
| OR39 | AAACGTGACTCGGTGAGCTT | AGCCATAAACGATCGAAACG |
| OR40 | TTTGCTGGAATTCGCTTTTT | CTCTTGGGAAATCAAACACCA |
| OR41 | ACGACCACAGTTGGTGGTTT | AAAGCTCCCGTAACTGATCG |
| Ionotropic receptors | | |
| IR8a | CGTCAATGGTTTTGTCATGG | GAGACCGGACCTGTCAACAT |
| IR25a | GTCATTGGAGAAGGGCGATA | ACCCAGCGAGTGGCTACTTA |
| IR21a | GATACGCTGGTCATCGGTTT | TCCGAGAAAGCTAACGGAAA |
| IR41a | ACCGGTTGGTTGTTGATAGC | CCAGTTGGCTGACTTTCTCC |
| IR75q.1 | AATTGAGGGCCAACAATGAA | TATTGGTGTCGGGATTGTCA |
| IR75q.2 | CGATGGATTCTTTTCGGAGA | AGTGCCATTGACCCGATAAC |
| IR75p | AAGGAGTGGAGCGAATCAGA | ATTTTGGAGGGTTTCGCTCT |
| IR76b | GAATTTGGCGAAGGAAGTCA | GAACTGCTGCCAGGACTAGG |
| IR87a | TTTTCGGAGGGTGTAAGACG | TGGATGCTCAAAGGCTCAAT |
| IR93a | AGAATTGGCAAATGACACGA | TGAATGGAAGTAGAAACAGCAGA |
| IR1 | AATCGGCCTGACAAGTATGC | CTCTTCTGCGCCATGATGTA |
| IR2 | GATGCCCACACTTTGGATCT | GCTTCTGGACTCCACTTTGC |
| IR3 | GTACAACCAGCCCCTAAGCA | CAGCAGGAGAGCAGCACATA |
| IR4 | GGCAGCAGAGATCAACAACA | GGTGCTCATGAGTGCAGAAA |
| IR5 | GGCGGAGTATTCATTGTGCT | AGATCCACCTGATCCACTGC |
| IR6 | ATGCCATCCTCATGTGTCAA | GAAGCGTAGACGAGGGTCAC |
| IR7 | TATCACCGTAATGGCCACAG | TCTCCTCCCAAAGCAAAGAA |
| IR8 | TGCATAACGATTATTTCGGATG | AACACCAGTGAAGGCAGGTC |
| IR9 | AGGGTTACGCCATTGATCTC | GAACAAGTTCGGTGGTTGCT |
| IR10 | TGACGTCCCGAAGTCCTATC | ATGCACAGTGTCGACCTCAA |
| IR11 | CGTACCATCGACGACCTCAC | CCTGAACTCCTTTCCACCTG |
| IR12 | AATCAACAACCGCAACATCA | GACGTAAACACAAGCGAGCA |
| IR13 | TTCCCTGAGAAAGCGAAAAA | TATGACAGCGTAAGCCACCA |
| IR14 | TGGAACTCCATAAGCCTTCG | GATCTTGGCAATGGAGTCGT |
| Sensory neuron membrane proteins | | |
| SNMP1 | GACTGAGTTCGCTCCGAAAG | AGTTCCCGATGTTGGCTATG |
| SNMP2 | TCTTATTTGGCTGCCTTGCT | TGATCGGAGGGAATATCGAG |
| Gustatory receptors | | |
| GR63 | TCCCAGCGAACCTTATATCG | TCTGGGGTTTCTGAACGACT |
| Reference genes | | |
| β-actin | ACCACACCTTCTACAACGAGCTG | AGCGCGTATCCCTCGTAGATG |

**Table S3.** Continued.
