# Supplementary material for: Molecular Characterization and Differential Expression of Olfactory Genes in the Antennae of the Black Cutworm Moth Agrotis ipsilon
Source: PLoS One. 2014 Aug 1;9(8):e103420. doi: 10.1371/journal.pone.0103420 (PMC4118888; doi:10.1371/journal.pone.0103420)
Supplement: Table S4 — Primers used for RT-qPCR analysis of olfactory genes of the A. ipsilon moth. (DOCX) [file pone.0103420.s004.docx]

| Primer name | Forward primer (5’-3’) | Reverse primer (5’-3’) |
| --- | --- | --- |
| Odorant binding proteins | | |
| PBP1 | CAGGAGATCATCAAGAAT | CCAGAAGTTGTAGAAGTC |
| PBP2 | ATAGGAGAGGACCAGAAG | ATTCGTAGAGAATGGCTA |
| PBP3 | TTCAGGGTTCGTTAAGGT | CGTGTAGTCCAACTTCCA |
| GOBP1 | CAAGTGTCGGCAGGAGAG | TCGGTGAGCAGGTTGAAG |
| GOBP2 | CTGTATGTCCAACAAGTT | AGAGAACCTCACCATTAG |
| OBP1 | TTAGAAGATTGCTGGTTA | TCATTTTCGTACTTGGAT |
| OBP2 | AGTTCACTTGTTTGGTTT | CAATGTCATCGTTGCTAA |
| OBP3 | ACTAATGCCAAGAAGGTA | GCTTACAGTTCCATAATACA |
| OBP4 | GGACAAGACCAAGGAATT | ACTTCTTCATCGTTCACTT |
| OBP5 | TATTAGCCGAAGCGAAGA | TTCAACTTGCCATCATTGT |
| OBP6 | AAAGTGTTCCAACCCTAA | CTTCCTTGTTCAGAGTATTC |
| OBP7 | CGAGATGATGAAGAATATACTAA | AAAGCAAAGAGACAAACG |
| OBP8 | TGCTAAGCCGTTCGTAGA | GATGATTCTGTACTTCTTGATGGA |
| OBP9 | GCTCAAGACCAAGACAAT | TCATTCACACCTTCAACTG |
| OBP10 | TTCATTTCAGACCCATTT | GTATGCCATTAACAAAGG |
| OBP11 | TATTCGCTGACGGTATTC | TCACAATTCTTGAGTCCTT |
| OBP12 | GATCAGACAAGTGGATATG | ATAAGATGCCTCACAGAT |
| OBP13 | TTGACGAGCCAGAAGTAA | TCATCAGGTAGCAATAAATCAG |
| OBP14 | GAGTACAACAATAATCAGAT | GGTCATATCCACTTCTAA |
| OBP15 | AATAAGCATCAAGTTACAGAAGAC | TCGCAAGGAGGATTCGTA |
| OBP16 | AATCCAACGACTGCTTCTT | AATGCTCAACGATAGGCTTA |
| OBP17 | CAATGTTATGTGCTATGTC | CCTTCTTAGTAGGCTCTT |
| OBP18 | GACGAACAGAAGGAGAAG | TTCAGTCTTGTAATCACCTC |
| OBP19 | AGAGGAGGATATAACGAAT | ATAGTCCACAGTACCATC |
| OBP20 | AGATTACGATTACGAAGG | CGACTTGTATCATTATTGTT |
| OBP21 | TATCGGAATACACAGGAGAC | TCTTCATCATCACCATCATTATC |
| OBP22 | GCTCAAGTGCTACATCAAGTG | GTTGTGCTCCGCCATCTC |
| OBP23 | TTCAACAGCAAAGGAGATTTC | TTCCAACTTCAGCCACTT |
| OBP24 | CCTACATACAAGCACTGAA | ACCATCCTCCGATACAAT |
| OBP25 | AGGATGTCAACCAGTATA | TTAGATGAGGATGCTAGT |
| OBP26 | GAAGGCAGAAGGAGTAAG | TTCATTCAACGCATCAGT |
| OBP27 | ATGAAGAACCACGACAAG | CGATACGGTCCGAAATAC |
| OBP28 | TCTCTTCTTCGGTCATCAT | TCTTGTAAGTCCGTGTCA |
| Chemosensory proteins | | |
| CSP1 | CCAGAGGAAGAGAAGTAT | CAAGTAAGCAGTCACAAT |
| CSP2 | TCAAGGGATTCCAGACTA | TTAATGAACCTGGTGAAGAT |
| CSP3 | AACAACGAGGCTGACTAC | TTATTGCTTGATCTCTTTGAGTT |
| CSP4 | AAACTCGTTGTGCTAAATGTA | ACCAGTTCCTTCCAGATG |
| CSP5 | CCTGTGATCCTATCGGAA | CGCTGTATCTGCTTAGTT |
| CSP6 | AGTTATCAGGCACTTAGT | ATTTGGGTCTTGTATTTGT |
| CSP7 | AGTTCGTCTTGCTATTGTG | TGTTGGTCAGGATCTCAT |
| CSP8 | AAGATGTACTCCTGAAGG | TGATGTGCTTGACTACTT |

**Table S4.** Primers used for qRT-PCR analysis of olfactory genes of the *A. ipsilon* moth.

**Table S4.** Continued.

| Primer name | Forward primer (5’-3’) | Reverse primer (5’-3’) |
| --- | --- | --- |
| CSP9 | ACTACCTTATAGATCACAGA | CCTTAGTGTCTTCATCAG |
| CSP10 | AGAAGCAGTTAATCAAGA | TTATATGTCCTGTCCTTATC |
| CSP11 | CAGTGCTATTATTGTGTGTTT | CGCATCAATGTCCAGATA |
| CSP12 | TGATAGTGTTGTCTTGTCT | GAGAAGTCTGTCATTGGA |
| Odorant receptors | | |
| ORco | CTACCAAGCAACTAAGAT | TCCATTACTGATGAACTC |
| OR1 | AAGATGTGTCTGAATATGC | AGAAGAATAGTTGCTCCA |
| OR2 | CTTCGTTGTTGATATTAGC | TTGTTCGTAAGTCCTATTC |
| OR3 | TAACAGAGAATATCCACTACC | TCCAACATCAGAAGACATT |
| OR4 | ACAATAGCGAACTTAACTT | TGGTAGAACAGATGGATT |
| OR5 | TTGCTTCTCTTCATCATC | ATCTCCTTCAGTCCTATC |
| OR6 | AACAATGCTGGCTATCTG | TTCATACTTCACTTCCTTCTC |
| OR7 | CGACTATCATCTTCTCAAC | AATCGTCACAGTATTAACC |
| OR8 | ATGTCCTATTCTCCGTTA | CATCTAATATCGCAATGATT |
| OR9 | TACAATCTTCTCCGTCTA | TATTCTTCAGCATCTTCC |
| OR10 | TGAAGATGAACGATGATT | TACAAGCCACCTACTATA |
| OR11 | AGGTTACTCTGTATCTGT | ACACGAACATATCTAACG |
| OR12 | GCTTGAATTACTCTTACCAT | ATCCATAACCACGACTAC |
| OR13 | ATACTGTTGTGATGACTC | CATATTGCTCTTGAATCTG |
| OR14 | CAAGGAACATTCTGAATT | GACCAGTTATTGTATATCG |
| OR15 | ATGGAACGGATGAAGAAA | CTGCGAAGTAAGAGTATGA |
| OR16 | GCCGACAACTACAACAAG | AACAGCAGATATGGATGGT |
| OR17 | TATAACGCAGAAGTGAAGA | CTTGATTGAGCAGATTGAT |
| OR18 | CTATCAAGAGATGTGTTACTAT | TAGATGTCCAAGGTGTAG |
| OR19 | TAATAGACGAAGTGGACTA | GTGCGAGATGTAATAGAA |
| OR20 | GCACTGCGATATATTTCT | CTATGTATCCGAGACCTT |
| OR21 | CCTCAGCCATCAGTACAA | TTCATACATCTGCTCCTTCT |
| OR22 | AACCTATAGCATTCACAG | GTACATCGTATACAGTAATG |
| OR23 | TGTTGCGTATTGTATTCCTA | TAGTGGCTGGATGTATGT |
| OR24 | GCCTATCACCAATAATGT | TATTCAGTCGTCAAGAAC |
| OR25 | ATATTCTATGACCATTCTTG | TAATGTCTTCCTCTACTG |
| OR26 | TGACTAACATTATGAGGAAGA | CGTTGAATGGATAGTATGC |
| OR27 | GCATCTTGACGGAGTTGTT | CTCCATGACCAGCACCAG |
| OR28 | TTAATCATCTGCTTGGTC | AAGAGAACATTGTGTAACT |
| OR29 | ACAACTTCACGACATACAT | AAGCATCAAGGAACACAT |
| OR30 | CTACAACTTACTTGGTGAA | GTCTTAATCTTGAACTCCTT |
| OR31 | GCACCATGAACCTGATAT | TTGATCGCACAAGTACAT |
| OR32 | ATTGTCAGTGAGCATATC | TTGGATGATTAGGTAGGT |
| OR33 | ACTGTAGGATTGTTATGG | CCTTAGTGACTTCATTGT |
| OR34 | AATGATGATAACCTTACG | GTCTATATTGACAGTGAG |
| OR35 | GAGAATTATGAATGAACAC | CTGATACCAATCCTATAAC |
| OR36 | ATTACCACTAACAACATTCCA | GTCGCTGTAGTAACAAGG |
| OR37 | GCGTCTTGTATGTATGTTG | TATAACTTCAGCATCACACT |

**Table S4.** Continued.

| Primer name | Forward primer (5’-3’) | Reverse primer (5’-3’) |
| --- | --- | --- |
| OR38 | ACTACTATAGCACTGATA | GATTAAATATGGCGTAAG |
| OR39 | GTCAGTCACACGATAGTTA | CTTATATTTCTTCGCCATCAA |
| OR40 | GACAACAGGAGTCATCAT | ACCATTATCCAACAGTGAA |
| OR41 | TCCTAATGATGAGTGTCTT | GCTCTGATCGTTATTGTC |
| Ionotropic receptors | | |
| IR8a | ATGGACACTTATAGAAGG | GGACTAACTGACTAACAT |
| IR25a | TGATTAACGAGGAGAGTAA | CCACAACAATAACAGGAT |
| IR21a | AACACAGATTGCCAACATT | TGCGATGACCTTGCTATT |
| IR41a | GGTATATGTGGATTGCTGTA | TGTTCTGGTATTGAGATTGT |
| IR75q.1 | AATATCCTCGTAGACACA | GCCATCAGTTAAGTATCC |
| IR75q.2 | TATATCTTGTGCTGCTAA | GATAACATCAACGACATT |
| IR75p | TGGAGATACTGAGAGTTG | CTGCTGAATGAAGACATC |
| IR76b | AATAAGATGAATGATGACTC | CAGGTCTATTGAGAATGA |
| IR87a | CGTCAAGCCATCTACCAA | CCTCGTCAGAATAGCAACT |
| IR93a | CTGTTCTATCTGATCCTAT | GTAGTGAATGGAAGTAGA |
| IR1 | GTGTTTGCTGCTATATGC | CCGATTACCAGAAGAGTC |
| IR2 | TTCCAGGACAATCTTATG | TCTTCAGAATCACAACTAA |
| IR3 | ATTATGGATAGATGGATTGGTA | CTTGAACGGACTTGAGAA |
| IR4 | CTTCTTGTCAGTAGTCTT | TACATCCTCAACATCTTC |
| IR5 | TCCGCAGTTGTTATTGTTA | GCCAGTAGATTCTCTATGAC |
| IR6 | TTCAACGAACAGTCCATC | AACAGGAACAGAGCAATG |
| IR7 | TGATAACTACACAATGAC | TTAAGGTGAATATCTAAGC |
| IR8 | GTAAGGAGAAGTGATAGG | TATATTCGCAACATAACTG |
| IR9 | CATTACATACGACAGAGA | GATAGGAAGGAGAACAAG |
| IR10 | AGTTGGAATGGTGATAGA | CCGTAGTTAATGACAGATT |
| IR11 | GCTGTACCGTCGCAAGAT | CCTGAACTCCTTTCCACCTG |
| IR12 | AACCGTATAGTATCAACAA | CTCAGCGACTAATATCAA |
| IR13 | TAATGCCTCCAGTAAGTAA | TCTTGAATCTCCGATGTT |
| IR14 | GTTCCGAAGACGCCATAA | TTCCATTCTATCCTCCATTAGC |
| Sensory neuron membrane proteins | | |
| SNMP1 | TGGTGGCTAAACAAAGAA | CTCGTCAATCCAGAACAA |
| SNMP2 | TAGGACTTGTATGCGGTAA | GCCTGATTCTGAACGATT |
| Gustatory receptors | | |
| GR63 | ATGTGAGCAAGCATATCG | TCAACTATCTCGGTTCGTA |
| Reference genes | | |
| β-actin | GATGTTGACATTCGTAAG | ATCTTGATCTTCATTGTG |
| RPS3 | AAGTCCATGAAGTTCGTA | GTACTCCTTGTCTTAGCA |
